# Presynaptic targeting of botulinum neurotoxin type A requires a tripartite PSG‐Syt1‐SV2 plasma membrane nanocluster for synaptic vesicle entry

**DOI:** 10.15252/embj.2022112095

**Published:** 2023-05-25

**Authors:** Merja Joensuu, Parnayan Syed, Saber H Saber, Vanessa Lanoue, Tristan P Wallis, James Rae, Ailisa Blum, Rachel S Gormal, Christopher Small, Shanley Sanders, Anmin Jiang, Stefan Mahrhold, Nadja Krez, Michael A Cousin, Ruby Cooper‐White, Justin J Cooper‐White, Brett M Collins, Robert G Parton, Giuseppe Balistreri, Andreas Rummel, Frédéric A Meunier

**Affiliations:** ^1^ Clem Jones Centre for Ageing Dementia Research, Queensland Brain Institute The University of Queensland Brisbane QLD Australia; ^2^ Queensland Brain Institute The University of Queensland Brisbane QLD Australia; ^3^ Australian Institute for Bioengineering and Nanotechnology The University of Queensland Brisbane QLD Australia; ^4^ Institute for Molecular Bioscience The University of Queensland Brisbane QLD Australia; ^5^ Institut für Toxikologie Medizinische Hochschule Hannover Hannover Germany; ^6^ Centre for Discovery Brain Sciences, Hugh Robson Building University of Edinburgh Edinburgh UK; ^7^ Muir Maxwell Epilepsy Centre University of Edinburgh Edinburgh UK; ^8^ Simons Initiative for the Developing Brain University of Edinburgh Edinburgh UK; ^9^ School of Chemical Engineering The University of Queensland Brisbane QLD Australia; ^10^ UQ Centre for Stem Cell Ageing and Regenerative Engineering The University of Queensland Brisbane QLD Australia; ^11^ Centre for Microscopy and Microanalysis The University of Queensland Brisbane QLD Australia; ^12^ Department of Virology, Faculty of Medicine University of Helsinki Helsinki Finland; ^13^ School of Biomedical Sciences The University of Queensland Brisbane QLD Australia

**Keywords:** BoNT/A, endocytosis, polysialogianglioside, SV2, synaptotagmin‐1, Membranes & Trafficking, Neuroscience

## Abstract

The unique nerve terminal targeting of botulinum neurotoxin type A (BoNT/A) is due to its capacity to bind two receptors on the neuronal plasma membrane: polysialoganglioside (PSG) and synaptic vesicle glycoprotein 2 (SV2). Whether and how PSGs and SV2 may coordinate other proteins for BoNT/A recruitment and internalization remains unknown. Here, we demonstrate that the targeted endocytosis of BoNT/A into synaptic vesicles (SVs) requires a tripartite surface nanocluster. Live‐cell super‐resolution imaging and electron microscopy of catalytically inactivated BoNT/A wildtype and receptor‐binding‐deficient mutants in cultured hippocampal neurons demonstrated that BoNT/A must bind coincidentally to a PSG and SV2 to target synaptic vesicles. We reveal that BoNT/A simultaneously interacts with a preassembled PSG‐synaptotagmin‐1 (Syt1) complex and SV2 on the neuronal plasma membrane, facilitating Syt1‐SV2 nanoclustering that controls endocytic sorting of the toxin into synaptic vesicles. Syt1 CRISPRi knockdown suppressed BoNT/A‐ and BoNT/E‐induced neurointoxication as quantified by SNAP‐25 cleavage, suggesting that this tripartite nanocluster may be a unifying entry point for selected botulinum neurotoxins that hijack this for synaptic vesicle targeting.

## Graphical abstract

Live‐cell super‐resolution imaging and electron microscopy of botulinum toxin type A in hippocampal neurons reveal the mechanism for its synaptic vesicle hijacking and neurointoxication.

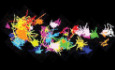

## Introduction

The extreme potency of clostridial neurotoxins (CNTs), including botulinum neurotoxins (BoNTs) and tetanus neurotoxin (TeNT), is mediated by their high affinity and specificity for peripheral cholinergic motoneurons and, in the case of TeNT, retrograde axonal transport to the inhibitory interneurons of the central nervous system, which leads to deadly paralysis (Gill, 1982; Rossetto & Montecucco, 2019). All BoNT serotypes and TeNT share a similar molecular structure consisting of a heavy chain (HC, 100 kDa), which is connected to a light chain (LC, 50 kDa) by a disulphide bond and noncovalent interactions (Lacy *et al*, 1998; Swaminathan & Eswaramoorthy, 2000; Montal, 2010; Pirazzini *et al*, 2017). According to the dual receptor model (Montecucco, 1986), CNTs achieve their high affinity and specificity for neurons by binding to two plasma membrane receptors. The HC first binds to a polysialoganglioside (PSG) with low affinity, followed by high‐affinity binding to one of the two synaptic vesicle (SV) proteins, either synaptotagmin‐1/2 (Syt1/2) or N‐glycosylated SV glycoprotein 2 (SV2).

BoNT serotypes A, B, E, F, and G share a conserved PSG‐binding site, whereas BoNT/C, /D, and /DC display two independent PSG‐binding sites (Rummel, 2013). PSGs, such as GD1a and GT1b, comprise a large family of glycosphingolipids that are abundantly expressed on the outer leaflet of the presynaptic plasma membrane and organized in microdomains together with a number of glycoproteins. While the PSG‐bound toxin has been suggested to move laterally to find and bind to the less abundant protein receptor (Rossetto & Montecucco, 2007), the precise role of the PSG binding of the toxin is not well understood. Binding to the protein receptor, on the other hand, is serotype‐dependent and mutually exclusive: BoNT/A, E, and TeNT bind to SV2, whereas BoNT/B, /DC, and /G bind to Syt1/2 (Rummel, 2017) as a requisite step for endocytosis into SVs. Independent of which receptor the toxin binds, the end result is similar; following the endocytic targeting of the toxin into SVs and acidification of the SV lumen, the LC of the toxin translocates into the cytosol where its Zn^2+^‐endopeptidase activity cleaves its specific target, soluble N‐ethylmaleimide sensitive factor attachment protein receptor (SNARE), thereby blocking neurotransmission and causing paralysis (Foran *et al*, 2003; Dong *et al*, 2006; Osborne *et al*, 2007).

Syt1 and SV2 receptors are, therefore, remarkably complementary in the SV targeting of CNTs. BoNT/A binds to all three SV2A‐C isoforms (Dong *et al*, 2006; Strotmeier *et al*, 2014). SV2A and SV2B exhibit broad expression in the central nervous system while SV2C is more restricted to defined areas such as the striatum (Crevecoeur *et al*, 2013). SV2 is an intrinsic trafficking partner (iTRAP) for Syt1, ensuring that these SV proteins are clustered and retrieved efficiently from the neuronal plasma membrane (Yao *et al*, 2010; Kaempf *et al*, 2015; Zhang *et al*, 2015; Harper *et al*, 2020). Syt1‐SV2 iTRAP interactions (Gordon & Cousin, 2016; Bartholome *et al*, 2017), and the ability of Syt1 and SV2 to form nanoclusters at the plasma membrane (Small *et al*, 2022), are considered to function as a “fail‐safe” mechanism, which ensures that SVs are formed with the correct molecules at the required stoichiometry. BoNT/A‐HC has been shown to be internalized into uncoated SVs and clathrin‐coated vesicles (Couesnon *et al*, 2009; Harper *et al*, 2011; Colasante *et al*, 2013; Pellett *et al*, 2015), and both Syt1 and SV2 have been shown to interact with clathrin adaptor proteins. Syt1 has been demonstrated to function as a dual Ca^2+^ sensor for both endo‐ and exocytosis (Yao *et al*, 2011; Chen *et al*, 2022), and to specifically facilitate clathrin‐mediated endocytosis, and clamp bulk endocytosis (Yao *et al*, 2012; Chen *et al*, 2022). The cytosolic C2B domain of Syt1 has been shown to bind to endocytic adaptor protein 2 (AP‐2) and stonin‐2, further supporting a role for Syt1 in clathrin‐dependent endocytosis (Zhang *et al*, 1994; Haucke *et al*, 2000; Grass *et al*, 2004; Walther *et al*, 2004; Diril *et al*, 2006). SV2A has been shown to bind to multiple clathrin‐dependent endocytosis proteins such as AP‐2, epidermal growth factor receptor pathway substrate 15 (EPS15), and amphiphysin 2/bridging integrator 1 (Bin1) (Yao *et al*, 2010). SV2 and stonin‐2 play a major role in regulating the amount of Syt1 in SVs (Yao *et al*, 2010; Kaempf *et al*, 2015).

Because of their small size, Syt1 and SV2 nanoclusters and the nanoscale organization of BoNT/A can only be detected in live neurons using super‐resolution single‐molecule tracking which allows the investigation of the neurointoxication process with high spatiotemporal resolution. One of the major limitations of published work on the binding, endocytosis, and trafficking of BoNT/A using live‐cell imaging has been the reliance on atoxic 50 kDa receptor‐binding domains (Hc fragments) at concentrations far exceeding pathophysiological levels. Here, we took advantage of single‐ and dual‐color super‐resolution imaging, in particular live‐cell single‐molecule tracking, in cultured hippocampal neurons to fully characterize the intoxication mechanism of intact BoNT/A at picomolar concentrations from plasma membrane binding to selective endocytosis into SVs. Using catalytically inactivated BoNT/A wildtype and receptor‐binding‐deficient mutants, we demonstrate that PSGs not only serve as low‐affinity binding sites but also recruit the endocytosis factor Syt1 in the BoNT/A neurointoxication process, which leads to efficient internalization of the toxin into the correct endosomal compartment. We also show that the coincidental binding of the toxin to a preassembled PSG‐Syt1 complex and SV2 elevates the interaction between Syt1 and SV2, as well as the number of nanoclusters that the two receptors form on the neuronal plasma membrane. By actively promoting the SV2‐Syt1 complex, BoNT/A hijacks the existing SV protein endocytosis mechanism to facilitate its own endocytic targeting into SVs. Using electron microscopy (EM), electron tomography, and electrophysiology, we demonstrate that Syt1 knockdown (KD) leads to mistargeted endocytosis of the toxin and a reduction of BoNT/A neurotoxicity. Reintroduction of wildtype Syt1 following Syt1 KD restores BoNT/A toxic function, whereas a Syt1 mutant with disrupted interaction with PSGs does not. We also reveal that perturbations of PSG‐Syt1‐SV2 intermolecular interactions lead to a major deficiency in BoNT/A endocytosis and mistargeting of the toxin into a degradative endocytic pathway. Syt1 therefore plays a central role in BoNT/A intoxication, by hijacking the tripartite PSG‐Syt1‐SV2 complex as an intrinsic host endocytic sorting mechanism. Finally, we demonstrate that BoNT/E, which selectively binds to PSGs and SV2, also relies on a preassembled PSG‐Syt1 complex to intoxicate neurons, suggesting that PSG‐Syt1‐SV2 tripartite nanoclusters may act as a unifying entry point for other BoNT serotypes.

## Results

### Coincidental binding to PSG and SV2 is a requisite for endocytic targeting of BoNT/A into SVs

Due to the extreme toxicity of the LC, we previously used BoNT/A‐H_C_ to study the endocytic uptake and trafficking of the toxin (Wang *et al*, 2015; Harper *et al*, 2016). However, a detailed analysis of the intoxication mechanism of the intact holotoxin using super‐resolution imaging was not conducted. Single‐molecule imaging enables visualization of individual neurotoxin molecules in pico‐ to nanomolar concentrations as they bind to their plasma membrane receptors in live neurons, thereby initiating their intoxication journey. To investigate the BoNT/A neurointoxication process in detail, we created catalytically inactivated holotoxins (E224A/R363A/Y366F; Gu *et al*, 2012; BoNT/Ai^wt^) and introduced additional mutations to abolish BoNT/A binding to either PSG (W1266L; Rummel *et al*, 2004b; BoNT/Ai^PSG^), SV2 (G1141D/G1292R; Strotmeier *et al*, 2014; Yao *et al*, 2016; BoNT/Ai^SV2^), or both (W1266L/G1141D/G1292R; BoNT/Ai^PSG,SV2^; Appendix Fig S1A; Movie EV1). For single‐molecule imaging, the holotoxins were labeled, on average, with 1–3 Atto647N fluorophores (BoNT/Ai‐At647N; Appendix Figs S1 and S2A; Movie EV1). Similarly labeled catalytically active BoNT/A retained high potency (~ 20%) relative to the nonlabeled BoNT/A in *ex vivo* mouse phrenic nerve assays (Appendix Fig S2B–E), indicating limited interference of the At647N labeling with the neurointoxication process.

**Figure 1:**
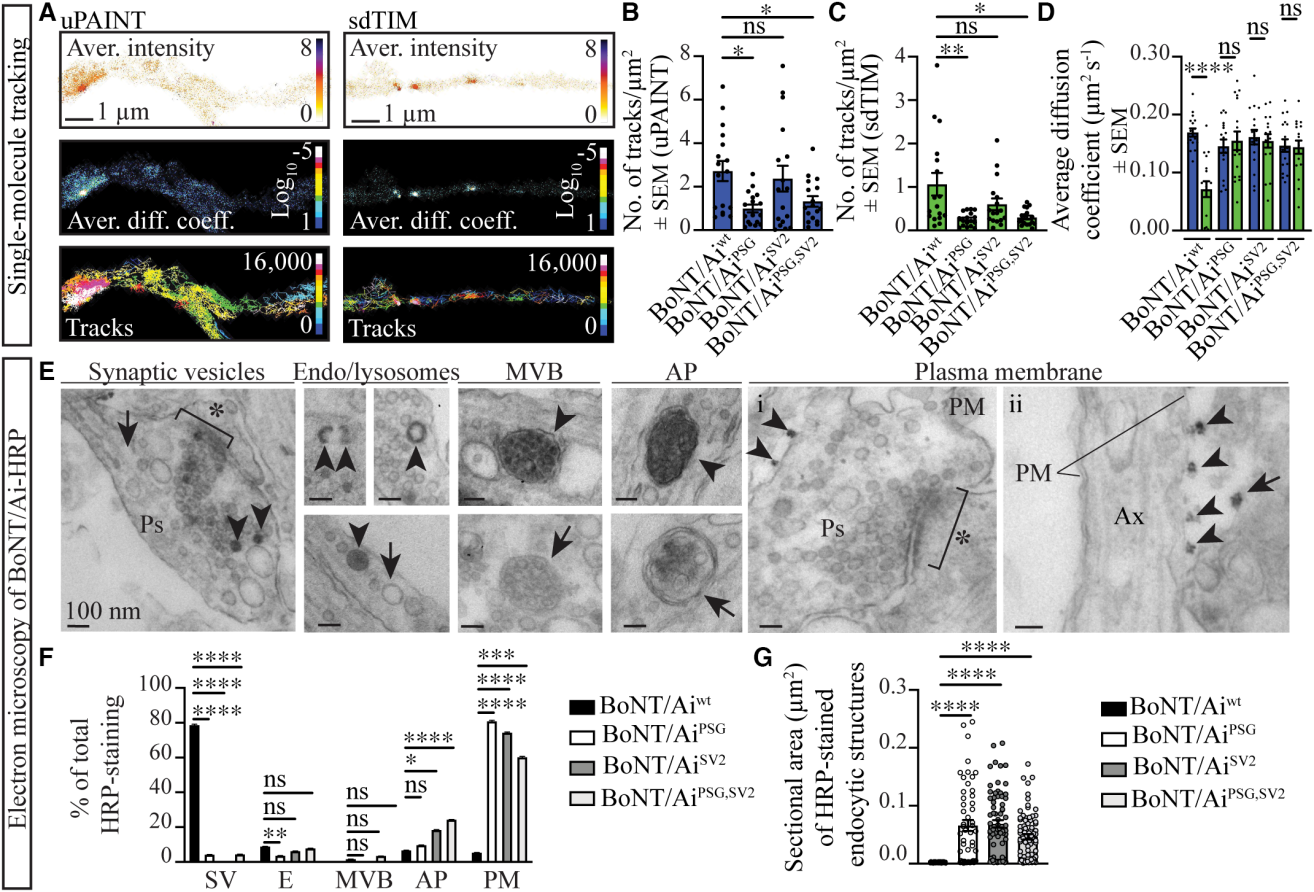
BoNT/A must coincidentally bind to PSG and SV2 on the neuronal plasma membrane for endocytic targeting of the toxin into SVs ASuper‐resolved average intensity (bar: high 8 to low 0 density), average diffusion coefficient (bar: Log_10_D high 1 to low −5 mobility), and trajectory maps (16,000 frames) of BoNT/Ai^wt^‐At647N on the plasma membrane (uPAINT) and following endocytosis (sdTIM).B, CNumber of BoNT/Ai^wt^‐At647N and mutant trajectories (B) on the plasma membrane (uPAINT) and (C) following endocytosis (sdTIM).DScatter plot of the average diffusion coefficient (μm^2^ s^−1^) of indicated toxins following uPAINT (blue) and sdTIM (green) single‐molecule imaging and tracking.EEM analysis of endocytosed HRP‐tagged BoNT/Ai. Representative images show cytochemically stained HRP precipitate (arrowheads). Endo/lysosomes, multivesicular bodies (MVB), structures with the morphology of autophagosomes (AP), and (i) presynaptic (Ps) and (ii) axonal (Ax) plasma membrane (PM). Postsynaptic density (asterisks), nonstained endocytic compartments (arrows), and background binding on the PM (arrows in ii) are indicated. Scale bars 100 nm.F, G(F) Percentage of total BoNT/Ai^wt^‐HRP and indicated mutants localized on the PM or in the indicated endocytic structures, and (G) the average sectional area of the HRP‐stained endocytic compartments. Data information: Error bars are shown as standard error of the mean (± SEM). (B–D) *N* = 17 technical replicates/condition from five to six independent biological replicates, and (F, G) *n* = 60 electron micrographs/condition from three independent biological replicates. Statistical test using nonparametric Kruskal–Wallis multiple comparison test. *P < 0.05, **P < 0.01, ***P < 0.001, ****P < 0.0001, ns—nonsignificant.

To study BoNT/Ai^wt^‐At647N binding to the plasma membrane of cultured rat hippocampal neurons, we performed universal point accumulation imaging in nanoscale topography (uPAINT) super‐resolution imaging (Giannone *et al*, 2013; Appendix Fig S3A). Cultured mature (days *in vitro* DIV21‐22) hippocampal neurons were stimulated with high K^+^ buffer in the presence of 100 pM BoNT/Ai^wt^‐At647N, BoNT/Ai^PSG^‐At647N, BoNT/Ai^SV2^‐At647N, or BoNT/Ai^PSG,SV2^‐At647N and immediately imaged using total internal reflection fluorescence (TIRF) microscopy. To track the internalized toxin molecules, we used a pulse‐chase super‐resolution imaging technique called subdiffractional tracking of internalized molecules (sdTIM; Joensuu *et al*, 2016, 2017; Appendix Fig S3A; Movie EV2). For this, cultured mature (DIV21‐22) hippocampal neurons were stimulated with high K^+^ buffer for 5 min in the presence of 1 nM BoNT/Ai^wt^‐At647N, BoNT/Ai^PSG^‐At647N, BoNT/Ai^SV2^‐At647N, or BoNT/Ai^PSG,SV2^‐At647N at 37°C, after which the neurons were washed with low K^+^ buffer to remove any unbound toxins, chased for 10 min at 37°C to allow endocytic uptake of the toxins and then imaged live using highly inclined and laminated optical sheet (HILO) microscopy. Single‐molecule tracking of the toxins allowed us to conduct nanoscale analysis of the dynamic changes in their mobility upon initial binding to the plasma membrane, and following endocytosis (representative super‐resolved average intensity maps, average diffusion coefficient maps, and single‐molecule tracks of BoNT/Ai^wt^‐At647N on the plasma membrane and following endocytosis are shown in Fig 1A). Comparison of the number of holotoxins that bound to the plasma membrane demonstrated that abolishment of PSG binding (BoNT/Ai^PSG^‐At647N and BoNT/Ai^PSG,SV2^‐At647N) significantly decreased the binding of the toxin compared with BoNT/Ai^wt^‐At647N, whereas abolishment of SV2 binding (BoNT/Ai^SV2^‐At647N) did not (Fig 1B, uPAINT). This indicates that although the initial binding of the toxin to the plasma membrane is primarily PSG‐dependent, BoNT/A can bind to SV2 without prior PSG binding, albeit to a lesser extent. A similar decrease in the number of BoNT/Ai^PSG^‐At647N and BoNT/Ai^PSG,SV2^‐At647N, but not BoNT/Ai^SV2^‐At647N, molecules were also observed following activity‐dependent endocytic uptake of the toxins compared with BoNT/Ai^wt^‐At647N (Fig 1C, sdTIM). Comparison of the uPAINT and sdTIM imaging data revealed a significant decrease in single‐molecule mobility following internalization of BoNT/Ai^wt^‐At647N (Fig 1D; Appendix Fig S3B and C), reflecting endocytic uptake of the toxins into SVs (Joensuu *et al*, 2016). A similar decrease in mobility was not observed for BoNT/Ai^PSG^‐At647N, BoNT/Ai^SV2^‐At647N, or BoNT/Ai^PSG,SV2^‐At647N (Fig 1D; Appendix Fig S3D–I), suggesting that endocytosis of all the receptor‐binding mutant toxins was perturbed.

To investigate whether the endocytosis of the receptor‐binding mutant toxins was perturbed, we next conducted pulse‐chase experiments of horseradish peroxidase (HRP)‐labeled BoNT/Ai^wt^, BoNT/Ai^PSG^, BoNT/Ai^SV2^, and BoNT/Ai^PSG,SV2^. For this, cultured mature (DIV21‐22) hippocampal neurons were stimulated with high K^+^ buffer in the presence of 33 nM BoNT/Ai^wt^‐HRP and the receptor‐binding mutants at 37°C for 5 min, after which the neurons were washed with low K^+^ to remove unbound toxins, chased for 10 min to allow endocytic uptake of the toxins, then fixed, cytochemically stained and processed for EM. The great majority of the BoNT/Ai^wt^‐HRP was internalized into SVs, with only a small portion either localizing into other endocytic compartments (endo‐lysosomes, multivesicular bodies [MVBs] and structures with the morphology of autophagosomes [APs]) or remaining on the plasma membrane (Fig 1E and F). By contrast, most of the receptor‐binding mutant toxins were found on the plasma membrane (representative EM images are shown in Fig 1Ei and ii). A small fraction of internalized mutant toxin was found in APs, demonstrating a defect in the endocytic sorting of the toxin mutants into SVs, with mistargeting into a degradative pathway (Fig 1F). Reflecting the entry of the mutant holotoxins into endocytic structures other than SVs, the average sectional areas of the HRP‐stained endocytic compartments containing receptor‐binding mutant toxins were significantly larger than those obtained with BoNT/Ai^wt^‐HRP, which was primarily observed in small SVs (average diameter 0.04 μm; Fig 1G).

Polysialogangliosides are considered to be the initial target of BoNT/A due to their abundance on the presynaptic membrane (Simpson & Rapport, 1971a,b; Schwarzmann & Sandhoff, 1990; Yowler & Schengrund, 2004; Stenmark *et al*, 2008; Hamark *et al*, 2017; Schnaar *et al*, 2022). Our single‐molecule imaging results demonstrated that BoNT/A with an inactivated PSG‐binding site was able to bind SV2 at pM toxin concentrations without prior PSG binding. However, based on our EM analysis, BoNT/A binding to SV2 alone was insufficient for endocytic uptake of the toxin into SVs, stranding the toxin on the plasma membrane or mistargeting it upon entry into a degradative endocytic pathway. These results indicate that BoNT/A must coincidentally detect both PSG and SV2 to selectively target SVs upon endocytosis.

### BoNT/A‐induced SV2A immobilization on the neuronal plasma requires Syt1

**Figure 2:**
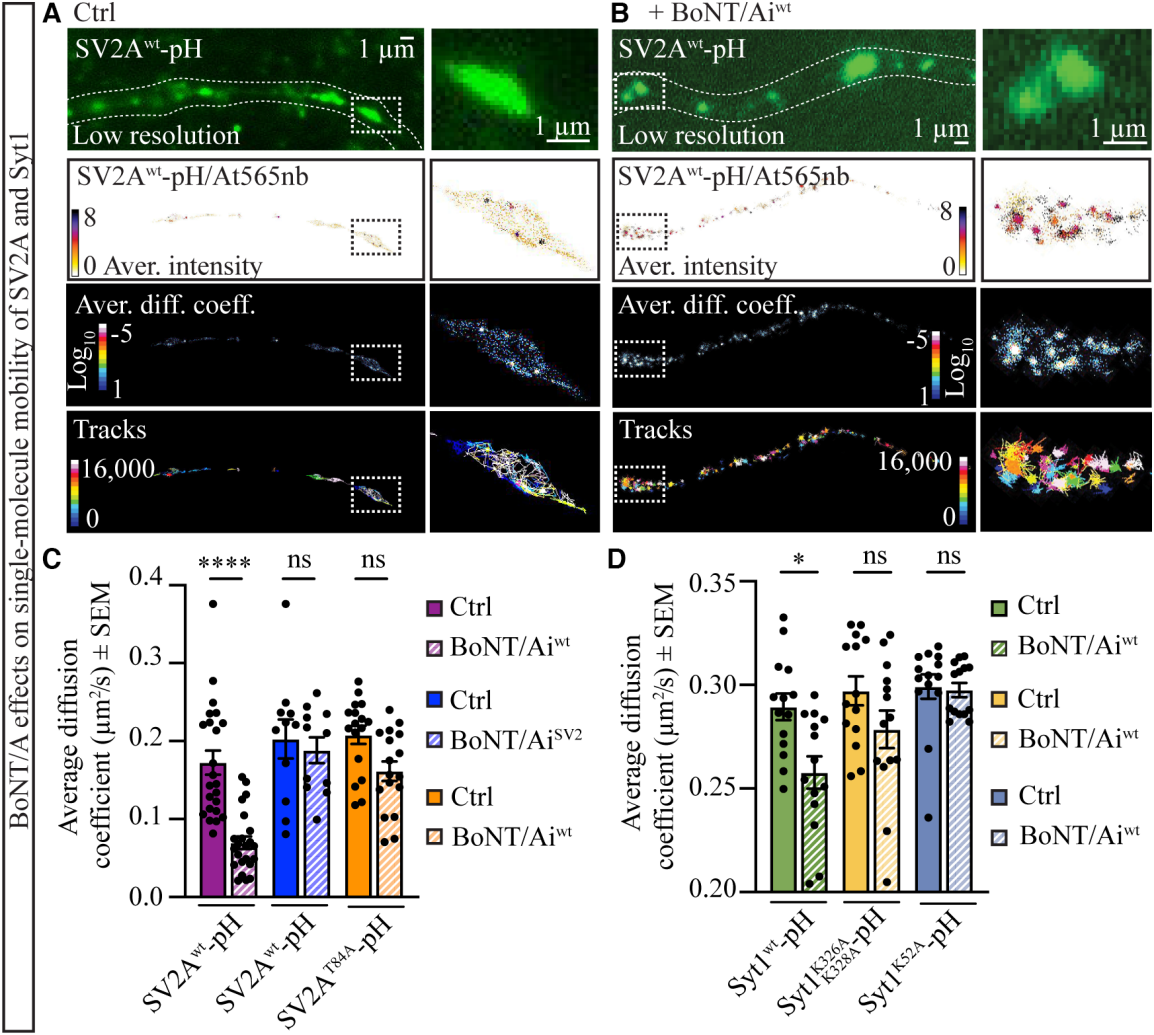
BoNT/A‐induced immobilization of SV2 and Syt1 on the neuronal plasma membrane depends on Syt1‐SV2 and PSG‐Syt1 interactions A, BLow‐resolution images and the corresponding super‐resolved uPAINT average intensity (bar: high 8 to low 0 density), average diffusion coefficient (bar: Log_10_D high 1 to low −5 mobility) and trajectory maps of SV2A^wt^‐pH/At565nb in (A) control conditions (high K^+^; Ctrl), and (B) following treatment with 100 pM BoNT/Ai^wt^‐At647N (supplemented in high K^+^).CScatter plots of the average diffusion coefficient (μm^2^ s^−1^) single‐molecule mobility of SV2A^wt^‐pH/At565nb and SV2A^T84A^‐pH/At565nb in the control condition (high K^+^; Ctrl) and following treatment with 100 pM BoNT/Ai^wt^‐At647N or BoNT/Ai^SV2^‐At647N (supplemented in high K^+^).DScatter plots of the average diffusion coefficient (μm^2^ s^−1^) single‐molecule mobility of Syt1^wt^‐pH/At565nb, Syt1^K326A,K328A^‐pH/At565nb and Syt1^K52A^‐pH/At565nb in the control condition (high K^+^; Ctrl) and following treatment with 100 pM BoNT/Ai^wt^‐At647N (supplemented in high K^+^). Data information: Error bars are shown as standard error of the mean (± SEM). *N* = 11–23 technical replicates/condition from three to five independent biological replicates. Statistical test using nonparametric Kruskal–Wallis multiple comparison test. *P < 0.05, ****P < 0.0001, and ns—nonsignificant.

What then is the role of the coincidental binding of BoNT/A to PSGs and SV2? To answer this question, we first investigated how the initial binding of BoNT/A to SV2 affects SV2 nanoscale organization by performing uPAINT imaging. For this, hippocampal neurons transiently expressing pHluorin‐tagged SV2A^wt^ (SV2A^wt^‐pH) (Zhang *et al*, 2015) were stimulated with high K^+^ buffer supplemented with 100 pM anti‐GFP Atto565 nanobodies (At565nb) in the presence or absence of 100 pM BoNT/Ai^wt^, and the mobility of the receptor was immediately imaged with TIRF microscopy (Fig 2A and B; Movie EV3). Anti‐GFP At565nb binds to the luminal pH sensitive pHluorin‐tag inserted into luminal domain 1 of SV2A (between SV2A amino acids 197 and 198), which is exposed to the extracellular environment following stimulated SV exocytosis, allowing single‐molecule tracking of the receptor on the neuronal plasma membrane (Fig 2A and B). Quantification of SV2A^wt^‐pH/At565nb mobility demonstrated that BoNT/Ai^wt^ binding to the neuronal plasma membrane immobilized SV2A to distinct areas of confinement (Fig 2A and B), significantly decreasing the single‐molecule mobility of the receptor (Fig 2C; Appendix Fig S4A–D), whereas no such effect was observed following BoNT/Ai^SV2^ treatment (Fig 2C; Appendix Fig S4E–J). These results demonstrate that BoNT/A binding to its protein receptor restricts the lateral diffusion of SV2A on the neuronal plasma membrane prior to the endocytosis of the toxin into SVs.

We next focused on how BoNT/A immobilizes SV2A. All SV2 isoforms (A–C) have been shown to bind Syt1 through their cytoplasmic domains (Schivell *et al*, 2005), forming so‐called intrinsic trafficking partners (iTRAPs) (Gordon & Cousin, 2016; Bartholome *et al*, 2017), which ensure correct clustering and efficient retrieval of Syt1 and SV2 from the neuronal plasma membrane into recycling SVs (Lazzell *et al*, 2004; Yao *et al*, 2010; Kaempf *et al*, 2015; Zhang *et al*, 2015; Harper *et al*, 2020). In agreement with this, we recently demonstrated that SV2A‐Syt1 nanoclusters control the selective retrieval of these two proteins from the plasma membrane into SVs (Small *et al*, 2021a). The direct Syt1‐SV2 interaction occurs through the C2B domain (K326/K328) (Zhang *et al*, 2015) of Syt1 and the N‐terminal threonine 84 (T84) of SV2 (Schivell *et al*, 1996, 2005), and is strongly potentiated by phosphorylation of T84 by casein kinase 1 family kinases (Pyle *et al*, 2000; Zhang *et al*, 2015). We therefore hypothesized that the observed BoNT/A‐induced immobilization of SV2A prior to endocytosis was dependent on Syt1‐SV2 interactions. To test this, we repeated the uPAINT experiments using the SV2A mutant with abolished binding to Syt1 (SV2A^T84A^‐pH). Although a small decrease in the single‐molecule mobility of SV2A^T84A^‐pH/At565nb was observed following BoNT/Ai^wt^ treatment, the decrease was not significant (Fig 2C; Appendix Fig S4K–P), indicating that the toxin‐induced SV2A immobilization on the neuronal plasma membrane was indeed dependent on its ability to interact with Syt1.

To investigate the possible involvement of Syt1 in BoNT/A intoxication, we next studied the effects of BoNT/Ai^wt^ on the uPAINT mobility of Syt1^wt^‐pH (Diril *et al*, 2006; Harper *et al*, 2020). Our results revealed that, similar to SV2A^wt^‐pH/At565nb, Syt1^wt^‐pH/At565nb became more immobile following toxin exposure (Fig 2D; Appendix Fig S5A–E). This result was surprising, given that Syt1 does not directly bind to BoNT/A (Dong *et al*, 2003; Rummel *et al*, 2004a), suggesting that the effect of the toxin on Syt1 mobility is likely to be indirect. We then repeated the uPAINT experiments with the Syt1 K326A/K328A mutant, which abolishes binding to SV2 (Zhang *et al*, 2015), and observed a markedly reduced effect of BoNT/Ai^wt^ on Syt1^K326A,K328A^‐pH/At565nb mobility (Fig 2D; Appendix Fig S5F–J). Together, these results indicate that BoNT/A immobilizes SV2A and Syt1 receptors on the neuronal plasma membrane, dependent on Syt1‐SV2 interaction.

We next studied the role of BoNT/A binding to PSG. A recent study reported that BoNT/B targets a preassembled GT1b‐Syt1 complex on the outer leaflet of the plasma membrane to intoxicate neurons (Flores *et al*, 2019; Ramirez‐Franco *et al*, 2021). The authors demonstrated that a lysine (K) 52 to alanine (A) (K52A) mutation in the N‐terminal glycosphingolipid‐binding motif of Syt1 disrupted the GT1b‐Syt1 interaction, leading to deficient binding and entry of BoNT/B in neuroendocrine cells (Flores *et al*, 2019). Given that BoNT/B shares a conserved PSG‐binding site with BoNT/A (Rummel, 2013), we hypothesized that a similarly preassembled glycolipid‐protein complex (PSG‐Syt1) may also be targeted by BoNT/A, with the PSG serving as a molecular bridge between the toxin and Syt1. To investigate this possibility, we created a Syt1^K52A^‐pH construct that disrupts the formation of the PSG‐Syt1 complex and performed uPAINT imaging of Syt1^K52A^‐pH/At565nb with and without BoNT/Ai^wt^ treatment. The immobilization effect of Syt1 following toxin treatment was completely abolished following the disruption of the PSG‐Syt1 complex (Fig 2D; Appendix Fig S5K–O). Together, our single‐molecule imaging results indicate that BoNT/A promotes the confinement of SV2A and Syt1 on the neuronal plasma membrane and that this depends on their ability to interact with each other. Our results further suggest that Syt1 is engaged in the BoNT/A intoxication process through the PSG‐Syt1 complex.

### BoNT/A enhances SV2A‐Syt1 interactions and their nanoclustering on the neuronal plasma membrane

**Figure 3:**
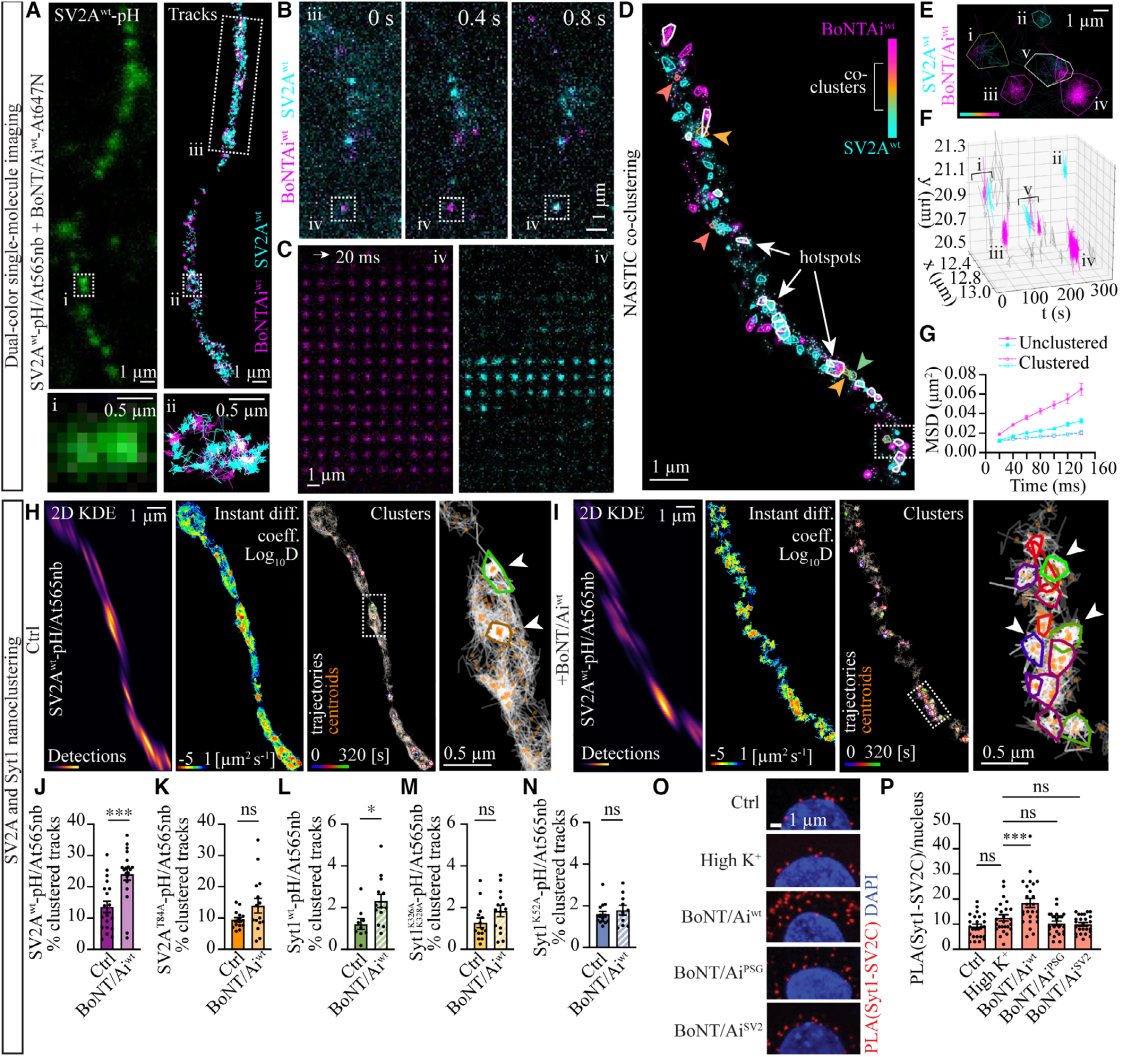
BoNT/A induces Syt1‐SV2 interactions and receptor nanoclustering A–C(A) Representative low‐resolution image of SV2A^wt^‐pH (green), and corresponding dual‐color super‐resolved uPAINT trajectories of SV2A^wt^‐pH/At565nb (cyan) imaged simultaneously with BoNT/Ai^wt^‐At647N (magenta). Regions (i–ii) are magnified below, and (iii) is magnified in (B) showing a representative time‐lapse sequence of the single molecules of SV2A^wt^‐pH/At565nb (cyan) and BoNT/Ai^wt^‐At647N (magenta) detected coincidentally in a neuronal projection. The 10 × 10 pixel boxed region (iv) is magnified in (C), showing frame‐by‐frame coincidental detection of BoNT/Ai^wt^‐At647N and SV2A^wt^‐pH/At565nb obtained using a 50 Hz (20 ms exposure time).D, E(D) Representative NASTIC nanocluster analysis image of BoNT/Ai^wt^‐At647N and SV2A^wt^‐pH/At565nb showing outlined nanoclusters in magenta and cyan, respectively. BoNT/Ai^wt^‐At647N and SV2A^wt^‐pH/At565nb co‐clusters are indicated by colored arrowheads and hotspots with repeated nanoclustering events by white outlines. Boxed area in the bottom is shown magnified in (E), which shows examples of (i) a BoNT/Ai^wt^‐At647N and SV2A^wt^‐pH/At565nb co‐cluster, (ii) an SV2A^wt^‐pH/At565nb cluster, (iii, iv) two BoNT/Ai^wt^‐At647N clusters, and a hotspot (v) as indicated.F3D representation of the 2D image in (E), with corresponding i–v clusters.GMSD graph of unclustered and clustered BoNT/Ai^wt^‐At647N (magenta) and SV2A^wt^‐pH/At565nb (cyan).H, IRepresentative images of NASTIC nanocluster analysis of SV2A^wt^‐pH/At565nb in (H) the control condition (high K^+^; Ctrl) and (I) following high K^+^ stimulation in the presence of 100 pM BoNT/Ai^wt^‐At647N, with 2D kernel density estimation (KDE, high density in red), instantaneous Log_10_ diffusion coefficient map (low mobility in red), and nanoclusters with their respective trajectories (white) and centroids (orange) shown. Boxed areas are magnified on the right, with arrowheads indicating nanoclusters.J–NQuantification of the (J) SV2A^wt^‐pH/At565nb, (K) SV2A^T84A^‐pH/At565nb, (L) Syt1^wt^‐pH/At565nb, (M) Syt1^K326A,K328A^‐pH/At565nb and (N) Syt1^K52A^‐pH/At565nb nanoclustering in control conditions (high K^+^; Ctrl) and following BoNT/Ai^wt^‐At647N treatment (diluted in high K^+^).O
*In situ* proximity ligation assay (PLA) of Syt1‐SV2C interactions in naïve (ctrl), high K^+^ stimulated (vehicle) conditions, and following high K^+^ stimulation with the indicated toxins. Nucleus (DAPI; blue) and Syt1‐SV2C PLA (red) signals from a representative confocal maximum intensity projection of the somatodendritic area.PNumber of PLA (Syt1‐SV2C) dots per nucleus in the indicated conditions. Data information: Error bars are shown as standard error of the mean (± SEM). (J–N) *N* = 11–19 technical replicates from three to five independent biological replicates, and (P) *n* = 25 regions of interest (200 × 200 pixel ROI surrounding DAPI staining)/condition from two independent biological replicates. Unpaired *t* test in (J–N) and nonparametric Kruskal–Wallis multiple comparison test in (P). *P < 0.05, ***P < 0.001, ns—nonsignificant.

The binding of BoNT/A to PSGs has been suggested to concentrate the toxin on the neuronal plasma membrane and to laterally move it to its protein receptor (Montecucco, 1986). To investigate whether BoNT/A moves on the plasma membrane to the sites enriched with surface SV2, we performed dual‐color single‐molecule uPAINT imaging of the toxin and SV2A. For this, hippocampal neurons transiently expressing SV2A^wt^‐pH were stimulated with high K^+^ buffer supplemented with 100 pM At565nb and 100 pM BoNT/Ai^wt^‐At647N, and the mobility of the receptor and the toxin were simultaneously imaged with TIRF microscopy (Fig 3A; Movie EV3). Dual‐color single‐molecule tracking revealed immobilized SV2A and toxin in the same diffraction‐limited areas on the neuronal plasma membrane (Fig 3B and C). Using nanoscale spatiotemporal indexing clustering (NASTIC) analysis (Wallis *et al*, 2021), which allowed us to investigate the nanoclustering of BoNT/A and SV2A on the plasma membrane in live neurons, we discovered that the immobilized toxin and SV2A molecules were observed in the same diffraction‐limited areas, forming occasional co‐clusters (Fig 3D–F). Prior to co‐clustering, both the toxin and SV2A were observed to diffuse on the plasma membrane with distinct mobilities, BoNT/A being more mobile than its receptor when not engaged in nanoclusters (Fig 3G). Following their lateral trapping in nanoclusters, sometimes repeatedly in the same diffraction‐limited areas (i.e., hotspots in Fig 3D–F), the mobility of both BoNT/A and SV2A decreased substantially and became indistinguishable (Fig 3G). These results demonstrate that SV2 is a dynamic rather than static target of the toxin and that SV2A and BoNT/A form nanoclusters on the neuronal plasma membrane prior to endocytosis into SVs.

Next, we studied the spatiotemporal nanoclustering of SV2A and Syt1 on the plasma membrane using NASTIC cluster analysis of our uPAINT data. As anticipated, we observed a significant increase in the nanoclustering of SV2A^wt^‐pH/At565nb (Fig 3H–J), but not of SV2A^T84A^‐pH/At565nb (Fig 3K), following BoNT/Ai^wt^ exposure, indicating that the BoNT/A‐induced changes in SV2A nanoclustering depend on Syt1‐SV2A interactions. In agreement with this, we also observed a small but significant increase in Syt1^wt^‐pH/At565nb nanoclustering following BoNT/Ai^wt^ treatment (Fig 3L; Appendix Fig S6A and B). A similar increase in clustering was not observed for Syt1^K326A,K328A^‐pH/At565nb (Fig 3M) or Syt1^K52A^‐pH/At565nb (Fig 3N).

We then investigated the effects of BoNT/A on Syt1 and SV2 interactions on the plasma membrane using the *in situ* proximity ligation assay (PLA). Following immunolabeling of endogenous Syt1 and SV2C, the PLA produced productive ligation as evidenced by the detection of discrete fluorescent dots (Fig 3O). This suggested that Syt1 and SV2C interact at these locations, as the ligation step requires that the two proteins of interest are in close proximity to each other (< 40 nm). Our results showed that the number of Syt1‐SV2C PLA dots in the synapse‐rich somatodendritic area of the neurons increased significantly following BoNT/Ai^wt^‐At647N exposure compared with naïve (nontreated control) or high K^+^‐stimulated (vehicle control) neurons or following exposure to BoNT/Ai^PSG^‐At647N or BoNT/Ai^SV2^‐At647N (Fig 3P). Together with our previous findings, these results indicate that BoNT/A promotes Syt1‐SV2C interactions on the neuronal plasma membrane when the toxin coincidentally engages with both PSG and SV2, but not when it is bound to either of the receptors alone. Based on our nanoclustering results, the approximately two‐fold increase in SV2A and Syt1 nanoclustering caused by the toxin was dependent on both SV2A‐Syt1 and PSG‐Syt1 interactions, suggesting that the coincidental binding of BoNT/A to PSG and SV2 has two purposes: binding to PSG functions as a molecular bridge that engages Syt1 in BoNT/A intoxications, and coincidental binding of the PSG‐Syt1‐bound BoNT/A to SV2A increases SV2A‐Syt1 interactions via the cytoplasmic domains of the two transmembrane proteins.

### Syt1 KD leads to loss of BoNT/A toxic function

**Figure 4:**
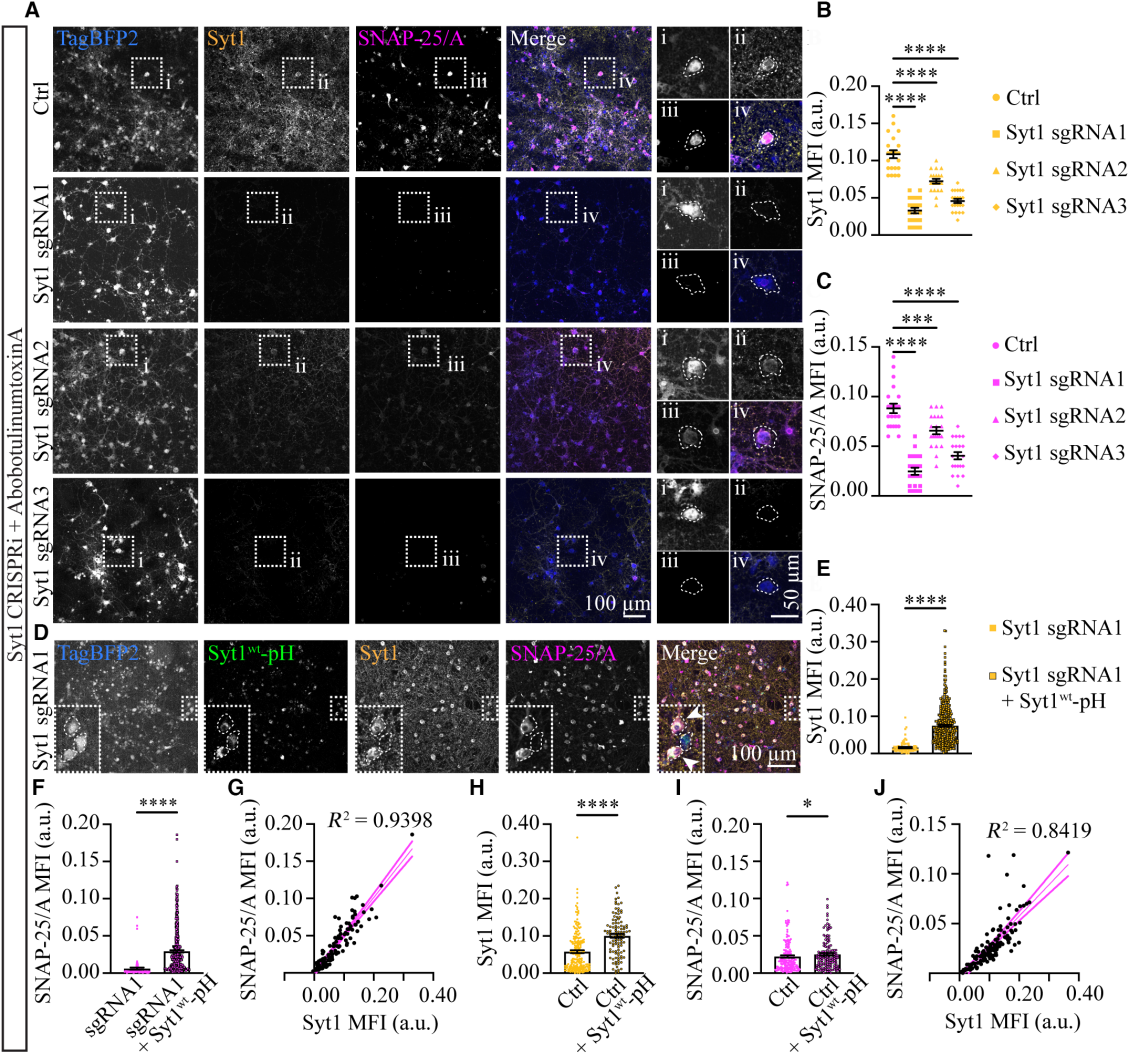
Syt1 KD leads to loss of BoNT/A‐induced SNAP‐25 cleavage ARepresentative confocal Z‐stack sum projections of neurons transduced with TagBFP2‐expressing (blue) control (ctrl; nontargeting sgRNA) and CRISPRi Syt1 KD (Syt1 sgRNA1‐3) lentiviruses. Neurons were treated for 30 min with 10 units of AbobotulinumtoxinA, and immunostained for endogenous Syt1 (yellow) and BoNT/A‐cleaved SNAP‐25 (SNAP‐25/A; magenta). Boxed regions (i‐iv) are magnified on the right, with neuronal somas outlined by dashed lines for clarity.B, CQuantification of mean fluorescence intensity (MFI) of endogenous (B) Syt1, and (C) cleaved SNAP‐25 (SNAP‐25/A), in control and Syt1 sgRNA1‐3 KD neurons following AbobotulinumtoxinA treatment.DA representative confocal Z‐stack sum projection of neurons transduced with TagBFP2‐expressing (blue) CRISPRi Syt1 KD (sgRNA1) lentiviruses and rescued with pLenti6.3‐Syt1^wt^‐pH (green). Neurons were treated with AbobotulinumtoxinA (10 units) for 30 min, and immunostained for endogenous Syt1 (yellow) and SNAP‐25/A (magenta). Boxed regions are magnified in the insets, with rescued neurons indicated by arrowheads and neuronal somas outlined by dashed lines for clarity.EQuantification of the MFI of endogenous Syt1 in Syt1 sgRNA1 KD neurons with and without pLenti6.3‐Syt1^wt^‐pH rescue. Neurons were treated with AbobotulinumtoxinA (10 units) for 30 min.FQuantification of the MFI of endogenous cleaved SNAP‐25 (SNAP‐25/A) in Syt1 sgRNA1 KD neurons with and without pLenti6.3‐Syt1^wt^‐pH rescue. Neurons were treated with AbobotulinumtoxinA (10 units) for 30 min.GLinear regression graph showing Syt1 and cleaved SNAP‐25/A MFI quantification from a representative confocal Z‐stack sum projection of hippocampal neurons transduced with CRISPRi Syt1 sgRNA1 lentiviruses. Neurons were treated for 30 min with 10 units of AbobotulinumtoxinA, and immunostained for endogenous Syt1 (yellow) and BoNT/A‐cleaved SNAP‐25 (SNAP25/A). Solid bold lines indicate 95% confidence intervals, and R^2^ represents the proportion of the variance.H, IQuantification of (H) Syt1 and (I) SNAP‐25/A MFI in nontransduced neurons (neurons negative for TagBFP2) with and without pLenti6.3‐Syt1^wt^‐pH expression following AbobotulinumtoxinA treatment (10 units, 30 min.)JLinear regression graph showing Syt1 and cleaved SNAP‐25/A MFI quantification from a representative confocal Z‐stack sum projection of hippocampal neurons transduced with pLenti6.3‐Syt1^wt^‐pH. Neurons were treated for 30 min with 10 units of AbobotulinumtoxinA, and immunostained for Syt1 (yellow) recognizing both the endogenous Syt1 and Syt1^wt^‐pH, and BoNT/A‐cleaved SNAP‐25 (SNAP‐25/A). Solid bold lines indicate 95% confidence intervals, and R^2^ represents the proportion of the variance. Data information: Error bars are shown as standard error of the mean (± SEM). (B, C) *n* = 21 technical replicates/condition from two independent biological replicates. (E, F, H, I) *n* = 3 technical replicates/condition from one biological experiment. Statistical significance was assessed using a nonparametric Kruskal–Wallis multiple comparison test (B, C) and a nonparametric Mann–Whitney *U* test (E, F, H, I). *P < 0.05, ***P < 0.001, ****P < 0.0001 and ns—nonsignificant.

To determine whether Syt1 has a functional role in BoNT/A intoxication, we next investigated the effects of Syt1 KD on BoNT/A‐induced cleavage of synaptosomal‐associated protein 25 kDa (SNAP‐25), a component of the SNARE exocytic machinery, which is a hallmark of BoNT/A‐induced neurointoxication (Dong *et al*, 2019). Given that Syt1 is a long‐lived protein that is challenging to deplete (Vevea & Chapman, 2020), we employed a lentiviral clustered regularly interspaced short palindromic repeats interference (CRISPRi) tool using 3 short guide RNAs (sgRNAs; sgRNA1‐3) to achieve Syt1 KD in cultured mature hippocampal neurons (KD for 7 days). Following a 30 min exposure to 10 units of AbobotulinumtoxinA (a BoNT/A‐based licensed drug), DIV21‐22 neurons were fixed and processed for immunofluorescence staining using antibodies recognizing endogenous Syt1 and cleaved SNAP‐25/A (the SNAP‐25/A antibody specifically recognizes amino acids 189–197, a sequence which is only exposed upon BoNT/A‐mediated cleavage of SNAP‐25), after which they were imaged with spinning‐disk confocal microscopy. We introduced Tag Blue Fluorescent Protein 2 (TagBFP2) in the lentivirus vector expression cassette under a separate promoter to identify lentiviral‐transduced neurons. TagBFP2 fluorescence was used for the automatic segmentation of neuronal regions of interest (ROIs), from which Syt1 and SNAP‐25/A mean fluorescence intensity (MFI) levels were quantified (Appendix Fig S7A). Representative maximum intensity Z‐projections of the acquired confocal stacks (Fig 4A), and the MFI quantification of Syt1 and SNAP‐25/A immunostaining in CRISPRi control and Syt1 sgRNA1‐3‐induced neurons (Fig 4B and C, respectively), demonstrated that the most efficient KD of Syt1 was achieved with sgRNA1 (on average, 69.7% KD efficiency) compared with sgRNA2 (33.3% KD efficiency) and sgRNA3 (57.9% KD efficiency), and that the level of SNAP‐25 cleavage correlated with the efficiency of Syt1 KD (71.9, 25.4 and 54.1% decrease in SNAP‐25 cleavage, respectively). Interestingly, we also observed that Syt1 sgRNA1 KD was protective over BoNT/E‐induced cleavage of SNAP‐25 (Appendix Fig S7B–D), These results suggest that Syt1 may also have a role in the intoxication process of other BoNT serotypes that target PSGs and SV2 as their receptors.

These results were confirmed using short hairpin RNA (shRNA) against Syt1 in cultured mature hippocampal neurons. For this, DIV7 hippocampal neurons were co‐transfected with pEGFP and Syt1 targeting shRNA or nontargeting control plasmid and cultured for 14 days. Following a 30 min or 16 h exposure to 10 units of AbobotulinumtoxinA on DIV21‐22, the neurons were fixed and processed for immunofluorescence as described above. The MFIs of endogenous Syt1 and cleaved SNAP‐25 following 30 min exposure to AbobotulinumtoxinA were quantified from neurons positive for EGFP (Appendix Fig S7E), demonstrating that the level of Syt1 shRNA KD correlated with SNAP‐25 cleavage (on average, 54.4% Syt1 shRNA KD led to a 39.5% decrease in SNAP‐25 cleavage; Appendix Fig S7F–H). Prolonged exposure (16 h) to AbobotulinumtoxinA slightly increased the levels of SNAP‐25 cleavage (Appendix Fig S7I–K), indicating either a delayed intoxication effect and/or that the toxin may enter the neurons through an alternative endocytic pathway following prolonged exposure. Lentivirally induced Syt1^wt^‐pH re‐expression (pLenti6.3‐Syt1^wt^‐pH) in the Syt1 sgRNA1 KD background rescued the CRISPRi phenotype (Fig 4D–G), whereas pLenti6.3‐Syt1^wt^‐pH overexpression (Fig 4H; on average, 73.4% increase compared with endogenous Syt1) in wildtype hippocampal neurons led to a significant increase in SNAP‐25 cleavage following toxin treatment (Fig 4I and J), indicating a gain of toxic function and suggesting that the expression level of Syt1 is rate limiting for BoNT/A intoxication. By contrast, pLenti6.3‐Syt1^K52A^‐pH expression was less effective in rescuing the SNAP‐25 cleavage phenotype than pLenti6.3‐Syt1^wt^‐pH (Appendix Fig S7L–N; on average, SNAP‐25 cleavage increased > 4‐fold and 1.5‐fold when rescued with pLenti6.3‐Syt1^wt^‐pH and pLenti6.3‐Syt1^K52A^‐pH, respectively). These results demonstrate that Syt1 KD is protective against BoNT/A intoxication.

Knockdown or deletion of SV2A/B can lead to missorting or reduced expression of Syt1 (Kaempf *et al*, 2015). To ensure that the KD of Syt1 did not significantly affect the expression level or localization of SV2A, we performed qPCR (Appendix Fig S8A), immunofluorescence quantification of endogenous levels of Syt1 and SV2A (Appendix Fig S8B–D), and immunofluorescence co‐localization analysis of SV2A with the plasma membrane marker Ezrin (Appendix Fig S8E–G) following a 7‐day Syt1 CRISPRi sgRNA KD in primary hippocampal neurons. Our results revealed no significant changes in SV2A mRNA levels, SV2A protein levels, or SV2A plasma membrane localization, respectively. We also investigated the binding efficacy of BoNT/Ai^PSG^‐At647N (only binds to SV2) on control and Syt1 sgRNA1 KD neuronal plasma membrane in an immunofluorescence analysis, and observed no changes in the levels of the toxin that bound to the membrane (Appendix Fig S8H–J), verifying that the 7‐day Syt1 sgRNA1 KD did not affect significantly the neuronal expression or localization of SV2A.

**Figure 5:**
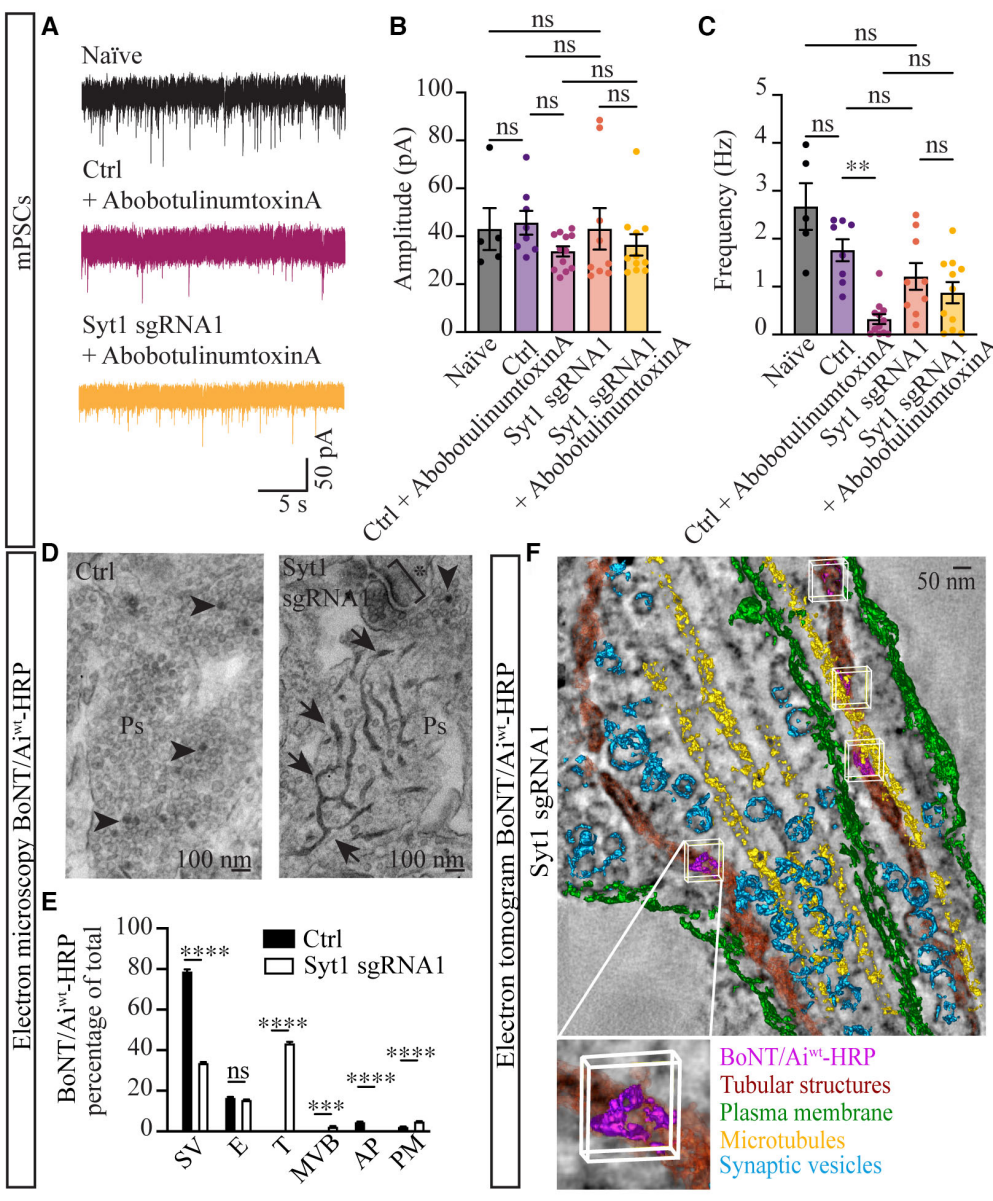
Syt1 KD is protective against BoNT/A neurointoxication by mislocalizing the toxin upon entry into neurons ARepresentative patch‐clamp spontaneous miniature inhibitory postsynaptic current (mPSC) recordings in naïve neurons, and in control (nontargeting) and Syt1 KD (CRISPRi sgRNA1) neurons following a 30 min treatment with AbobotulinumtoxinA (10 units).B, CQuantification of mPSC (B) peak amplitudes and (C) frequencies for indicated conditions.DEM analysis of endocytosed BoNT/Ai^wt^‐HRP following lentiviral control (ctrl; nontargeting sgRNA) and Syt1 sgRNA1 CRISPRi KD transduction. HRP precipitate in synaptic vesicles (SVs; arrowheads) and tubular structures (arrows) in presynapses (Ps) are indicated. The postsynaptic density is marked with an asterisk.EPercentage of total BoNT/Ai^wt^‐HRP localized in SVs, endo/lysosomes (E), tubular structures (T), multivesicular bodies (MVB), structures resembling autophagosomes (AP) and on the plasma membrane (PM) in the indicated conditions.FElectron tomogram of endocytosed BoNT/Ai^wt^‐HRP (purple with white bounding boxes) in tubular structures (red) induced by Syt1 sgRNA1 KD. PM (green), SVs (cyan), microtubules (yellow). Data information: Error bars are shown as standard error of the mean (± SEM). *N* = 5–12 technical replicates/condition from two independent biological replicates in (B, C), *n* = 56 regions of interest/ condition from two independent biological replicates (E), statistical significance was assessed using a nonparametric Kruskal–Wallis multiple comparison test (B), an ordinary one‐way ANOVA multiple comparison test (C) and two‐way ANOVA multiple comparison test (E). **P < 0.01, ***P < 0.001, ****P < 0.0001, and ns—nonsignificant.

To verify the protective effect of Syt1 KD against BoNT/A intoxication, we next performed whole‐cell patch‐clamp recordings of spontaneous miniature postsynaptic currents (mPSCs) in DIV21‐22 hippocampal neurons at a holding potential of −70 mV in the presence of 1 nM tetrodotoxin (bath‐applied) to block action potentials. Representative traces from a naïve hippocampal neuron (untreated), nontargeting sgRNA control neuron (treated with 10 units of AbobotulinumtoxinA for 30 min), and CRISPRi Syt1 sgRNA1‐treated neuron (also treated with AbobotulinumtoxinA for 30 min) are shown in Fig 5A. Our recordings revealed that the mPSC peak amplitudes were similar when averaged over the cells across all conditions (Fig 5B), supporting earlier findings that Syt1 depletion (Bacaj *et al*, 2013) or BoNT/A2 subtype treatment (Akaike *et al*, 2010) do not significantly change mPSC amplitude. In further support of previous findings (Beske *et al*, 2015), we observed a significant decrease in mPSC frequency in control neurons following AbobotulinumtoxinA treatment (Fig 5C). We also observed a decrease in mPSC frequency in Syt1 sgRNA1 KD neurons compared with naïve (untreated) neurons (Fig 5C). Importantly, AbobotulinumtoxinA treatment in Syt1 sgRNA1 KD neurons did not promote a further reduction in the mPSC frequency and was protective over the mPSC frequency reduction observed in control neurons (Fig 5C), supporting our finding that Syt1 KD has a protective effect against BoNT/A intoxication. Together, these results demonstrate that reducing Syt1 expression levels leads to the loss of BoNT/A‐induced cleavage of SNAP‐25 and prevents the blockage of presynaptic neurotransmission.

### Syt1 KD blocks the targeted entry of BoNT/Ai into synaptic vesicles

Syt1 KD could be envisioned to prevent BoNT/A intoxication via two alternative mechanisms, either by preventing the endocytic uptake of BoNT/A (i.e., the toxin remains stranded on the plasma membrane) or by mistargeting it to nonacidic endocytic compartments upon endocytosis. In the latter scenario, BoNT/A would be unable to undergo the conformational change required for the translocation of BoNT/A‐LC, and subsequent cleavage of SNAP‐25 (Blasi *et al*, 1993; Schiavo *et al*, 1993; Rossetto *et al*, 2014; Lam *et al*, 2018). To determine which of these mechanisms was at play, we performed EM on Syt1 KD CRISPRi neurons following BoNT/Ai^wt^‐HRP uptake. Similar to our earlier EM analysis (Fig 1E and F), we observed that the majority of BoNT/Ai^wt^‐HRP internalized into SVs, and to a lesser degree into endo/lysosomes, and APs, in control neurons (Fig 5D and E). However, following Syt1 sgRNA1 KD, we observed a striking phenotype whereby BoNT/Ai^wt^‐HRP was mainly found in tubular structures (Fig 5D and E). These tubular structures often originated from the plasma membrane, where they remained open to the extracellular space (Appendix Fig S8K). This tubular phenotype containing BoNT/Ai^wt^‐HRP was confirmed with electron tomography (Fig 5F, Movie EV4). We next asked whether the tubular phenotype was caused by Syt1 depletion, or by the toxin binding to the neuronal plasma membrane in Syt1‐depleted neurons. We reasoned that if the tubular phenotype was caused by Syt1 depletion alone, we should be able to observe the plasma membrane‐connected tubules by EM using ruthenium red (RuR) plasma membrane staining (Blanquet, 1976). We therefore prepared EM samples of Syt1 sgRNA1 KD hippocampal neurons, stimulated the neurons with high K^+^ buffer with and without 100 pM unlabelled BoNT/Ai^wt^, and stained the neuronal plasma membrane with RuR for EM. In the absence of toxin, we did not observe RuR‐positive tubulation/ invagination of the plasma membrane in the Syt1 KD neurons (Appendix Fig S8L). By contrast, in the presence of toxin, elongated tubular structures stained with RuR could be detected following Syt1 KD, indicating that the tubulation effect was specifically induced by the toxin in these neurons (Appendix Fig S8L).

### Modeling of the tripartite PSG‐Syt1‐SV2 nanocluster for selective binding and endocytic targeting of BoNT/A into SVs

**Figure 6:**
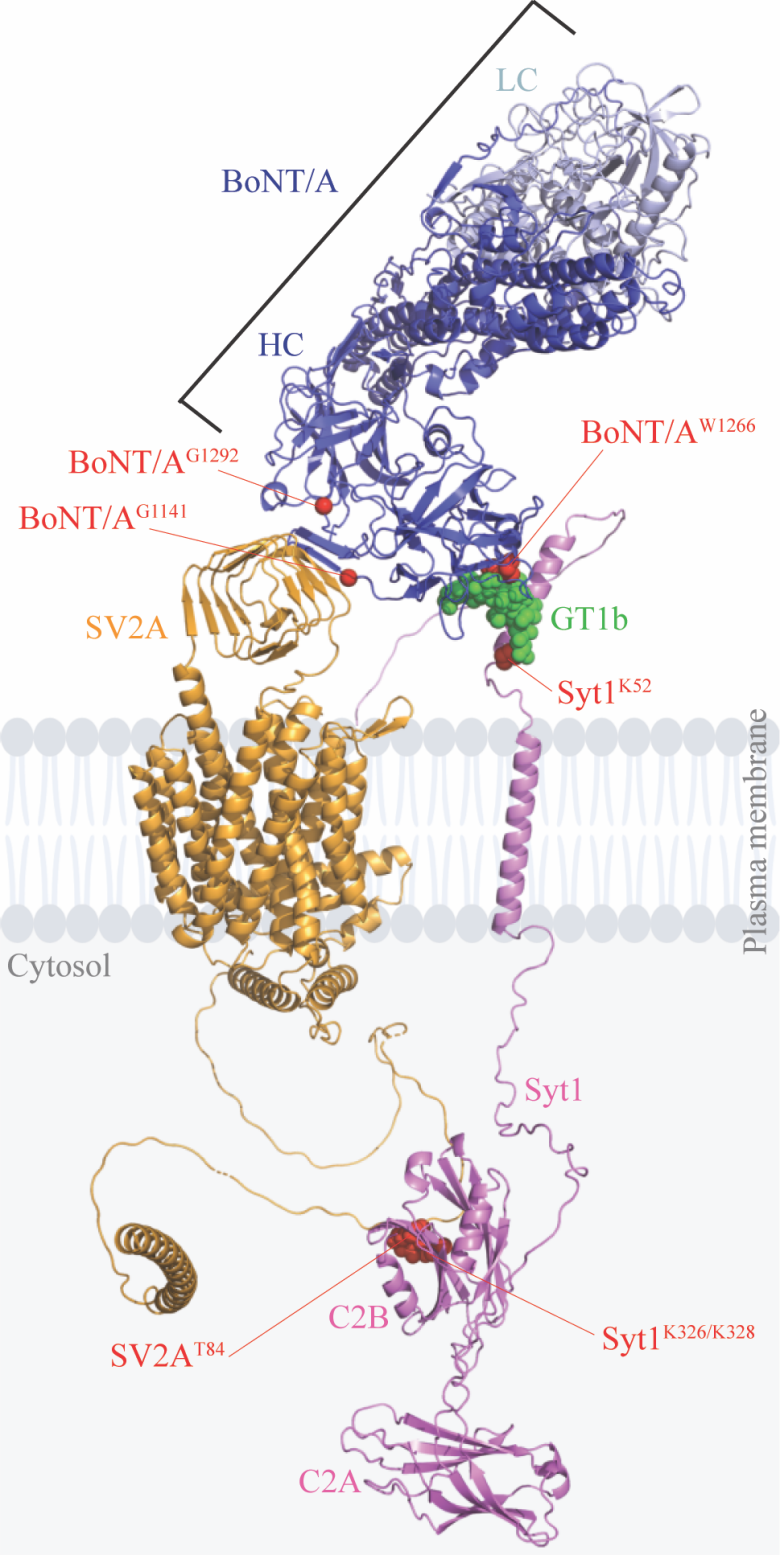
Structural model of the tripartite GT1b‐Syt1‐SV2 complex for selective binding and endocytic targeting of BoNT/A Predictive model of the assembled complex between plasma membrane‐inserted Syt1‐GT1b complex and SV2A, with bound BoNT/A (heavy chain, HC, light chain, LC). Mutated residues in this study are indicated as red spheres.

Together our results point to a model whereby BoNT/A coincidentally binds to both a preassembled PSG‐Syt1 complex and SV2 on the neuronal plasma membrane, thereby facilitating interactions between Syt and SV2 cytoplasmic domains, and the formation of the tripartite PSG‐Syt1‐SV2 complex. We propose a model whereby BoNT/A hijacks an intrinsic tripartite PSG‐Syt1‐SV2 endocytic sorting mechanism present on the neuronal plasma membrane to gain access to SVs. To visualize the tripartite PSG‐Syt1‐SV2 complex together with BoNT/A on the plasma membrane, we created a predictive model of the GT1b ganglioside, Syt1, and SV2 complex together with bound BoNT/A (Fig 6 and Movie EV5). For this, we used the crystal structures of separate complexes, together with the recently published machine learning algorithm for protein structure prediction AlphaFold2 (Jumper *et al*, 2021; Tunyasuvunakool *et al*, 2021). The model, derived from the crystal structures of separate complexes, combined with the AlphaFold2 predictions of full‐length SV2A and Syt1 proteins, points to BoNT/A being able to simultaneously bind to GT1b and SV2A on the plasma membrane. Both SV2A and Syt1 contain significant regions of predicted structural disorder, providing flexible linkers that allow for conformational flexibility in their intracellular binding domains. The simultaneous binding of BoNT/A to GT1b and SV2A allows the formation of Syt1‐SV2 interactions by capturing the GT1b‐bound Syt1 (by the K52 residue), which then interacts with SV2A through K326/K328 and T84 residues in the cytosol, respectively, clustering both receptors and the bound toxin on the plasma membrane. This model suggests that the existence of a tripartite PSG‐Syt1‐SV2 complex is structurally realistic and that this complex could form nanoclusters for the selective binding and endocytic targeting of BoNT/A in SVs.

## Discussion

### PSG binding serves to engage Syt1 during BoNT/A intoxication mechanism

The results presented in this study demonstrate that BoNT/A facilitates its own entry into SVs by hijacking and potentiating an existing tripartite PSG‐Syt1‐SV2 nanocluster that serves as a portal to internalize the toxin in hippocampal neurons. We reveal that BoNT/A recognizes PSGs complexed to Syt1 on the neuronal plasma membrane, which brings Syt1 into the nanoscale vicinity of SV2 and increases the generation of SV2‐Syt1 clusters. Our results further demonstrate that Syt1 expression is rate limiting for BoNT/A intoxication and that Syt1 controls the selective uptake of the toxin into SVs. These findings explain how BoNT/A selectively targets SVs, and update the long‐standing dual receptor model (Montecucco, 1986) to a tripartite model by introducing a third component: Syt1.

Our results reveal that BoNT/A must coincidentally bind to PSG‐Syt1 and SV2 to cause neurointoxication, as binding to SV2 or PSG alone is insufficient for targeted endocytosis into SVs and precludes SNAP‐25 cleavage. Why does BoNT/A initially target two receptors (Montecucco, 1986) on the neuronal plasma membrane? Both Syt1 (Zhang *et al*, 1994; Haucke *et al*, 2000; Grass *et al*, 2004; Walther *et al*, 2004; Diril *et al*, 2006; Yao *et al*, 2011, 2012; Chen *et al*, 2022) and SV2 (Nowack *et al*, 2010; Yao *et al*, 2010; Kaempf *et al*, 2015) have well‐established roles in endocytosis. Syt1‐SV2 iTRAP, on the other hand, has been shown to facilitate the targeting of these proteins back into SVs (Gordon & Cousin, 2016; Cousin, 2017; Harper *et al*, 2020; Small *et al*, 2022). Here we show that BoNT/A binding to the plasma membrane elevates these iTRAP interactions, promoting their own selective uptake into SVs, which ultimately culminates in neuronal intoxication and paralysis. One explanation for the dual binding could therefore be that the simultaneous binding of BoNT/A with PSG‐Syt1 and SV2 can properly orientate these transmembrane proteins so that their cytoplasmic domains can interact and form immobile Syt1‐SV2 nanoclusters that control SV targeting. The immobilization of Syt1 and SV2 must at least partially depend on their ability to interact with one another, given that blocking the interactions abolishes the protein nanoclustering. Interestingly, pull‐down assays have shown that, in addition to SV2, BoNT/A also pulls down the respective iTRAP receptor, Syt1, but not other vesicular proteins (Peng *et al*, 2011), supporting an association between Syt1 and SV2 in the BoNT/A intoxication process (Bennett *et al*, 1992; Schivell *et al*, 1996; Lazzell *et al*, 2004; Baldwin & Barbieri, 2007; Yao *et al*, 2010).

Downstream of these initial toxin binding and endocytic sorting events on the plasma membrane, CNTs are mainly endocytosed in clathrin‐coated vesicles (Maksymowych & Simpson, 2004; Deinhardt *et al*, 2006; Couesnon *et al*, 2009; Harper *et al*, 2011, 2016; Colasante *et al*, 2013; Meng *et al*, 2013; Pellett *et al*, 2015; Wang *et al*, 2015). Clathrin‐coated pits are formed by the association of an array of endocytic proteins including clathrin heavy and light chains, as well as AP‐2, and are further regulated by membrane lipid phosphatidylinositol 4,5‐bisphosphate (PIP2) (Haucke, 2005). Syt1 has been shown to bind to PIP2‐containing membrane patches (Park *et al*, 2015) which are dephosphorylated by synaptojanin‐1 to facilitate membrane fission (Chang‐Ileto *et al*, 2011). Whether Syt1 and SV2 form nanoclusters on the plasma membrane patches that are enriched in PIP2 remains to be determined. Our results suggest that the endocytic sorting of the toxin depends on tripartite interactions within the PSG‐Syt1‐SV2 nanocluster. It is likely that downstream of these events, other endocytic machinery proteins, such as stonin‐2 (Zhang *et al*, 1994; Haucke *et al*, 2000; Grass *et al*, 2004; Walther *et al*, 2004; Diril *et al*, 2006), are also involved in the uptake of BoNT/A.

Our single‐molecule tracking of BoNT/Ai^PSG^‐At647N and BoNT/Ai^PSG,SV2^‐At647N on the plasma membrane (uPAINT) revealed a significantly decreased binding of the toxin compared with wildtype toxin. Unexpectedly, BoNT/Ai^PSG,SV2^‐At647N binding to the plasma membrane did not show a further decrease compared with the other receptor‐binding mutant holotoxins. This could be due to the slightly elevated At647N‐labeling of BoNT/Ai^PSG,SV2^ compared with the other mutant holotoxins (Appendix Figs S1E and S2A) increasing the positive charge of the toxin molecule, thereby potentially increasing its nonspecific binding of the toxin. Our analysis on the binding of At647N‐labeled mutant toxins to glass‐bottom dishes showed no differences in the number of bound toxins (Appendix Fig S8M), indicating that the increased At647N labeling was not contributing to the BoNT/Ai^PSG,SV2^‐At647N binding. Additional factors, such as FGFR3 (James *et al*, 2021) which interacts with SV2 (Jacky *et al*, 2013), have also been suggested to have a role in BoNT/A neurointoxication, which could offer an alternative explanation as to why the BoNT/Ai mutant, with abolished binding to both of its primary receptors, was still able to bind neuronal plasma membrane. However, based on our results, potential engagement with additional receptors without PSG and SV2 binding resulted in unsuccessful endocytic uptake of the toxin into SVs, and therefore the role of additional factors remains to be determined.

### Syt1‐SV2 iTRAP controls the endocytic targeting of BoNT/A into SVs

Laterally diffusing plasma membrane proteins (Kusumi *et al*, 1993; Borgdorff & Choquet, 2002; Dahan *et al*, 2003; Heine *et al*, 2008) and receptors agglomerate in clusters that are crucial for enhancing pathogen‐binding capability, indicating that hierarchical clustering correlates with function (Garcia‐Parajo *et al*, 2014). The newly generated SVs formed during endocytosis must contain the appropriate proteins with the correct stoichiometry to remain fusion competent and ultimately maintain neurotransmission in nerve terminals. The synaptic iTRAPs are a major determinant of vesicular protein stoichiometry in SVs, where specific SV proteins themselves provide a final “fail‐safe” to ensure accurate retrieval of SV proteins and bound cargo into SVs (Cousin, 2017). We recently demonstrated that Syt1‐SV2A nanoclusters control the recycling of SVs, as well as the selective retrieval of plasma membrane‐stranded vesicular proteins into SVs (Small *et al*, 2022). Depletion of SV2A results in a plasma membrane retrieval defect of Syt1 without affecting other SV proteins (Yao *et al*, 2010; Zhang *et al*, 2015), indicating that the trafficking of these proteins is highly interconnected. Our PLA experiments suggest that Syt1 and SV2C interact or form iTRAPs in untreated neurons, thereby ensuring that proteins, which perform essential functions in SV exocytosis, such as Syt1 and SV2, are clustered and retrieved efficiently (Harper *et al*, 2020; Small *et al*, 2022). We also found that BoNT/A treatment significantly increases the formation of Syt1‐SV2 iTRAPs on the neuronal plasma membrane, which most likely reflects the molecular rearrangement of the iTRAPs prior to their internalization into SVs. Taken together, the findings presented here support a model whereby BoNT/A elevates the formation of Syt1‐SV2 interactions via coincidental binding to the PSG‐Syt1 complex and SV2. BoNT/A thereby promotes its own internalization into SVs by facilitating the nanoclustering of SV2 and Syt1. Receptor‐binding mutations in BoNT/A lead to perturbed toxin and receptor nanoclustering on the plasma membrane, which causes a deficiency in the endocytic uptake of the toxin and disrupts its subsequent sorting into SVs.

Pathological cargoes, such as viruses and toxins, have been shown to affect the nanoscale receptor clustering on the plasma membrane (Aigal *et al*, 2015), and target multimeric receptor clusters (Finke *et al*, 2020; Kabbani *et al*, 2020; Sieben *et al*, 2020). Human papillomaviruses, for example, target multimeric receptor clusters on the host cell plasma membrane, where the various nanocluster components have different functional roles in the binding, uptake, and infectivity of the virus (Finke *et al*, 2020). Another example is the influenza A virus, which targets multivalent sialic acid clusters on the plasma membrane (Sieben *et al*, 2020). These clusters overlap with epidermal growth factor receptor clusters that the virus needs to activate downstream factors in order to trigger its endocytic uptake (Sieben *et al*, 2020). Cholera toxin subunit B, on the other hand, can induce membrane curvature and endocytosis only when bound to multiple copies of its glycosphingolipid receptor, GM1, within a cluster (Kabbani *et al*, 2020). Small changes in receptor clustering can control whether or not a receptor and bound cargo are internalized efficiently (Salavessa *et al*, 2021). For example, interleukin‐2 receptor clustering at the cell surface was recently shown to be fundamental for the efficient internalization of the receptor, with small clusters (≤ 3 receptors) representing newly secreted and/or abortive endocytic events, medium clusters (4–6 receptors) being endocytic competent, and large clusters (> 6 receptors) having stalled (Salavessa *et al*, 2021). Such reports, together with our results, support a model whereby external cargo such as BoNT/A can facilitate cell surface protein nanoclustering. Here, we show that genetic perturbation of the intermolecular interactions between PSG, Syt1 and SV2, or ablation of the coincidental binding of BoNT/A to PSG and SV2, alter SV2A and Syt1 nanoclustering, indicating that receptor clustering on the plasma membrane is tightly controlled through these intermolecular interactions, and that receptor nanoclustering opens a gateway for the toxin to reach SVs as part of its neurointoxication strategy.

### Syt1 depletion leads to BoNT/A mistargeting into tubular structures, and loss of toxic function

Our results indicate that Syt1 functions as an endocytic sorting factor, together with SV2, directing BoNT/A into SVs. Following Syt1 depletion, BoNT/Ai‐HRP is mislocalized into tubular structures that are connected to the plasma membrane. Interestingly, the C2A–C2B domains of Syt1 were shown to convert liposomes into long, thin lipid tubules in the presence of 1 mM Ca^2+^, indicating that Syt1 may have a propensity to promote membrane tubulation (Chen *et al*, 2022). Using plasma membrane RuR staining and EM, we further demonstrate that these tubules are only observed in Syt1 KD neurons following treatment with BoNT/Ai^wt^, indicating that the formation of these tubular structures requires the presence of the toxin as no effect was observed in the absence of toxin. It is worth noting that RuR only stains the tubular membranes if the tubule remains connected to the plasma membrane. It is therefore possible that some of the tubules could have lost their connection to plasma membrane and were not detected with RuR staining. However, it is unlikely that all the tubules would have lost this connection as we regularly observed HRP‐labeled toxins in tubules connected to the plasma membrane. Based on these results, it is possible that the plasma membrane‐connected tubular structures share the neutral pH of the extracellular space. In the instance that fission of tubular endosomes occurs, vacuolar‐type ATPase (vATPase)‐driven endosomal acidification should take place, which is a requirement for BoNT/A intoxication. Together our results suggest that the decreased SNAP‐25 cleavage following Syt1 KD stems from the accumulation of BoNT/A in surface‐connected tubules which presumably lack the acidic environment required for translocation to the cytosol (Koriazova & Montal, 2003; Mesaki *et al*, 2011).

### The PSG‐Syt1‐SV2 complex as a common entry mechanism for the majority of CNTs

The majority of CNTs bind to two receptors, leading to irreversible plasma membrane binding (Rummel, 2017). Interestingly, BoNT/A, /B, /E, /F, /G, and TeNT bind to PSGs via a conserved PSG‐binding site E(Q)…H(K/G)…SXWY…G (Rummel, 2013), suggesting that the preassembled PSG‐Syt1 complex may be involved in the initial binding of these CNTs to the plasma membrane. Our results indicate that Syt1 has a role in the neurointoxication process of both BoNT/A and BoNT/E, which target PSGs and SV2 as their receptors. It is therefore tempting to hypothesize a “unifying mechanism” for those CNTs that share this conserved PSG‐binding site, allowing these toxins to hijack the tripartite PSG‐Syt1‐SV2 complex to initiate their selective endocytic uptake into SVs. However, alternative assembly options may also be possible. BoNT/B ganglioside‐binding site 1 was recently shown to interact with Syt‐free GD1a, while the Syt‐binding pocket was demonstrated to interact with GT1b precomplexed with Syt (Ramirez‐Franco *et al*, 2022). In a separate study the preassembled GT1b/Syt complex was shown to be crucial for the binding and entry of BoNT/B (Flores *et al*, 2019). These results suggest that BoNT/B has interactions with multiple gangliosides, supporting the model whereby the toxin recognizes a protein/ganglioside complex. Further studies are needed to establish whether BoNT/A also engages with the membrane‐inserted PSG‐Syt1 complex alongside free gangliosides. Together, the results presented here unveil a fundamental endocytic targeting mechanism of receptor‐mediated cargo uptake in primary neurons. Our findings also provide a detailed nanoscale model for the early steps by which the tripartite PSG‐Syt1‐SV2 complex is hijacked by a bacterial toxin to form nanoclusters that control its selective entry into SVs.

## Materials and Methods

### Key resources

Key resources are listed in Table EV1.

### Ethical considerations and animals

Experiments were conducted with the approval of The University of Queensland Animal Ethics Committee (Approval #2016/AE000254 and 2019/AE000244). Pregnant Sprague Dawley rats (Charles River Laboratories; https://www.criver.com) were individually housed and maintained in a dedicated animal husbandry area at room temperature (~ 22°C) in pathogen‐controlled laboratory conditions. Rats were kept on a 12:12 h light/dark cycle and received water and food *ad libitum*.

### Neuronal cultures

All experiments were conducted on hippocampal neurons obtained from rats at embryonic day 18 (E18) as previously described (Joensuu *et al*, 2017). Both male and female embryos were used for all experiments. Dissected hippocampal neurons were cultured in neuronal culture medium, composed of neurobasal medium (Gibco‐Thermo Fisher, Cat. #12348‐017), 1× serum‐free B27 supplement (Gibco‐Thermo Fisher, Cat. #17504‐044), 1× GlutaMAX supplement (Gibco‐Thermo Fisher, Cat. #35050‐061) and a penicillin (100 U ml^−1^)/streptomycin (100 μg ml^−1^, Invitrogen‐Thermo Fisher, Cat. #15140‐122) mix as previously described (Joensuu *et al*, 2017), on poly‐L‐lysine–coated (Sigma‐Aldrich, Cat. #P2636) glass‐bottom dishes at 80,000 (CellVis, Cat. # D29‐20‐1.5‐N for all super‐resolution imaging), and 30,000–50,000 (CellVis, Cat. #D29‐10‐1.5‐N for proximity ligation assay, CRISPR interference and electron microscopy) cells per coverglass. For electrophysiology, hippocampal neurons were plated at 50,000 cells per poly‐L‐lysine–coated 5‐mm coverslip placed in D29‐10‐1.5‐N glass‐bottom wells. All transfections were performed on day *in vitro* 14 (DIV14), or on DIV7 for shRNA Syt1 KD experiments, using Lipofectamine 2000 reagent (Invitrogen, Cat. #11668019) according to the manufacturer's instructions. CRISPRi lentiviral transduction was performed on DIV14 (for 7 days) and subsequent rescue experiments on DIV19 (for 48 h). All live‐cell imaging and sample processing were performed on mature neurons on DIV21‐22.

### Mammalian expression constructs

The pHluorin‐Syt1 K52A (Syt1^K52A^‐pH) construct was created by site‐directed mutagenesis as per the manufacturer's instructions (QuikChange Lightning Site‐Directed Mutagenesis Kit, Agilent Technologies, Cat. #210518). The mutagenesis primers used were Forward: 5′‐GTTTATGAATGAGCTGCATGCAATTCCATTGCCACCGTG 3′ and Reverse: 5′‐CACGGTGGCAATGGAATTGCATGCAGCTCATTCATAAAC 3′. The construct was sequenced in the Australian Genome Research Facility at The University of Queensland. Synaptotagmin‐1 shRNA was inserted into pSUPER‐neo + GFP (with GFP replaced by mCerulean; Clayton *et al*, 2010). pSuper‐neo + mCerulean was cut with BgIII and XhoI to insert annealed shRNA oligonucleotides by T4 ligation; the BgIII site is destroyed by this process.

### Lentiviral CRISPRi and rescue plasmids

In the CRISPRi system, the nuclease null form Cas9 (dCas9) was fused to three different single guide RNAs (sgRNA1‐3) and to the transcription repressor KRAB (dCas9‐KRAB) to block Syt1 gene transcription. This technique avoids the use of insertions or deletions (indels) which guarantees the genotypic consistency of neurons following gene suppression, thereby making CRISPRi particularly suitable for Syt1 KD in the neurons, as demonstrated previously (Chen *et al*, 2021). All sgRNAs for rat Syt1 wildtype and K52A were constructed on the same backbone vector (#AAAA‐0244, termed “dCas9‐KRAB‐TagBFP2”; Table EV3) by DNA Dream Lab. We used the pLV hU6‐sgRNA hUbC‐dCas9‐KRAB‐T2a‐Puro construct (dCAS9‐KRAB; a gift from Charles Gersbach, Addgene plasmid #71236; http://n2t.net/addgene:71236; RRID: Addgene_71236 (Thakore *et al*, 2015)), where the puromycin‐resistance gene was replaced by the TagBFP2 (tag blue fluorescent protein 2) marker, which was PCR‐amplified from the ACE2 g1 + Cas9 plasmid (a gift from Jason Sheltzer, Addgene plasmid #153011; http://n2t.net/addgene:153011; RRID: Addgene_153011). The replacement was made with the NEBuilder kit (NEB, Cat. #E2621S) by concatenating the PCR‐linearized dCAS9‐KRAB construct and TagBFP2 fluorescent marker with the primers indicated in Table EV2. The final backbone expresses gRNA under the human U6 promoter, with dCAS9‐KRAB being fused with self‐cleaving peptide (via T2A) and TagBFP2 fluorescent marker, all under the UbC promoter. To find the targets for gRNAs on the 5′‐UTR of the rat Syt1 gene, we used Chop‐Chop (Montague *et al*, 2014; Labun *et al*, 2016, 2019) (https://chopchop.cbu.uib.no) and CRISPick (Kim *et al*, 2018; DeWeirdt *et al*, 2021) (aka GPP; https://portals.broadinstitute.org/gppx/crispick/public) and Benchling softwares (Benchling, https://www.benchling.com/molecular‐biology/). We selected 3 locations manually and designed oligonucleotides with CACC and AAAC overhangs on the 5′ position of the forward and reverse primers, respectively, for cloning into the dCas9‐KRAB‐TagBFP2 vector. Each pair of primers was annealed and ligated into the BsmBI‐linearized dCas9‐KRAB‐TagBFP2 vector following the manufacturer's instructions. The resulting plasmids are listed in Table EV3 (Syt1 sgRNA1 #AAAA‐0245, Syt1 sgRNA1 #AAAA‐0246, and Syt1 sgRNA1 #AAAA‐0247). All the sequences were confirmed by Sanger sequencing to match the original design. For Syt1 rescue experiments, the pLenti6.3‐Syt1^wt^‐pH vector (#AAAA‐0238; Table EV3) with N‐terminal pHluorin‐tag was produced by assembling the PCR‐linearized *Rattus norvegicus* Syt1 gene under the human synapsin1 promoter as one DNA fragment from the pSUPER‐pHluorin‐Syt1 (Syt1^wt^‐pH) plasmid and PCR‐linearized pLenti6.3 vector, using NEBuilder HiFi DNA Assembly Master Mix (NEB, Cat. #E2621), following the standard manufacturer's protocol. The existing CMV promoter in the original pLenti6.3 was eliminated during the PCR step. We used KAPA HiFi HotStart ReadyMix (Roche, Cat. #7958927001) DNA polymerase for the PCR reactions according to the manufacturer's protocol. In a similar manner, a variant of the synaptotagmin‐1 gene carrying the K52A mutation was transferred to pLenti6.3, also under the human synapsin1 promoter, and was assigned #AAAA‐0239 as a unique identifier (Table EV3).

### Production of inactivated BoNT/Ai holotoxins

Enzymatically inactivated, full‐length BoNT/Ai^wt^ (E224A/R363A/Y366F; Gu *et al*, 2012) and the respective receptor‐binding mutant BoNT/Ai^PSG^ (E224A/R363A/Y366F + W1266L), BoNT/Ai^SV2^ (E224A/R363A/Y366F + G1141D/G1292R) and BoNT/Ai^PSG‐SV2^ (E224A/R363A/Y366F + W1266L/G1141D/G1292R) holotoxin were produced recombinantly in the *E. coli* BL21 DE3 strain (NEB, Cat. #C2527H) in the laboratory of Dr. Andreas Rummel. All mutations were generated by two‐step PCR and verified by DNA sequencing. Inactive BoNT/Ai^wt^ and mutants carrying a C‐terminal His6‐tag were purified on Co^2+^‐Talon matrix (Takara Bio Europe S.A.S.) and eluted with 50 mM Tris–HCl (pH 8.0), 150 mM NaCl, and 250 mM imidazole. For proteolytic activation and removal of the affinity tag, BoNTs were incubated for 16 h at room temperature with 0.01 U bovine thrombin (Sigma‐Aldrich) per μg of BoNT. Subsequent gel filtration (Superdex‐200, Cytiva) was performed in phosphate‐buffered saline (PBS, pH 7.4). For storage, enzymatically inactivated, full‐length BoNT/Ai^wt^ and mutants were snap‐frozen in liquid nitrogen and maintained at −80°C.

### Production of active BoNT/A for mouse phrenic nerve hemidiaphragm assay

Active BoNT/A was produced recombinantly in BL21(DE3) *E. coli* (NEB, Cat. #C2527H) as previously described (Weisemann *et al*, 2015) under biosafety level 2 containment (project number GAA A/Z 40654/3/123), yielding a solution of 2.5 μM (0.37 mg ml^−1^) BoNT/A in PBS buffer. To obtain a high degree of labeling (DoL; manufacturer recommended protein concentrations > 1 mg ml^−1^), 10 nmol of BoNT/A were concentrated by ultrafiltration (MWCO 30 kDa) by a factor of ~ 4 to yield a solution of 11.5 μM (1.7 mg ml^−1^) BoNT/A.

### At647N labeling of active and inactivated BoNT/A and determination of the degree of labeling

To map the potential At647N‐labeling sites in the toxin structure, the BoNT/A lysine (K) residues, together with point mutations inactivating the catalytic activity (E224A/R363A/Y366F) and receptor‐binding mutations (W1266L/G1141D/G1292R), were indicated in the ribbon structure of BoNT/A holotoxin using the Pymol program (Schrodinger, https://pumol.org/2/) (Appendix Fig S2A). This demonstrated a homogenous distribution of the 103 lysine residues throughout the BoNT/A structure, and we assume a random coupling of At647N to these residues. BoNT/Ai^wt^, BoNT/Ai^W1266L^ (BoNT/Ai^PSG^), BoNT/Ai^G1141D/G1292R^ (BoNT/Ai^SV2^), and BoNT/Ai^W1266L/G1141D/G1292R^ (BoNT/Ai^PSG,SV2^) holotoxins were labeled with Atto647N NHS‐ester (At647N; ATTO‐TEC Gmbh, Cat. #AD 647 N‐31) according to the manufacturer's protocol. Briefly, At647N was dissolved in water‐free DMSO and the pH of BoNT/Ai dilution in PBS buffer was adjusted to 8.3 with 0.2 M NaHCO_3_. A 3:1 (molar) ratio of At647N‐dye: BoNT/Ai was mixed, shielded from light, and incubated for 1.5 h at room temperature in slow rotation. The nonbound dye was then removed using Sephadex G‐25 columns (Cytiva, Cat. #C985C42) and the first blue fraction containing the labeled At647N‐BoNT/A was collected, aliquoted, and stored at −80°C.

The DoL of BoNT/Ai^wt^‐At647N and the receptor‐binding mutants was first quantified in a cell‐free flow‐chamber assay by quantifying the number of At647N fluorescence emission steps over time, as previously described (Joensuu *et al*, 2016, 2017). The average number of emission steps was 1.7 ± 1.1 (SD) for BoNT/Ai^wt^‐At647N, 1.9 ± 0.8 for BoNT/Ai^PSG^‐At647N, 1.7 ± 1.1 for BoNT/Ai^SV2^‐At647N, and 3.2 ± 1.5 for BoNT/Ai^PSG,SV2^‐At647N, indicating that each BoNT/A molecule was labeled with one to three At647N molecules. The DoL was then confirmed using a NanoDrop ND 1000 (Thermo Scientific) spectrophotometer (Appendix Fig S3E) using the equation (where A_280_ is the absorption maximum of BoNT/A, and A_647_ is the absorption maximum of At647N):

$$\mathrm{DoL}=\left(A647\times \frac{\text{εBoNTA}}{\left(A280-A647\times \mathrm{CF}280\right)\times \text{εmax}}\right).$$

The extinction coefficient of inactivated BoNT/A at 280 nm (εBoNTA) was calculated as follows (where *n*(W), *n*(Y), and *n*(C) are the number of tryptophans, tyrosines, and cysteines in the BoNT/A amino acid sequence, respectively):

$$\text{εBoNTA}=n\left(W\right)\times 5,500+n\left(Y\right)\times 1,490+n\left(C\right)\times 125\times \frac{1}{M}\times \mathrm{cm}.$$

Using Nanodrop ND 1000's Proteins & Labels test type, a new dye, At647N, with the following specifications was created: CF_260_(At647N) = 0.04; CF_280_(At647N) = 0.03; λ_abs_ = 647 nm; ε_max_ = 1.5 * 10^5^ * 1/(M^−1^ cm^−1^). The sample type was set to other protein (E 1%) BoNT/A ε^1%^ = (ε_BoNT/A_ * 10) / M_w_ = 192,395/150,000 = 12.83. The absorption was measured at 280 and 647 nm from three to four technical replicates, with the estimated average DoL being 1.68 for BoNT/Ai^wt^‐At647N, 1.83 for BoNT/Ai^PSG^‐At647N, 2.17 for BoNT/Ai^SV2^‐At647N, and 3.5 for BoNT/Ai^PSG,SV2^‐At647, supporting the results obtained from the cell‐free flow‐chamber assay.

At647N labeling of active BoNT/A was performed as described above and analyzed with SDS–PAGE together with nonlabeled active BoNT/A, demonstrating no apparent change in the size of the toxins following the labeling (Appendix Fig S2C). The BoNT/A‐At647N DoL was then determined using a NanoDrop 1000 spectrophotometer as described above (Appendix Fig S2E), indicating that on average BoNT/A‐At647N contained one or two At647N‐fluorophores (average DoL 1.46). Assessment of the binding of the BoNT/Ai^PSG^‐At647N, BoNT/Ai^SV2^‐At647N, and BoNT/Ai^PSG,SV2^‐At647 to poly‐L‐lysine–coated glass‐bottom cell culture dishes was done as follows: The toxins were diluted into low K^+^ buffer (0.5 mM MgCl_2_, 2.2 mM CaCl_2_, 5.6 mM KCl, 145 mM NaCl, 5.6 mM D‐glucose, 0.5 mM ascorbic acid, 0.1% (wt/vol) BSA and 15 mM HEPES, pH 7.4, 290–310 mOsm) to a final concentration of 100 pM, and the dilution was added onto the culture dish glass‐bottom for 20 min (at room temperature, in the dark). The dish was then gently washed 3 times with low K^+^ buffer. Imaging of 16–12 random regions of interest for each toxin was then performed as described below for uPAINT. The number of glass‐bottom‐bound toxins was then counted manually, and related to the area (μm^2^).

### HRP labeling of holotoxins

A Lightning‐Link HRP Antibody Labelling Kit (Novus Biologicals, Cat. #701‐0000) was used to conjugate the inactivated BoNT/Ai^wt^, BoNT/Ai^PSG^, BoNT/Ai^SV2^, and BoNT/Ai^PSG,SV2^ holotoxins with HRP. For the random direct conjugation of HRP to any available amine group in the toxin structure, the labeling was performed according to the manufacturer's instructions in a 1:4 molar ratio of holotoxin:HRP. All conjugated toxins were stored at −20°C with the addition of 50% glycerol as a stabilizer.

### Lentiviral production

For lentiviral production, 5.0 × 10^6^ Lenti‐XTM 293T Cell Line (Takara Bio, Cat. #632180) cells were seeded into T175 flasks (Nunc, Cat. #159910) in 35 ml of Dulbecco's modified Eagle's medium (DMEM) with 4.5 g l^−1^ glucose (Gibco, Cat. #11995‐065), 10% fetal bovine serum (FBS; Gibco, Cat. #26140‐079), 100 U ml^−1^ penicillin/streptomycin (Invitrogen‐Thermo Fisher, Cat #15140‐122), and 1× L‐glutamine (Gibco, Cat. # 25030). The following day, 60–70% of confluent cells were transfected with Transit LT‐1 (Mirus, Cat. #2304) according to the manufacturer's instructions. Briefly, 150 μl of Transit LT‐1 was diluted into 6 ml of Opti‐MEM (Gibco, Cat. #31985‐070) and 50 μg (total) of the following plasmids were separately diluted in Opti‐MEM: pMDLg/pRRE (a gift from Didier Trono, Addgene plasmid #12251; http://n2t.net/addgene:12251; RRID:Addgene_12251 (Dull *et al*, 1998)), pRSV‐REV (a gift from Didier Trono, Addgene plasmid #12253; http://n2t.net/addgene:12253; RRID:Addgene_12253 (Dull *et al*, 1998)), pMD2.G (a gift from Didier Trono, Addgene plasmid #12259; http://n2t.net/addgene:12259; RRID: Addgene_12259), and either the CRISPRi plasmid encoding TagBFP2 and the dCas9‐KRAB, or one of the sgRNA1‐3 guides, or rescue plasmid encoding pLenti6.3‐Syt1^wt^‐pH or pLenti6.3‐Syt1^K52A^‐pH (see Table EV3). The plasmid ratio for pMDL g/p RRE, pRSV‐REV, pMD2‐G, and the CRISPRi/rescue constructs was 1:1:0.5:2.5, respectively. After 5 min, the two solutions were mixed and incubated for 20 min at room temperature. The mix was then added to the cells drop by drop and the cells were incubated for 8 h at 37°C in 5% CO_2_ atmosphere, after which the culture medium was replaced with complete growth medium (without antibiotics). 48 h after transfection, the medium was collected and centrifuged at 3,800 *g* for 15 min at 4°C. The supernatant was then centrifuged at 100,000 *g* in a SW32Ti rotor for 2 h at 4°C, over a 4.5 ml cushion of 20% sucrose prepared in neurobasal medium. The pellet was finally resuspended in 150 μl of neurobasal medium overnight at 4°C with slow rocking. Aliquots were frozen at −80°C.

### Syt1 KD and AbobotulinumtoxinA and BoNT/E treatment

For the assessment of AbobotulinumtoxinA and BoNT/E toxic function following Syt1 KD with CRISPRi and shRNA, E18 hippocampal neurons were cultured on glass‐bottom dishes (CellVis, Cat. #D29‐10‐1.5‐N) at 50,000 neurons per 78.54 mm^2^ confluency to ensure mature and functional synaptic connections. For CRISPRi Syt1 KD, neurons (DIV14) were transduced with dCas9‐KRAB/TagBFP2 (control) or three different CRISPRi Syt1‐targetting sgRNAs (sgRNA1‐3) that were also designed to produce TagBFP2 marker, for 7 days. The lentiviral transduction of the neurons was performed as follows: 20 μl of the respective lentiviral stocks were diluted into 30 μl of conditioning medium collected from the dishes, with the remaining conditioning medium being collected into a sterile 15 ml Falcon tube. The virus mix was added onto the neurons, and incubated for 4–6 h at 37°C in a 5% CO_2_ atmosphere, after which the remaining conditioning medium was added back onto the dishes. For rescue experiments, the neurons were subsequently transduced with pLenti6.3‐Syt1wt‐pH on DIV19 for 72 h as described above. On DIV21‐22, the conditioning medium was collected from the dishes, and the neurons were stimulated with high K^+^ buffer (56 mM KCl; Ajax Finechem Pty Limited, Cat. #1206119), 0.5 mM ascorbic acid (Sigma‐Aldrich, Cat. #A5960), 0.1% bovine serum albumin (BSA; Sigma‐Aldrich, Cat. #A8022), 15 mM HEPES (Sigma‐Aldrich, Cat. #H3375), 5.6 mM D‐glucose (AMRESCO, Cat. #0188), 95 mM NaCl (Sigma‐Aldrich, Cat. #S9888), 0.5 mM MgCl_2_ (Chem‐Supply, Cat. #MA029), and 2.2 mM CaCl_2_ (Sigma‐Aldrich, Cat. #C5080; at pH 7.4, 290–310 mOsm) supplemented with 10 U of AbobotulinumtoxinA (Ipsen; Dysport®; BoNT/A‐based licensed drug) or 10 nM BoNT/E (a kind gift from Thomas Binz) for 5 min at 37°C in 5% CO_2_ atmosphere. The neurons were then washed three times with low K^+^ buffer, and chased for 30 min/ 16 h (AbobotulinumtoxinA), or 16 h (BoNT/E), in conditioning medium at 37°C in a 5% CO_2_ atmosphere. Samples were then either fixed for immunofluorescence staining or processed for patch‐clamp electrophysiology, as described below. The use of AbobotulinumtoxinA and BoNT/E was approved by The University of Queensland Institutional Biosafety Subcommittee #IBC/562B/QBI/2022.

### Immunofluorescence staining of hippocampal neurons

For the assessment of AbobotulinumtoxinA‐ and BoNT/E‐induced cleavage of SNAP‐25, hippocampal neurons grown on glass‐bottom dishes were lentivirally transduced with nontargeting (control) or Syt1 sgRNA1 CRISPRi for 7 days and then treated with catalytically active toxins as described above. They were then fixed with 4% paraformaldehyde (Electron Microscopy Sciences, Cat. #15710) in PBS for 20 min at room temperature, followed by permeabilization with 0.1% of Triton X‐100 (Sigma) in PBS for 3 min at room temperature, and blocking with 1% BSA (Sigma) in PBS for 45 min at room temperature. After washing three times with PBS, samples were incubated with primary antibodies diluted in blocking solution overnight at 4°C: Syt1 (Abcam, Cat. #13259; 1:100 dilution in blocking buffer), BoNT/A‐cleaved SNAP‐25 (anti‐SNAP‐25/A used at a concentration of 87.5 μg ml^−1^ diluted in blocking buffer), or BoNT/E‐cleaved SNAP‐25 (anti‐SNAP‐25/E used at a concentration of 150 μg ml^−1^ diluted in blocking buffer). Antibodies recognizing the cleaved SNAP‐25 were a kind gift from D. Sesardic and P. Stickings, Division of Bacteriology, National Institute for Biological Standards and Control, United Kingdom. After three washes with PBS, samples were incubated in the following secondary antibodies diluted in blocking buffer, covered from light, for 30 min at room temperature: anti‐mouse Alexa‐546 (Invitrogen, Cat #A11030; 1:1,000) and anti‐rabbit Alexa‐647 (Invitrogen, Cat. #A32733). They were then washed with PBS, mounted in ProLong Gold Mountant (Invitrogen, Cat #P10144), and imaged using a spinning‐disk confocal system (Marianas; 3I, Inc.) consisting of an Axio Observer Z1 (Carl Zeiss) equipped with a CSU‐W1 spinning‐disk head (Yokogawa Corporation of America), ORCA‐Flash4.0 v2 sCMOS camera (Hamamatsu Photonics), and 20× 0.8 NA / Plan‐Apochromat/550 μm WD objective. Image acquisition was performed using SlideBook 6.0 (3I, Inc). The CRISPRi results presented are from *n* = 21 random regions of interest (ROIs) for both conditions, originating from two independent experiments, and the dot plots indicate the average fluorescence intensity ± SEM of endogenous Syt1 and SNAP‐25/A. The results for BoNT/E‐induced cleavage of SNAP‐25 in control and Syt1 sgRNA KD neurons are from *n* = 9 random ROIs for both conditions, 2 technical replicas originating from one independent experiment, and the dot plots indicate the average fluorescence intensity±SEM of endogenous Syt1 and SNAP‐25/E. The pLenti6.3‐Syt1^wt^‐pH rescue experiments are from *n* = 3 random ROIs of both Syt1 sgRNA and control conditions (one independent experiment), and the dot plots indicate the average fluorescence intensity ± SEM of endogenous Syt1 and cleaved SNAP‐25/A. The pLenti6.3‐Syt1^K52A^‐pH rescue experiments are from *n* = 3 random ROIs of Syt1 sgRNA conditions (one experiment), and the dot plots indicate the average fluorescence intensity ± SEM of anti‐Syt1 antibody labeling and cleaved SNAP‐25/A.

For co‐localization analysis of SV2A with the plasma membrane marker Ezrin, rat E18 hippocampal neurons cultured on glass coverslips were lentivirally transduced with control (nontargeting) and Syt1 sgRNA1 for 7 days, and at DIV22 were fixed, permeabilized, and blocked as described above, then immunostained against endogenous SV2A (Abcam, Cat. #ab77177; 1:50 dilution in blocking buffer) and Ezrin (Abcam, Cat. #ab40839; 1:50 dilution in blocking buffer), as well as Syt1 (Abcam, Cat. #ab13259; 1:50 dilution in blocking buffer) for 16 h at 4°C. Samples were then washed and stained with the following secondary antibodies: donkey anti‐goat Alexa Fluor 488 (ThermoFisher Scientific, Cat #A‐11055; 1:1,000 dilution), goat anti‐rabbit Alexa Fluor 647 (ThermoFisher Scientific, Cat #A‐21245; 1:1,000 dilution) and donkey anti‐mouse Alexa 568 (Abcam, Cat. #175472; 1:1,000 dilution). Mounted samples were imaged with a spinning‐disk confocal microscope as described above with the exception of using a 63× 1.4 NA/Plan‐Apochromat/180 μm WD oil objective. Acquired stacks were deconvolved with Huygens Professional 19.10 software (Scientific Volume Imaging; https://svi.nl/Huygens‐Professional), and the Pearson co‐localization of SV2A and Ezrin was quantified using Imaris Software (Oxford Instruments; https://imaris.oxinst.com/). The results shown are from *n* = 20 random ROIs of control and Syt1 sgRNA KD neurons, from two independent experiments, and the dot plot indicates the Pearson correlation coefficients of SV2A and Ezrin from each acquisition. Immunolabeling against Syt1 is shown in the small inset of the representative images.

To assess the SV2A availability for BoNT/A binding on the plasma membrane following Syt1 KD, the fluorescence intensity of BoNT/Ai^PSG^‐At647N (our BoNT/Ai mutant with ablated binding to PSG, and therefore only binds to SV2) bound to the neuronal plasma membrane was quantified. For this, E18 hippocampal neurons were lentivirally (nontargeting sgRNA and Syt1 sgRNA1) transduced for 7 days, and stimulated with high K^+^ buffer supplemented with 100 pM BoNT/Ai^PSG^‐At647N for 5 min at 37°C in a 5% CO_2_ atmosphere. The neurons were then fixed and immunostained against endogenous Syt1 and Ezrin, and imaged with a spinning‐disk confocal microscope as described above (with 20× 0.8 NA/Plan‐Apochromat/550 μm WD air objective). The results shown are from *n* = 16 random ROIs of control and Syt1 sgRNA1 KD neurons, from two independent experiments, and the dot plots indicate the Syt1 mean fluorescence intensity (MFI) ± SEM, and BoNT/Ai^PSG^‐At647N MFI ± SEM, both normalized to the average of the control.

Syt1 is a long‐lived protein that is challenging to knock down (Vevea & Chapman, 2020), and we therefore also used an shRNA KD strategy to achieve Syt1 KD in cultured hippocampal neurons (KD for 14 days). For shRNA Syt1 KD, E18 hippocampal neurons (at DIV7) were co‐transfected with EGFP and pRNAi‐mU6‐Green with sh‐lacZ insertion (Biosettia, Cat. #SORT‐A15) or Syt1 shRNA in a 1:1 ratio using Lipofectamine 2000. On DIV21‐22, the conditioning medium was collected from the culture plates and the neurons were stimulated with high K^+^ buffer supplemented with 10 U of AbobotulinumtoxinA for 5 min at 37°C in a 5% CO_2_ atmosphere, then washed three times with low K^+^ buffer, after which the buffer was replaced with the conditioning medium, and the neurons were chased for 30 min or 16 h at 37°C in a 5% CO_2_ atmosphere. Samples were then fixed and processed for immunofluorescence staining and imaged with a spinning‐disk confocal system as described above. The results presented for 30 min AbobotulinumtoxinA treatment are from *n* = 26 (control) and *n* = 27 (Syt1 shRNA) EGFP‐positive neurons and for 16 h AbobotulinumtoxinA treatment are from *n* = 29 (control) and *n* = 30 (Syt1 shRNA) EGFP‐positive neurons, from two independent experiments; the dot plots indicate the average of the fluorescence intensity ± SEM of endogenous Syt1 and cleaved SNAP‐25 from individual EGFP‐positive somas.

### Quantitative real‐time PCR

Rat hippocampal Syt1 sgRNA1 KD and control neurons were collected for RNA isolation. RNA was isolated using an RNeasy mini kit (Qiagen). 1 μg of RNA was reverse transcribed to cDNA using a high‐capacity reverse transcription kit (Applied Biosystems). RT–PCR was carried out using the following primers: rat SV2A forward 5′‐ CCAGATGGGCTCTGCTTACC‐3′ and reverse 5′‐ CTCATCGTGCTTCCCATTCTCTA‐3′, rat Syn1 forward 5′‐AGCCATAGCCATAGTTGCGG‐3′ and reverse 5′‐CGTCATCCTTAAGGGCCTGAT‐3′, rat beta actin forward 5′‐ CCCGCGAGTACAACCTTCTTG‐3′ and reverse 5′‐GTCATCCATGGCGAACTGGTG‐3′. PowerUpnTM SYBRTM Green Master Mix (ThermoFischer Scientific, Cat. #A25741) and the following cycling conditions were used: denaturation at 95°C for 15 s, annealing at 60°C for 30 s, and extension at 72°C for 30 s for 40 cycles. The mRNA levels were normalized to β‐actin mRNA levels and estimation as delta–delta threshold cycle (ΔΔ*C*_T_) was calculated as described previously (Schmittgen & Livak, 2008). All qRT–PCR reactions were carried out in triplicate in each biological replicate.

### Electrophysiology

To study the toxic function of AbobotulinumtoxinA following Syt1 KD, whole‐cell patch‐clamp electrophysiology experiments were performed on E18 hippocampal neurons at DIV21‐22. CRISPRi sgRNA1 KD of Syt1 and 30 min AbobotulinumtoxinA treatment were done as described above. Experiments were performed at room temperature (20–23°C) using an Axopatch 700B amplifier (Molecular Devices) and Axograph software (Axograph Scientific). Cells were placed in a bath and continuously perfused with extracellular solution containing 140 mM NaCl, 5 mM KCl, 2 mM CaCl_2_, 1 mM MgCl_2_, 10 mM HEPES, and 10 mM D‐glucose, adjusted to pH 7.4 with NaOH. Patch pipettes, fabricated from borosilicate glass capillaries (Harvard Apparatus), were pulled using a horizontal puller (Sutter Instruments), with a resistance of 3–6 MΩ, and fire‐polished. The pipettes were filled with an intercellular solution containing 145 mM CsCl, 2 mM CaCl_2_, 2 mM MgCl_2_, 10 mM EGTA, and 2 mM MgATP, adjusted to pH 7.4 with CsOH. Spontaneous miniature postsynaptic currents (mPSCs) were recorded at a holding potential of −70 mV, in the presence of 1 nM tetrodotoxin (bath‐applied; Abcam Cat. #120055) to block action potentials. Signals were filtered at 5 kHz and sampled at 20 kHz. Recordings with a series resistance above 20 MΩ were discarded. Peak amplitudes and frequencies were calculated using Axograph X (Axograph Scientific). Peak amplitudes were detected with a 3:1 signal‐to‐noise ratio as the threshold, and all peaks were manually examined to select well‐separated events. Peak amplitudes calculated by Axograph X for each event were averaged to determine the final values for each cell. Frequency values were calculated by including all postsynaptic events above the 3:1 signal‐to‐noise ratio for a recording period of at least 10 min. The presented results are from *n* = 5 (naïve), *n* = 8 (control; dCas9‐KRAB/TagBFP2), *n* = 12 (control +30 min AbobotulinumtoxinA; dCas9‐KRAB/TagBFP2 following 30 min AbobotulinumtoxinA treatment), *n* = 9 (Syt1 sgRNA1), and *n* = 11 (Syt1 sgRNA1 + 30 min AbobotulinumtoxinA treatment) recordings from two independent experiments. The dot plots indicate the average from individual recordings ± SD.

### HRP cytochemistry and electron microscopy

Electron microscopy (EM) analysis was performed on cultured E18 hippocampal neurons grown on dishes (CellVis, Cat. #D29‐10‐1.5‐N) at 50,000 neurons per 78.54 mm^2^ confluency to ensure mature and functional synaptic connections. On DIV21‐22, the neurons were stimulated with high K^+^ buffer supplemented with 5 μg ml^−1^ BoNT/Ai^wt^‐HRP, BoNT/Ai^PSG^‐HRP BoNT/Ai^SV2^‐HRP and BoNT/Ai^PSG,SV2^‐HRP for 5 min at 37°C in a 5% CO_2_ atmosphere, washed with low K^+^ buffer (8–10 ml) and chased for 10 min at 37°C in a 5% CO_2_ atmosphere. They were then fixed with 2% paraformaldehyde, 1.5% glutaraldehyde (Electron Microscopy Sciences, Cat. #16210) in 0.1 M sodium cacodylate (Sigma‐Aldrich, Cat. #C0250) buffer, pH 7.4, for 20 min at room temperature, washed three times for 3 min with 0.1 M sodium cacodylate buffer, and processed for 3,3′‐diaminobenzidine tetrahydrochloride (DAB; Sigma‐Aldrich Cat. #D5905) and hydrogen peroxidase (H_2_O_2_) cytochemistry using standard protocols. Samples were contrasted with 1% osmium tetroxide and 2% uranyl acetate before dehydration and embedded in LX‐112 or EPON resin using a BioWave tissue processing system (Pelco) as previously described (Joensuu *et al*, 2020). In the cytochemical staining (or peroxidase chemistry), HRP catalyzes an enzymatic reaction with hydrogen peroxidase and DAB substrates, which yields an insoluble reaction product that becomes electron dense during osmium tetroxide treatment. Thin sections (80–90 nm) were cut using an ultramicrotome (Leica Biosystems, UC6FCS), and imaged with a transmission electron microscope (JEOL USA, Inc. model 1101) equipped with a cooled charge‐coupled device camera (Olympus; Morada CCD camera). For ruthenium red (RuR; Sigma 00541) staining, Syt1 sgRNA1 KD neurons were stimulated with high K^+^ buffer alone, or in the presence of 100 pM unlabeled BoNT/Ai^wt^ for 5 min at 37°C in a 5% CO_2_ atmosphere, then washed with low K^+^ buffer (8–10 ml) and chased in low K^+^ buffer for 10 min at 37°C in a 5% CO_2_ atmosphere. Samples were then fixed for 1 h at room temperature in 2.5% GA in 0.1 M sodium cacodylate buffer, pH 7.4 containing 1 mg/ml RuR, washed three times for 10 min with 0.1 M sodium cacodylate buffer pH 7.4, and then incubated in 1% osmium tetroxide diluted in 0.1 M cacodylate buffer pH 7.4 containing 1 mg/ml RuR for 3 h at room temperature. After two 10 min washes with 0.1 M sodium cacodylate buffer pH 7.4, samples were processed embedded in LX‐112 resin using a BioWave tissue processing system (Pelco) as previously described (Joensuu *et al*, 2020).

Images were acquired when HRP precipitate was observed. For the quantification of the percentages (%) of total HRP signal, the localization of the HRP‐tagged holotoxins was quantified manually based on the following criteria: (i) endocytic structures smaller than 80 nm in diameter were assigned as synaptic vesicles, (ii) endocytic structures larger than 80 nm in diameter with round morphology were assigned as endosomes, and structures of similar diameter and concave appearance or hollow center were assigned as early endosomes (all counted together as endosomes), (iii) large endocytic structures with a multivesicular appearance were assigned as multivesicular bodies, (iv) large double‐membrane endocytic structures that contained cellular material were assigned as autophagosomes, as we have described earlier (Harper *et al*, 2011; Wang *et al*, 2015), (v) tubular structures approximately 50–80 nm in diameter, and free of ribosomes, were assigned as tubular structures, and (vi) separate HRP precipitates connected to the extracellular side of the plasma membrane were assigned as plasma membrane staining. Interconnected tubular structures with more than one HRP precipitate were counted as one structure. In total, 376, 308, 295, and 206 structures containing HRP precipitate, or separate HRP precipitates on the plasma membrane, were counted for BoNT/Ai^wt^‐HRP, BoNT/Ai^PSG^‐HRP, BoNT/Ai^SV2^‐HRP and BoNT/Ai^PSG,SV2^‐HRP, respectively. For the CRISPRi experiments, 394 and 464 structures containing HRP precipitate, or separate HRP precipitates on the plasma membrane, were counted for BoNT/Ai^wt^‐HRP in dCas9‐KRAB/TagBFP2 (control) and CRISPRi sgRNA1 Syt1 KD neurons, respectively. The results are presented as average ± SEM. For the quantification of the average sectional area (μm^2^) of the endocytic profiles containing the HRP precipitate of a given holotoxin, individual HRP‐stained profiles were segmented and the area was measured in ImageJ/Fiji (Schindelin *et al*, 2012; Schneider *et al*, 2012; https://imagej.nih.gov/ij/), with the dot plots indicating the average area (μm^2^) of an endocytic profile ± SEM.

### Electron tomography

E18 rat hippocampal neurons were transduced on DIV14 with CRISPRi Syt1 sgRNA1 KD for 7 days and processed for EM as described above. For Syt1 KD electron tomography, EM samples were prepared as above. Approximately 200 nm resin sections were then cut on a Leica Ultracut 6 ultramicrotome, and the grid was subsequently coated with a thin carbon layer. Tomography was completed as described previously (Ariotti *et al*, 2015). In brief, a dual‐axis tilt series spanning ± 60° with 1° increments was acquired on a Tecnai F30 transmission electron microscope (FEI) at 300 kV using a Gatan one‐view camera under the control of Serial EM. Tilt series were reconstructed using weighted back‐projection and patch tracking in IMOD (https://bio3d.colorado.edu/imod/). Segmentation was performed by density thresholding using the Isosurface Render program in IMOD for specific ROIs.

### Mouse phrenic nerve hemidiaphragm assay

To demonstrate that the At647N labeling of BoNT/A holotoxin did not impair its mechanism of action, the potency of wildtype active BoNT/A‐At647N was determined in comparison to nonlabeled wildtype active BoNT/A using the mouse phrenic nerve hemidiaphragm (MPN) assay. The MPN assay was performed using 20–30 g Swiss mice (Janvier SA) as described previously (Bigalke & Rummel, 2015). According to §4 Abs. 3 (sacrificing animals for scientific purposes, German animal protection law [TSchG]), the number of animals sacrificed by trained personnel before dissection of organs was reported yearly to the animal welfare officer of the Central Animal Laboratory and to the local authority, Veterinäramt Hannover. Isolated *N. phrenicus* hemidiaphragm tissue was then treated with nonlabeled BoNT/A or BoNT/A‐At647N, resulting in a characteristic time‐dependent decrease of the contraction amplitude of the indirectly stimulated muscle (Bigalke & Rummel, 2015). Employing the dose–response curve previously established for recombinant BoNT/A (Weisemann *et al*, 2015), catalytically active BoNT/A‐At647N retained high potency (~ 20%) of the nonlabeled BoNT/A. This reduction is acceptable considering the up to one order of magnitude lot‐to‐lot variation of specific toxicity of BoNT/A isolated from *C. botulinum* cultures. We estimate that the impairment to the mechanism of action caused by the At647N labeling of BoNT/Ai^wt^, BoNT/Ai^PSG^, BoNT/Ai^SV2^, and BoNT/Ai^PSG,SV2^ is highly likely to be in a similar range to that of BoNT/A‐At647N, given that all single‐site mutations (receptor‐binding site mutations W1266L/G1141D/G1292R, and LC inactivation E224A/R363A/Y366F) neither remove nor introduce the lysine residues that the NHS‐ester targets.

### uPAINT imaging

Universal point accumulation imaging in nanoscale topography (uPAINT) experiments were performed as described earlier (Giannone *et al*, 2013; Joensuu *et al*, 2016). To image the single‐molecule mobility of BoNT/Ai^wt^‐At647N, BoNT/Ai^PSG^‐At647N, BoNT/Ai^SV2^‐At647N and BoNT/Ai^PSG,SV2^‐At647N holotoxins on the plasma membrane, E18 hippocampal neurons were grown on super‐resolution‐compatible glass‐bottom dishes (Joensuu *et al*, 2017), and on DIV21‐22 the growth medium was replaced with low K^+^ buffer. The neurons were then transferred to the microscope, and stimulated with high K^+^ buffer supplemented with 100 pM holotoxins using a custom‐made perfusion system (Joensuu *et al*, 2017). The single‐molecule mobility of the holotoxins landing on the plasma membrane was then immediately imaged using a Roper Scientific iLas^2^ Ring‐TIRF (total internal reflection fluorescence) microscope with a CFI Apo 100×/1.49 N.A. an oil‐immersion objective (Nikon Instruments), an Evolve 512 Delta EMCCD camera (Photometrics) mounted on a TwinCam LS Image Splitter (Cairn Research), a Perfect Focus System (Nikon), an iLas^2^ double‐laser illuminator (Roper Scientific) for 360° TIRF illumination, and a 642 nm laser (100 mW, Vortran). TetraSpeck microspheres (ThermoFisher Scientific, Cat. #T7279) were used for TIRF angle calibration prior to imaging. Image acquisition was performed using MetaMorph software (Molecular Devices, Version 7.10.2). The uPAINT results for BoNT/Ai^wt^‐At647N, BoNT/Ai^PSG^‐At647N, BoNT/Ai^SV2^‐At647N and BoNT/Ai^PSG,SV2^‐At647N are shown from *n* = 17 acquisitions from randomly chosen ROIs (i.e., number of individual neuronal cultures; one acquisition per culture), originating from five to six independent experiments (i.e., independent embryonic recoveries). The average number of tracks ± SEM per acquisition was 5,800 ± 1,100 for BoNT/Ai^wt^‐At647N, 1800 ± 300 for BoNT/Ai^PSG^‐At647N, 4,200 ± 1,100 for BoNT/Ai^SV2^‐At647N and 2,400 ± 400 for BoNT/Ai^PSG,SV2^‐At647N, with the dot plots indicating the average mobility ± SEM in each acquisition.

To image SV2A and Syt1 mobility on the plasma membrane and to address the receptor mobility changes upon exposure to BoNT/Ai^wt^ or BoNT/Ai^SV2^, single channel uPAINT (for all control experiments without addition of toxin) and dual‐color uPAINT (for all experiments on the effects of toxins on receptor mobility; see further details on the dual‐color uPAINT imaging below) were carried out separately. To image SV2A^wt^‐pHluorin (SV2A^wt^‐pH) and SV2A^T84A^‐pHluorin (SV2A^T84A^‐pH; Zhang *et al*, 2015) single‐molecule mobility on the plasma membrane in the absence of toxins, uPAINT experiments were carried out as described previously (Joensuu *et al*, 2016) and above, with the following exceptions. Neurons were transfected with SV2A^wt^‐pH or SV2A^T84A^‐pH on DIV14. On DIV21‐22, the neurons were stimulated with high K^+^ buffer supplemented with 100 pM anti‐GFP sdAb antibody—FluoTag‐Q At565 nanobodies (At565nb; Synaptic Systems Cat. #N0301‐At565‐S). High K^+^ stimulation increases the extracellular exposure of the SV2 luminal epitope following synaptic vesicle fusion with the plasma membrane (Keller *et al*, 2004; Dong *et al*, 2007, 2008; Fu *et al*, 2009; Peng *et al*, 2011). At565nb specifically binds to the intravesicular pHluorin‐tag of the constructs following synaptic vesicle fusion with the plasma membrane and exposure of the pHluorin‐tag to the extracellular space. The mobility of SV2A^wt^‐pH/At565nb and SV2A^T84A^‐pH/At565nb were then immediately recorded using TIRF imaging. The ROIs for imaging were chosen based on the pHluorin fluorescence signal of the transfected neurons and observed neuronal morphology. The uPAINT high K^+^ stimulation results for SV2A^wt^‐pH/At565nb with and without BoNT/Ai^wt^‐At647N exposure are shown from *n* = 23 acquisitions and *n* = 11 acquisitions for SV2A^wt^‐pH/At565nb with and without BoNT/Ai^SV2^‐At647N exposure from three to five independent experiments. The uPAINT high K^+^ stimulation results for SV2A^T84A^‐pH/At565nb are shown from *n* = 17 acquisitions for both conditions from three independent experiments. The number of acquisitions refers to individual neuronal cultures (one acquisition per culture) and the number of independent experiments refers to independent embryonic recoveries. The average number of tracks ± SEM per acquisition was 1,000 ± 100 for SV2A^wt^‐pH/At565nb (control for SV2A^wt^‐pH/At565nb + BoNT/Ai^wt^‐At647N), 1,000 ± 200 for SV2A^wt^‐pH/At565nb (control for SV2A^wt^‐pH/At565nb + BoNT/Ai^SV2^‐At647N), and 900 ± 200 for SV2A^T84A^‐pH/At565nb (control for SV2A^T84A^‐pH/At565nb + BoNT/Ai^wt^‐At647N), and the dot plots indicate the average mobility ± SEM from each acquisition.

To image Syt1^wt^‐pHluorin (Diril *et al*, 2006; Harper *et al*, 2020; Syt1^wt^‐pH; a kind gift from Prof. Volker Haucke, The Leibniz‐Institute for Molecular Pharmacology), Syt1^K52A^‐pHluorin (Syt1^K52A^‐pH) and Syt1^K326A,K328A^‐pHluorin (Syt1^K326A,K328A^‐pH; Zhang *et al*, 2015) single‐molecule mobility on the plasma membrane in the absence of BoNT/Ai, uPAINT experiments were carried out as described above by transfecting the neurons with the respective constructs. The ROIs for imaging were chosen based on the pHluorin fluorescence signal of the transfected neurons and observed neuronal morphology. The uPAINT high K^+^ stimulation (control) results for Syt1^wt^‐pH/At565nb, Syt1^K52A^‐pH/At565nb, and Syt1^K326A,K328A^‐pH/At565nb are shown from *n* = 14 acquisitions/condition originating from three to four independent experiments. The number of acquisitions refers to individual neuronal cultures (one acquisition per culture) and the number of independent experiments refers to independent embryonic recoveries. The average number of tracks ± SEM per acquisition were 8,900 ± 2,200 for Syt1^wt^‐pH/At565nb (control for Syt1^wt^‐pH/At565nb + BoNT/Ai^wt^‐At647N), 5,900 ± 1,000 for Syt1^K52A^‐pH/At565nb (control for Syt1^K52A^‐pH/At565nb + BoNT/Ai^wt^‐At647N), and 4,900 ± 800 SEM for Syt1^K326A,K328A^‐pH/At565nb (control for Syt1^K326A,K328A^‐pH/At565nb + BoNT/Ai^wt^‐At647N), and the dot plots indicate the average mobility ± SEM in each acquisition.

### Dual‐color uPAINT imaging

The mobilities of SV2A^wt^‐pH/At565nb and SV2A^T84A^‐pH/At565nb following high K^+^ stimulation supplemented with 100 pM BoNT/Ai^wt^ or BoNT/Ai^SV2^ were acquired simultaneously with dual‐color uPAINT imaging, as described earlier (Small *et al*, 2021b), and the receptor mobility was analyzed separately. Briefly, E18 hippocampal neurons were grown on super‐resolution‐compatible glass‐bottom dishes (Joensuu *et al*, 2017) and, on DIV14, were transfected with SV2A^wt^‐pH or SV2A^T84A^‐pH. On DIV21‐22, uPAINT imaging was carried out by stimulating the neurons with high K^+^ buffer supplemented with 100 pM BoNT/Ai^wt^ or 100 pM BoNT/Ai^SV2^, and 100 pM anti‐GFP At565nb, and the mobilities of the At647N‐labeled holotoxin and SV2A^wt^‐pH/At565nb and SV2A^T84A^‐pH/At565nb were simultaneously recorded using TIRF microscopy. We used the imaging setup described above with the following additions. Two Evolve 512 Delta EMCCD cameras (Photometrics) were employed to record simultaneously on two channels, with a TIRF‐quality ultra‐flat quadruple beam splitter (ZT405/488/561/647rpc; Chroma Technology) for distortion‐free reflection of lasers and QUAD emission filter (simultaneous imaging quadruple laser filter set; ZET405/488/561/640 m; Chroma) for dual imaging. TetraSpeck microspheres were used for dual‐camera alignment and TIRF angle calibration prior to imaging. The uPAINT results for SV2A^wt^‐pH/At565nb and SV2A^T84A^‐pH/At565nb following holotoxin treatment are shown from *n* = 11–23 acquisitions per condition from three to five independent experiments. The average number of tracks±SEM per acquisition was 1,100 ± 150 for SV2A^wt^‐pH/At565nb (following BoNT/Ai^wt^‐At647N exposure) and 900 ± 400 SEM for SV2A^T84A^‐pH/At565nb (following BoNT/Ai^wt^‐At647N exposure), and the dot plots indicate the average mobility ± SEM in each acquisition.

Syt1^wt^‐pH/At565nb, Syt1^K52A^‐pH/At565nb, and Syt1^K326A,K328A^‐pH/At565nb mobility data following 100 pM BoNT/Ai^wt^‐At647N‐treatment were acquired with a similar dual‐color uPAINT imaging setup. The number of acquisitions refers to individual neuronal cultures (one acquisition per culture). ROIs were chosen based on the transfected neuron pHluorin fluorescence and neuronal morphology. The average number of tracks ± SEM per acquisition was 7,700 ± 2,900 for Syt1^wt^‐pH/At565nb, 3,500 ± 600 for Syt1^K52A^‐pH/At565nb, and 2,800 ± 500 for Syt1^K326A,K328A^‐pH/At565nb, all following BoNT/Ai^wt^‐At647N exposure, and the dot plots indicate the average mobility ± SEM in each acquisition.

### sdTIM imaging

Subdiffractional tracking of internalized molecules (sdTIM) experiments were performed as previously described (Joensuu *et al*, 2016, 2017). To image the single‐molecule mobility of BoNT/Ai^wt^‐At647N, BoNT/Ai^PSG^‐At647N, BoNT/Ai^SV2^‐At647N or BoNT/Ai^PSG,SV2^‐At647N holotoxins following internalization, E18 hippocampal neurons were grown on super‐resolution‐compatible glass‐bottom dishes, and on DIV21‐22 the growth medium was replaced with low K^+^ buffer. The neurons were then stimulated with high K^+^ buffer supplemented with 1 nM BoNT/Ai^wt^‐At647N, BoNT/Ai^PSG^‐At647N, BoNT/Ai^SV2^‐At647N or BoNT/Ai^PSG,SV2^‐At647N holotoxins for 5 min at 37°C in a 5% CO_2_ atmosphere, washed three times with low K^+^ buffer (8–10 ml) to remove unbound toxins and chased for 10 min at 37°C in a 5% CO_2_ atmosphere to induce activity‐dependent endocytic uptake of the holotoxins. They were then imaged as described above at 50 Hz (16,000 frames by image streaming) and 20 ms exposure, at 37°C in HILO (highly inclined and laminated optical sheet) illumination (Tokunaga *et al*, 2008). TetraSpeck microspheres were used for dual‐camera alignment and HILO angle calibration prior to imaging. sdTIM results are shown from *n* = 17 acquisition per condition from randomly chosen ROIs, originating from five to six independent experiments (number of independent embryonic recoveries). The number of acquisitions refers to individual neuronal cultures (one acquisition per culture). The average number of tracks ± SEM per acquisition was 2,100 ± 700 for BoNT/Ai^wt^‐At647N, 400 ± 50 for BoNT/Ai^PSG^‐At647N, 800 ± 200 for BoNT/Ai^SV2^‐At647N and 500 ± 80 for BoNT/Ai^PSG,SV2^‐At647N, and the dot plots indicate the average mobility ± SEM.

### Single‐molecule tracking

The single‐molecule localization and dynamics were extracted from the 16,000 frame TIRF and HILO acquisitions as described previously (Nair *et al*, 2013; Joensuu *et al*, 2016). BoNT/Ai^wt^‐At647N, BoNT/Ai^PSG^‐At647N, BoNT/Ai^SV2^‐At647N, and BoNT/Ai^PSG,SV2^‐At647N, as well as SV2A^wt^‐pH/At565nb, SV2A^T84A^‐pH/At565nb, Syt1^wt^‐pH/At565nb, Syt1^K52A^‐pH/At565nb, and Syt1^K326A,K328A^‐pH/At565nb, were detected and tracked using a combination of wavelet segmentation (Izeddin *et al*, 2012) and simulated annealing (Racine *et al*, 2006). PALMTracer (Kechkar *et al*, 2013; Nair *et al*, 2013) in Metamorph software (MetaMorph Microscopy Automation and Image Analysis Software, v7.7.8; Molecular Devices) was used to obtain the mean square displacement (MSD) and diffusion coefficient (D; μm^2^ s^−1^) values. Tracks shorter than eight frames were excluded from the analysis to minimize nonspecific background. Cross‐correlation drift correction of the uPAINT data was done using the SharpViSu tool (Andronov *et al*, 2016; https://github.com/andronovl/SharpViSu) in MATLAB 2017b (https://au.mathworks.com/matlabcentral/answers/498405‐matlab‐2017b‐download). The Log_10_D immobile and mobile fraction distributions were calculated as previously described (Constals *et al*, 2015), setting the displacement threshold to 0.03 μm^2^ s^−1^ (*i.e*., dotted line in the graph Log_10_D = −1.45 when [D] = μm^2^ s^−1^ as described previously; Constals *et al*, 2015; Joensuu *et al*, 2016). The mobile to immobile (M/IMM) ratio was determined based on the frequency distribution of the diffusion coefficients (Log_10_D) of immobile (Log_10_D ≤ −1.45) and mobile (Log_10_D > −1.45) molecules (the immobile fraction of molecules represents BoNT/A^wt^‐At647N molecules for which the displacement within 4 frames was below the spatial detection limit of our methods, 106 nm). The area under the MSD curve (AUC) was calculated in Prism 9 for macOS version 9.1.1 (GraphPad Prism 9 for macOS; https://www.graphpad.com/scientific‐software/prism/). The super‐resolved image color‐coding was done as previously described (Joensuu *et al*, 2017) using ImageJ/Fiji (2.0.0‐rc‐43/1.50e; National Institutes of Health), with each colored pixel in the average intensity maps indicating the localization of an individual molecule (bar: 8 to 0, high to low density), the color‐coded pixels in the average diffusion coefficient map presenting an average value for each single‐molecule track at the site of localization (bar: Log_10_ 1 to −5, high to low mobility), and the color‐coding of the track maps representing the detection time point (bar: 0–16,000 frame acquisition) during acquisition.

### *In situ* proximity ligation assay

Hippocampal neurons from E18 rats were seeded on glass‐bottom dishes (CellVis, Cat. #D29‐10‐1.5‐N) at 50,000 neurons per 78.54 mm^2^ confluency to ensure mature and functional synaptic connections. On DIV21‐22, neurons were left untreated (naïve control), stimulated for 5 min in high K^+^ buffer (vehicle control), or stimulated for 5 min in high K^+^ buffer supplemented with 100 pM BoNT/Ai^wt^‐At647N, BoNT/Ai^PSG^‐At647N, or BoNT/Ai^SV2^‐At647N. They were then fixed with 4% paraformaldehyde in PBS for 20 min at room temperature, washed three times with PBS, permeabilized with 0.1% Triton X‐100 for 4 min, washed three times with PBS, and processed for the *in situ* proximity ligation assay (PLA) according to the manufacturer's instructions (DuoLink, Merck, Cat. #DUO92101). Every experiment was performed with a pair of primary antibodies against endogenous Syt1 (Anti‐Syt1, Abcam, Cat. #ab13259; 1:200 dilution) and SV2C (Anti‐SV2C, Synaptic Systems, Cat. #119203; 1:200 dilution). Neurons were mounted in Duolink *in situ* mounting medium containing DAPI. Optical sections of the fluorescence signal of PLA dots and DAPI, spanning entire neurons, were acquired from 10 random ROIs in each sample. Imaging was done using a spinning‐disk Marianas 3I, Inc confocal system as described earlier, with the exception of using a 63× 1.4 NA/Plan‐Apochromat/180 μm WD objective. For image analysis, a 200 × 200 pixel area was selected manually around each nucleus (based on DAPI labeling, with overlapping nuclei being excluded) and the midsection of the nucleus was determined based on the peak fluorescence intensity of DAPI using the Plot z‐axis Profile‐function in ImageJ/Fiji. To quantify Syt1‐SV2C interactions at the synaptic connections in the somatodendritic area, the site of synaptic contacts, and to avoid the uniform nonspecific PLA signal observed on the glass bottom of the dish, a maximum intensity projection of the PLA signal was then obtained from ± 10 optical slices (z‐step 0.2 μm) offsetting from the nuclear midsection of each neuron. Automated image analysis to identify and quantify the number of PLA dots (reflecting the detection of a Syt1‐SV2C protein–protein interaction) using ImageJ/Fiji (Version 2.1.0/1.53c) was then conducted as described previously (Lopez‐Cano *et al*, 2019). The results presented are from *n* = 25 randomly chosen 200 × 200 pixel ROIs encompassing the DAPI staining, from two independent experiments for each condition, and the dot plots indicate the number of PLA dots per nucleus ± SEM (with neurons originating from embryos of different dams).

### Automated image segmentation and fluorescence intensity analysis

The quantification of Syt1 and cleaved SNAP‐25 fluorescence following CRISPRi (Fig 5A–C) was done using CellProfiler 3 (McQuin *et al*, 2018) (https://cellprofiler.org/) and ImageJ/Fiji. Using ImageJ/Fiji, confocal stacks of the AbobotulinumtoxinA‐treated and immunostained hippocampal neurons were Z‐projected using the sum of fluorescence. The 2D Z‐projections were then used in CellProfiler 3 to determine the area positive for TagBFP2. TagBFP2 was introduced in the lentivirus vector expression cassette to identify (can be excited with ultraviolet light for imaging) and segment neurons that had received the dCas9‐KRAB (control, nontargeting sgRNA) or the respective Syt1 sgRNAs. First, the brightest TagBFP2 fluorescent spots were identified using the Identify Primary Object tool of CellProfiler 3 and the otzu algorithm (green areas highlighted in Appendix Fig S1A). Next, these primary spots were expanded following the TagBFP2 fluorescence signal until the whole TagBFP2 ROI of each image was segmented (blue areas highlighted in Appendix Fig S1A). The mean fluorescence intensity (MFI, in arbitrary units) of endogenous Syt1 and cleaved SNAP‐25 was then measured from the segmented ROI. The mean background fluorescence intensity was determined manually from 15 areas outside the cells in each image. This value was subtracted from the MFI value, and the presented graphs show MFI ± SEM.

The MFI quantification for Syt1 shRNA KD (Appendix Fig S1B–H) was performed similar to the approach described above for CRISPRi by first Z‐projecting the sum fluorescence of the 3D‐stacks acquired with a spinning‐disk confocal microscope as above. The 2D Z‐projections were then used in CellProfiler 3 to segment the area positive for EGFP (a marker to identify neurons that had received the co‐transfected EGFP with the respective shRNA). The values of threshold fluorescence intensity were chosen in CellProfiler 3 so that only the brightest signal of the EGFP‐positive cells was automatically detected in all images. These bright areas corresponded to the neuronal soma (boxed area shown in higher magnification on right). The MFIs of Syt1 and cleaved SNAP‐25 were then quantified in these selected areas. SNAP‐25 localization in differentiated neurons has been shown to be plasma membrane‐associated in axonal compartments and not restricted to synaptic junctions, as well as localizing in tubulo‐vesicular structures in the cytoplasm of the soma and the axon, but rarely in the smaller dendrites (Tao‐Cheng *et al*, 2000). In the shRNA experiments, the Syt1 and SNAP‐25/A MFI were quantified in the soma compartment, which is the site for many synaptic connections, similar to quantifications previously done in motorneurons (Antonucci *et al*, 2008), due to low neuronal transfection efficiency. By contrast, in the CRISPRi experiments, the MFI was quantified in whole neurons.

For pLenti6.3‐Syt1^wt^‐pH and pLenti6.3‐Syt1^K52A^‐pH rescue of Syt1 sgRNA1 CRISPRi, 3D images acquired by spinning‐disk confocal microscopy were Z‐projected into 2D images using the “Z‐project sum of fluorescence intensities” function of the ImageJ. CellProfiler 3 was used to automatically identify TagBFP2‐positive cells using a manually determined threshold of fluorescence estimated as > 2× the median background fluorescence outside the cells. The identified TagBFP2‐positive areas corresponded to the soma of each neuron (the less bright dendrites and axons where not selected by the threshold of fluorescence). Within these TagBFP2‐positive areas, the software measured the MFI of Syt1^wt^‐pH, Syt1 (immunofluorescence of Syt1), and SNAP‐25/A (immunofluorescence staining of cleaved SNAP‐25). Using the same software, from each 2D image, the median fluorescence of fifteen 20 × 20 pixel ROIs outlining individual neurons was manually determined for each of the four fluorescence channels, and these intensity values were subtracted from all the final measurements of each respective channel. The pLenti6.3‐Syt1^wt^‐pH data are reported from *n* = 3 random ROIs of both Syt1 sgRNA and control conditions (one independent experiment), and the dot plots indicate the MFI ± SEM of endogenous Syt1 and cleaved SNAP‐25. The regression graphs (± 95% confidence intervals) are from representative Z‐projected stacks of Syt1 sgRNA1 CRISPRi rescued with pLenti6.3‐Syt1^wt^‐pH (Fig 1G), nontreated control neurons with pLenti6.3‐Syt1^wt^‐pH (Fig 1J) and Syt1 sgRNA1 CRISPRi rescued with pLenti6.3‐Syt1^K52A^‐pH (Appendix Fig S7L–N). Graphs were made in Prism 9, and the R^2^ for the regression analysis was determined in Excel (Microsoft Excel for Mac, version 16.55).

### Nanoscale spatiotemporal indexing clustering (NASTIC) analysis

NASTIC (Wallis *et al*, 2021) was used to perform nanocluster analysis on the single‐molecule localization data obtained with uPAINT. NASTIC uses the R‐tree spatial indexing algorithm (Gutmann, 1984) to determine the overlap of the bounding boxes of individual molecular trajectories, as a measure of their potential membership in nanoclusters. The spatial extent of a cluster represents the convex hull encompassing the individual detections of all overlapping trajectories in the cluster. Extending the spatial indexing into the time dimension allowed us to expand the spatial nanoclustering into spatiotemporal clustering, such that trajectory bounding boxes must overlap in both space and time to be considered a cluster. This approach enabled us to determine clustering metrics such as percentage of clustered tracks and co‐clustered tracks, as well as identifying spatial “hotspots” of repeated cluster formation over the acquisition period. NASTIC employs several parameters: the radius factor (*r*) represents the multiplication of the radius of the ideal circularized extent of each trajectory used to construct the regular 2D spatial bounding region, and the time window (*t*) represents the temporal “thickness” of the resulting 3D bounding box. For these analyses, the default values of *r* = 1.2 and *t* = 20 ms were used. Overlapping trajectories within ± 10 s in the same ROIs were counted as clusters. Clusters with a radius of > 150 nm were excluded from subsequent metrics. For visualization, identified clusters were colored according to the average detection time in the analysis, such that blue/red/green clusters represent those from early/middle/later in the analysis, respectively. Hotspots of repeated cluster formation are highlighted in white. 2D kernel density estimations were performed using individual molecular detections and were colored qualitatively such that higher density regions were represented with higher color temperature. Instantaneous diffusion coefficient plots of trajectories are colored such that smaller values are represented by higher color temperature (color bar range −5 to 1, log_10_D). Outliers were removed using the ROUT method with Q = 1% in Prism 9. *n* = 11–19 neurons/condition from three to five independent experiments from the respective single‐molecule experiments.

### Structural modeling of the SV2A‐Syt1‐GT1b‐BoNT/A complex

A structural model of the BoNT/A holotoxin in complex with tripartite GT1b‐Syt1‐SV2A was compiled step‐wise beginning with the full‐length BoNT/A crystal structure (PDB 2NYY; Garcia‐Rodriguez *et al*, 2007) and full‐length models of human SV2A and Syt1 from the AlphaFold2 database (https://alphafold.ebi.ac.uk; Jumper *et al*, 2021; Tunyasuvunakool *et al*, 2021; https://github.com/deepmind/alphafold). The full‐length SV2A and BoNT/A proteins were aligned with the co‐crystal structure of the SV2C extracellular domain in complex with the 50 kDa BoNT/A‐H_C_ fragment (PDB 4JRA; Benoit *et al*, 2014). The full‐length Syt1 was then aligned to both the co‐crystal structure of the GT1b ganglioside bound to the 50 kDa BoNT/A‐H_C_ fragment (Stenmark *et al*, 2008), and the co‐crystal structure of the Syt1 C2B domain bound to the peptide of SV2A phosphorylated at Thr84 (Zhang *et al*, 2015). The approximate alignment of Syt1 bound to GT1b was made based on the previously published molecular dynamics models of this complex (Flores *et al*, 2019). Alignments were performed in Pymol (Schrodinger), with adjustments to flexible regions in SV2A and Syt1 done manually to allow for their docking to bound partners in a physically reasonable way. Finally, bond lengths and angles were optimized in Coot (Emsley & Cowtan, 2004; https://www2.mrc‐lmb.cam.ac.uk/personal/pemsley/coot/). Structural images were made in Pymol (Schrodinger, USA; https://pymol.org/2/). It is worth noting that AlphaFold2 predictions are not always as accurate as more traditional experimental methods and predict one stable conformation per protein, ignoring any dynamic changes in the protein structures. Therefore, the presented tripartite GT1b‐Syt1‐SV2 complex for selective binding and endocytic targeting of BoNT/A should only be regarded as a predictive model and will have to be confirmed by other methods.

### Quantification and statistical analysis

Statistical tests were conducted in Prism 9 for macOS version 9.1.1. The normality of the data was tested with Kolmogorov–Smirnov tests unless otherwise stated, and nonparametric tests and parametric tests were used to compare two independent groups (Mann–Whitney test or *t* test) and multiple groups (Kruskal–Wallis test or ordinary one‐way ANOVA multiple comparison test). All the presented super‐resolution results in dot plots are from separate technical replications (*i.e*., from separate cultures) from the indicated number of independent experiments. Dot plots indicate average ± SEM from individual acquisitions or ROIs unless otherwise stated. Additional details on sample sizes for each experiment can be found in the description of each experiment in the Methods section and respective Figure legends.

### Image adjustments for figures and preparation of manuscript figures

Brightness and contrast of acquired images were adjusted in ImageJ/Fiji and Adobe Photoshop 22.4.3 release (Adobe). Pseudocoloring of multichannel acquisition was done in ImageJ/Fiji. Figures were made in Adobe Illustrator 25.4.1 release (Adobe).

## Author contributions

**Frédéric A Meunier:** Conceptualization; resources; supervision; funding acquisition; project administration; writing – review and editing. **Merja Joensuu:** Conceptualization; data curation; formal analysis; supervision; validation; investigation; visualization; methodology; writing – original draft; project administration; writing – review and editing. **Parnayan Syed:** Formal analysis; investigation; writing – review and editing. **Saber H Saber:** Formal analysis; investigation; writing – review and editing. **Vanessa Lanoue:** Formal analysis; investigation; visualization; writing – review and editing. **Tristan P Wallis:** Software; formal analysis; investigation; writing – review and editing. **James Rae:** Formal analysis; investigation; writing – review and editing. **Ailisa Blum:** Formal analysis; investigation; visualization; writing – review and editing. **Rachel S Gormal:** Resources; validation; writing – review and editing. **Christopher Small:** Validation; writing – review and editing. **Shanley Sanders:** Validation; writing – review and editing. **Anmin Jiang:** Validation; writing – review and editing. **Stefan Mahrhold:** Formal analysis; validation; investigation; writing – review and editing. **Nadja Krez:** Formal analysis; validation; investigation; writing – review and editing. **Michael A Cousin:** Resources; writing – review and editing. **Ruby Cooper‐White:** Resources; writing – review and editing. **Justin J Cooper‐White:** Resources; writing – review and editing. **Brett M Collins:** Formal analysis; investigation; writing – review and editing. **Robert G Parton:** Formal analysis; investigation; writing – review and editing. **Giuseppe Balistreri:** Resources; formal analysis; validation; investigation; visualization; methodology; writing – review and editing. **Andreas Rummel:** Conceptualization; resources; formal analysis; supervision; validation; investigation; visualization; methodology; writing – review and editing.

## Disclosure and competing interests statement

The authors declare that they have no conflict of interest.

## Supporting information



Appendix S1

Table EV1

Table EV2

Table EV3

Movie EV1

Movie EV2

Movie EV3

Movie EV4

Movie EV5

## Data Availability

Plasmids generated in this study have been deposited to Addgene (see Table EV3). This study did not generate new unique reagents. Plasmids encoding BoNT/A and mutants are excluded from deposition for biosecurity reasons. All original data created in this study and shown in Figs 1, 2, 3, 4, 5 will be uploaded to The University of Queensland RDM (https://rdm.uq.edu.au/files/3d68b6a0-f3bb-11ed-93ec-2b6f3205d72a). Any additional information required to reanalyze the data reported in this paper is available from the lead authors upon request.
