# Supplementary material for: Presynaptic targeting of botulinum neurotoxin type A requires a tripartite PSG‐Syt1‐SV2 plasma membrane nanocluster for synaptic vesicle entry
Source: EMBO J. 2023 May 25;42(13):e112095. doi: 10.15252/embj.2022112095 (PMC10308369; doi:10.15252/embj.2022112095)
Supplement: Supplementary file 1 — Appendix S1 [file EMBJ-42-e112095-s006.pdf]

Table of Content

- Appendix Figure S1 and figure legend, Page 2
- Appendix Figure S2 and figure legend, Page 3
- Appendix Figure S3 and figure legend, Page 4
- Appendix Figure S4, Page 5
- Appendix Figure S4 legend, Page 6
- Appendix Figure S5 and figure legend, Page 7
- Appendix Figure S6 and figure legend, Page 8
- Appendix Figure S7, Page 9
- Appendix Figure S7 legend, Page 10
- Appendix Figure S8, Page 11
- Appendix Figure S8 legend, Page 12

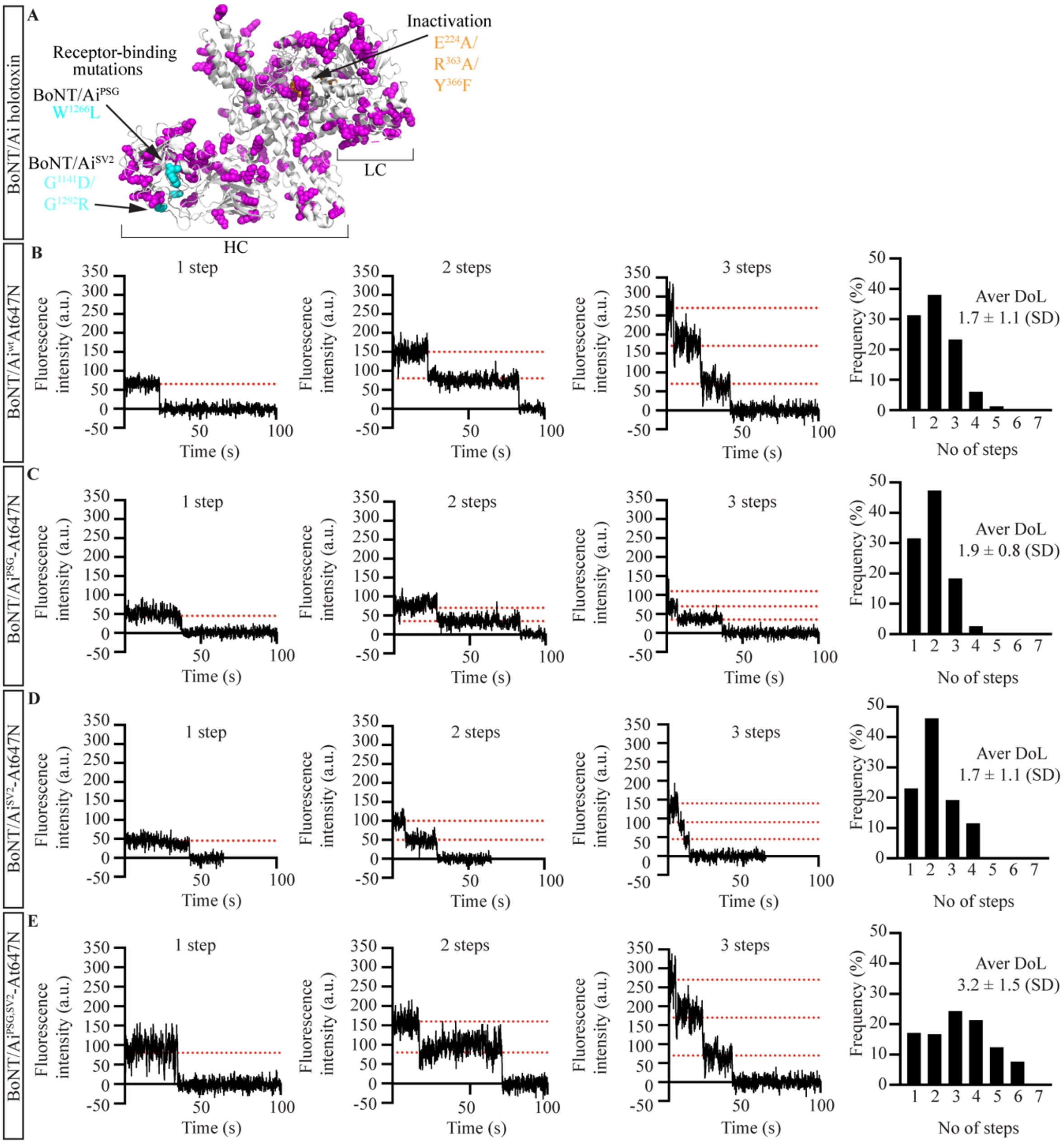

Appendix Figure S 1. Quantification of the degree of At647N-labelling of BoNT/Ai holotoxins
(A) Ribbon structure of BoNT/A holotoxins with lysine residues (magenta), light chain (LC) mutations (orange), and heavy chain (HC) mutations (cyan) indicated.
(B-E) For single-molecule imaging, BoNT/Ai holotoxins were labelled with Atto647N fluorophores, and the number of fluorescence emission steps of the labelled toxins were quantified using a flow-chamber assay. Representative single-molecule fluorescence emission traces of (B) BoNT/Ai^{wt}-At647N, (C) BoNT/Ai^{PSG}-At647N, (D) BoNT/Ai^{SV2}-At647N, and (E) BoNT/Ai^{PSG,SV2}-At647N recorded on a custom-made flow-chamber glass-bottom. The frequency (%) distribution of emission step numbers is shown in the bar graphs on right with the respective average degree of labelling (DoL) for each toxin.
Data information: Results are shown as average ± standard deviation (SD), (B) n = 163 BoNT/Ai^{wt}-At647N molecules, (C) n = 38 BoNT/Ai^{PSG}-At647N molecules, (D) n = 78 BoNT/Ai^{SV2}-At647N molecules, and (E) n = 234 BoNT/Ai^{PSG,SV2}-At647N molecules, all from one experiment/condition.

Degree of labelling (absorbance)

A

No	Sample	A_{280}	A_{647}	Aver DoL
1	BoNT/Ai^{wt}-At647N	0.400	0.465	1.68
2	BoNT/Ai^{wt}-At647N	0.360	0.454	
3	BoNT/Ai^{wt}-At647N	0.320	0.415	
4	BoNT/Ai^{wt}-At647N	0.300	0.415	

No	Sample	A_{280}	A_{647}	Aver DoL
1	BoNT/Ai^{PSG}-At647N	0.280	0.446	1.83
2	BoNT/Ai^{PSG}-At647N	0.330	0.420	
3	BoNT/Ai^{PSG}-At647N	0.280	0.397	

No	Sample	A_{280}	A_{647}	Aver DoL
1	BoNT/Ai^{SV2}-At647N	0.240	0.388	2.17
2	BoNT/Ai^{SV2}-At647N	0.260	0.374	
3	BoNT/Ai^{SV2}-At647N	0.220	0.396	

No	Sample	A_{280}	A_{647}	Aver DoL
1	BoNT/Ai^{PSG,SV2}-At647N	0.450	1.403	3.50
2	BoNT/Ai^{PSG,SV2}-At647N	0.460	1.085	
3	BoNT/Ai^{PSG,SV2}-At647N	0.480	1.088	

Mouse phrenic hemidiaphragm assay

B **C**

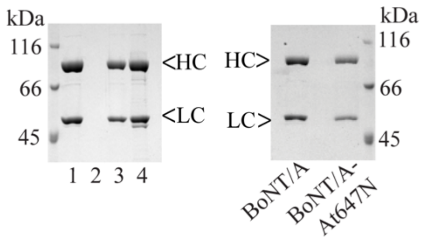

D

Absorbance

No	A_{280}	A_{647}	Aver. DoL
1	0.692	0.791	1.46
2	0.719	0.888	
3	0.825	0.764	

E

Sample	Bath conc. [pM]	$t_{1/2}$ [min]	Mean $t_{1/2}$ [min]	SD [min]	CV	n
BoNT/A	6	66	64.8	4.11	6.35%	4
		61				
		62				
		70				
BoNT/A-At647N	6	95	89.7	8.29	9.24%	6
		81				
		88				
		82				
		103				
		89				

Appendix Figure S 2. At647N labelling of BoNT/A and mouse hemidiaphragm assay

(A) Absorption spectroscopy recordings of BoNT/Ai^{wt}-At647N, BoNT/Ai^{PSG}-At647N, BoNT/Ai^{SV2}-At647N, and BoNT/Ai^{PSG,SV2}-At647N. Repeated measurements of A_{280} and A_{647} are shown in the table together with calculated average degree of labelling (DoL) for each toxin.

(B) SDS-PAGE (10%) analysis of BoNT/A produced recombinantly in *E. coli*, yielding a solution of 2.5 µM (0.37 mg mL^{-1}) in PBS buffer (4.5 µL loaded in lane 1). To further concentrate the toxin, 10 nmol of BoNT/A was ultrafiltrated (MWCO 30 kDa), yielding a solution of 11.5 µM (1.7 mg mL^{-1}; lane 2: flow through of the ultrafiltration, and 1 µL of 1.4 ml BoNT/A solution after ultrafiltration was loaded in lane 3). Lane 4 shows 7.5 µL of the protein pellet after ultrafiltration dissolved in 140 µL (1/10) in PBS. Heavy chain HC, light chain LC.

(C) SDS-PAGE (10%) of BoNT/A (lane 1) and BoNT/A-At647N (1 µL loaded; lane 2).

(D) Quantification of the DoL of BoNT/A-At647N using absorption spectroscopy. Repeated measurements at A_{280} and A_{647} are shown in the table, together with the calculated average DoL.

(E) Functional analysis of BoNT/A and BoNT/A-At647N in the mouse hemidiaphragm assay to test the impact of the At647N labelling on BoNT/A toxicity. Employing the dose-response curve previously established for recombinant BoNT/A, the potency of BoNT/A-At647N was reduced to ~20% compared to non-labelled BoNT/A.

Data information: Results are shown as standard deviation (SD). (A, D) 3 technical replicas from one experiment, (E) n=4-6 independent biological replicates.

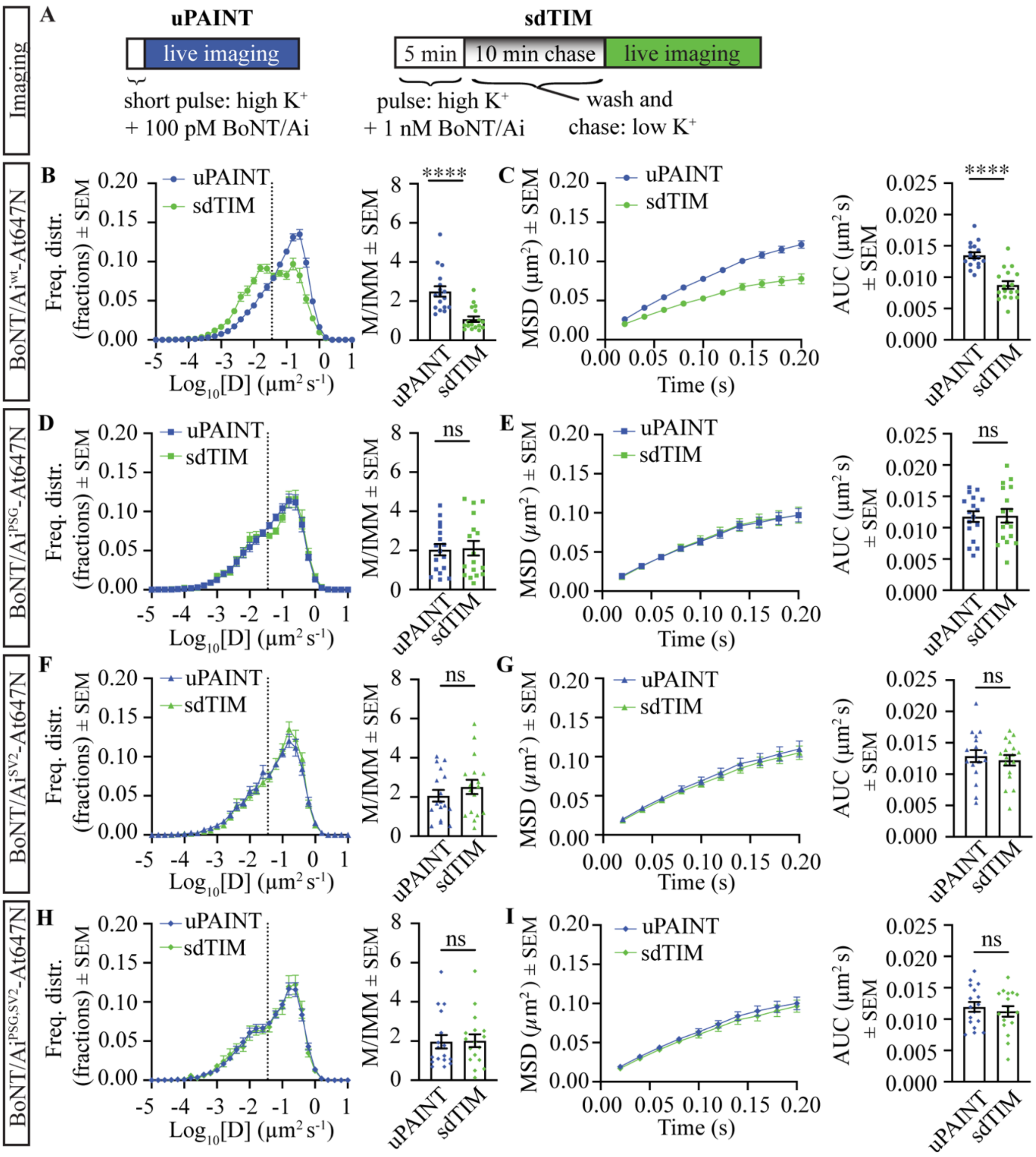

Appendix Figure S 3. Single-molecule imaging of BoNT/Ai holotoxins in hippocampal neurons

(A) Schematic illustrations of uPAINT and sdTIM super-resolution techniques to image the single-molecule mobility of At647N-labelled BoNT/Ai holotoxins on the presynaptic plasma membrane (uPAINT) and following endocytosis (sdTIM).

(B-I) Single-molecule uPAINT and sdTIM mobility quantification of (B, C) BoNT/Ai^{wt}-At647N, (D, E) BoNT/Ai^{PSG}-At647N, (F, G) BoNT/Ai^{SV2}-At647N and (H, I) BoNT/Ai^{PSG,SV2}-At647N indicated as the frequency distribution (fractions) of the Log$_{10}$ diffusion coefficient (threshold for mobile and immobile fractions indicated with dashed line at -1.45), mobile-to-immobile (M/IMM) ratio, mean square displacement (MSD) and area under the MSD curve (AUC).

Data information: Results are shown as average ± standard error of the mean (±SEM). Dots in scatter plots indicate averages from individual acquisitions. N = 17 technical replicates/condition from 5-6 independent experiments. Statistical significance was assessed using non-parametric Mann-Whitney U test (B, D, F, H), and parametric unpaired t test (C, E, G, I). ****$p<0.0001$, and ns – nonsignificant.

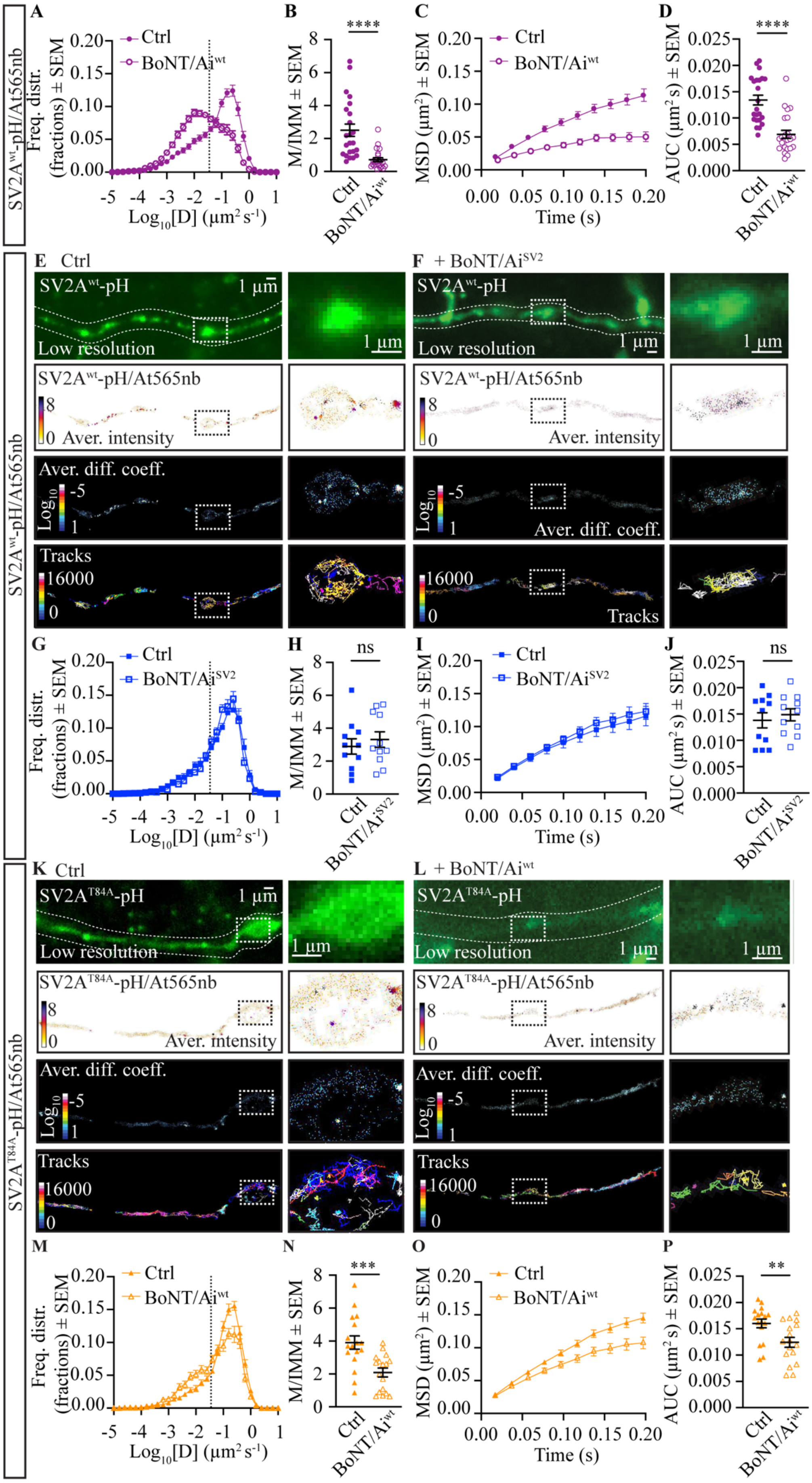

A
B
C
D
SV2A^wt-pH/At565nb
Freq. distr. (fractions) ± SEM
Ctrl
BoNT/Ai^wt
Log10[D] (μm² s⁻¹)
M/IMM ± SEM
****
MSD (μm²) ± SEM
Time (s)
AUC (μm² s) ± SEM
E Ctrl
F + BoNT/Ai^SV2
SV2A^wt-pH
1 μm
Low resolution
SV2A^wt-pH/At565nb
Aver. intensity
Aver. diff. coeff.
Log10
Tracks
16000
G
H
I
J
BoNT/Ai^SV2
ns
K Ctrl
L + BoNT/Ai^wt
SV2A^T84A-pH
SV2A^T84A-pH/At565nb
SV2A^T84A-pH/At565nb
M
N
O
P
***
**

Appendix Figure S 4. BoNT/A-induced SV2A immobilization on the neuronal plasma membrane requires Syt1

(A-D) Single-molecule uPAINT mobility of $SV2A^{wt}$-pH/At565nb in hippocampal neurons shown as (A) The frequency distribution of the diffusion coefficient, (B) mobile-to-immobile (M/IMM) ratio, (C) mean square displacement (MSD) and (D) area under the MSD curve (AUC) in the absence (high K+; Ctrl) and presence of 100 pM $BoNT/Ai^{wt}$-At647N (toxin diluted in high K^+).

(E, F) Representative low-resolution images of a hippocampal neuron expressing $SV2A^{wt}$-pH (green) and the corresponding super-resolved uPAINT average intensity (bar: high 8 to low 0 density), average diffusion coefficient (bar: $Log_{10}D$ high 1 to low -5 mobility) and trajectory maps (bar: 0-16,000 frame acquisition) in (E) control conditions (high K^+; Ctrl) and (F) following treatment with 100 pM $BoNT/Ai^{SV2}$.

(G-J) Single-molecule uPAINT mobility of $SV2A^{wt}$-pH/At565nb in hippocampal neurons shown as indicated in the absence (high K^+; Ctrl) and presence of 100 pM $BoNT/Ai^{SV2}$ (toxin diluted in high K^+).

(K, L) Representative low-resolution images of a hippocampal neuron expressing $SV2A^{T84A}$-pH (green) and the corresponding super-resolved uPAINT maps as indicated in (K) control conditions (high K^+; Ctrl) and (L) following treatment with 100 pM $BoNT/A^{wt}$.

(M-P) Single-molecule uPAINT mobility of $SV2A^{T84A}$-pH/At565nb in hippocampal neurons as indicated in the absence (high K^+; Ctrl) and presence of 100 pM $BoNT/Ai^{wt}$ (toxin diluted in high K^+).

Data information: Results are shown as average ± standard error of the mean (±SEM). Scatter plots indicate averages from individual acquisitions. (A-D, G-J, M-P) n=11-23 /condition from 3-5 independent experiments. Statistical significance was assessed using non-parametric Mann-Whitney U test (B, H, N), parametric unpaired t test (D, J, P). $^{**}p<0.01$, $^{***}p<0.001$, $^{****}p<0.0001$, and ns – nonsignificant.

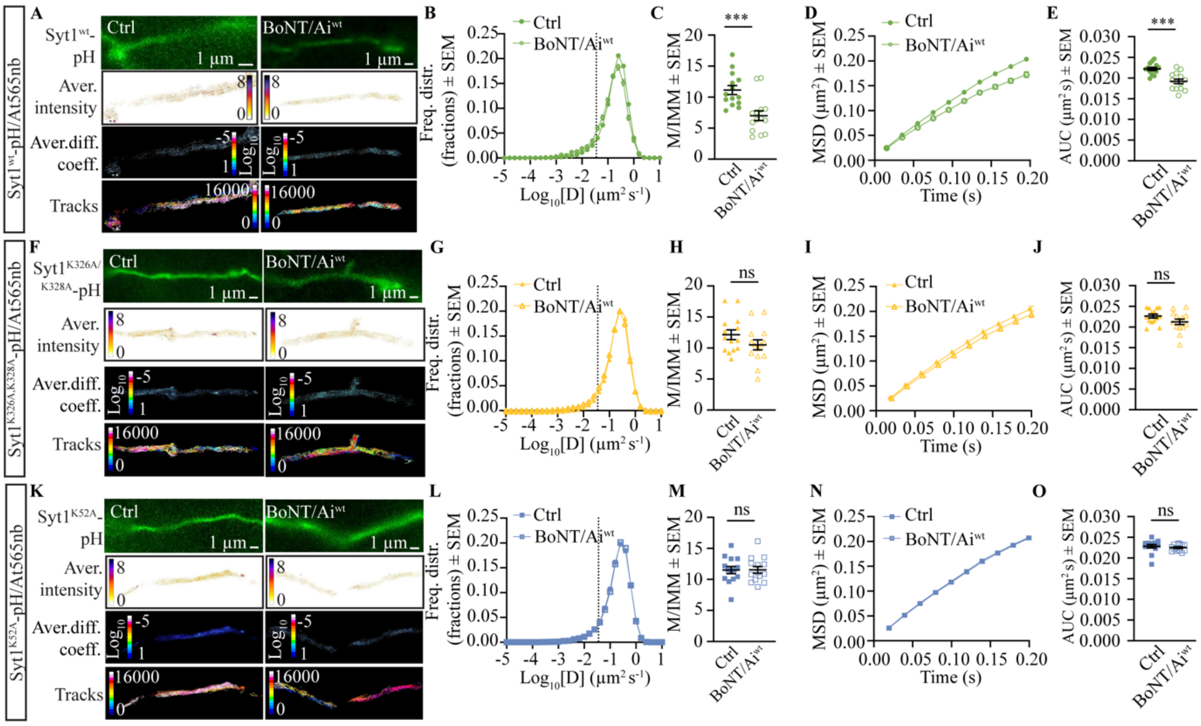

Appendix Figure S 5. BoNT/A-induced Syt1 immobilization at the neuronal plasma membrane requires SV2

(A) Representative low-resolution images of a hippocampal neuron expressing Syt1^{wt}-pH (green) and corresponding super-resolved uPAINT average intensity (bar: high 8 to low 0 density), average diffusion coefficient (bar: $Log_{10}D$ high 1 to low -5 mobility) and trajectory maps (bar: 0-16,000 frame acquisition) in control conditions (high K$^+$; Ctrl) and following treatment with 100 pM BoNT/Ai^{wt}.

(B-E) Single-molecule uPAINT mobility of Syt1^{wt}-pH/At565nb in hippocampal neurons shown as (B) The frequency distribution of the diffusion coefficient, (C) mobile-to-immobile (M/IMM) ratio, (D) mean square displacement (MSD) and (E) area under the MSD curve (AUC) in the absence (high K$^+$; Ctrl) and presence of 100 pM BoNT/Ai^{wt}-At647N (toxin diluted in high K$^+$).

(F) Representative low-resolution images of a hippocampal neuron expressing Syt1$^{K326A/K328A}$-pH (green) and corresponding super-resolved uPAINT maps as indicated in control conditions (high K$^+$; Ctrl) and following treatment with 100 pM BoNT/A^{wt}.

(G-J) Single-molecule uPAINT mobility of Syt1$^{K326A/K328A}$-pH/At565nb in hippocampal neurons as indicated in the absence (high K$^+$; Ctrl) and presence of 100 pM BoNT/Ai^{wt} (toxin diluted in high K$^+$).

(K) Representative low-resolution images of a hippocampal neuron expressing Syt1^{K52A}-pH (green) and corresponding super-resolved uPAINT maps as indicated in control conditions (high K$^+$; Ctrl) and following treatment with 100 pM BoNT/A^{wt}.

(L-O) Single-molecule uPAINT mobility of Syt1^{K52A}-pH/At565nb in hippocampal neurons as indicated in the absence (high K$^+$; Ctrl) and presence of 100 pM BoNT/Ai^{wt} (toxin diluted in high K$^+$).

Data information: Results are shown as average ± standard error of the mean (±SEM). Scatter plots indicate averages from individual acquisitions. (B-E, G-J, L-O) n=11-19 /condition from 3-5 independent experiments. Statistical significance was assessed using non-parametric Mann-Whitney U test (C, H, M), parametric unpaired t test (E, J, O). ***p<0.001, and ns – nonsignificant.

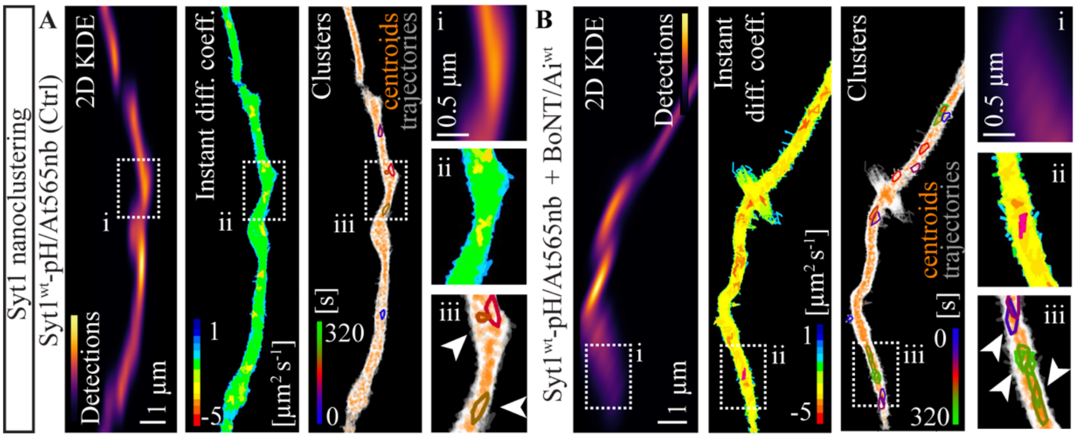

Appendix Figure S 6. BoNT/A-binding to the neuronal plasma membrane leads to nanoclustering of Syt1

Representative images of NASTIC nanocluster analysis of Syt1^{wt}-pH/At565nb in **(A)** the control condition (high K^+; Ctrl) and **(B)** following high K^+ stimulation in the presence of 100 pM BoNT/Ai^{wt}-At647N, with 2D KDE (high density in red), instantaneous Log_{10} diffusion coefficient map (low mobility in red), and nanoclusters with their respective trajectories (white) and centroids (orange) shown. Boxed areas are magnified on the right, with arrowheads indicating nanoclusters.

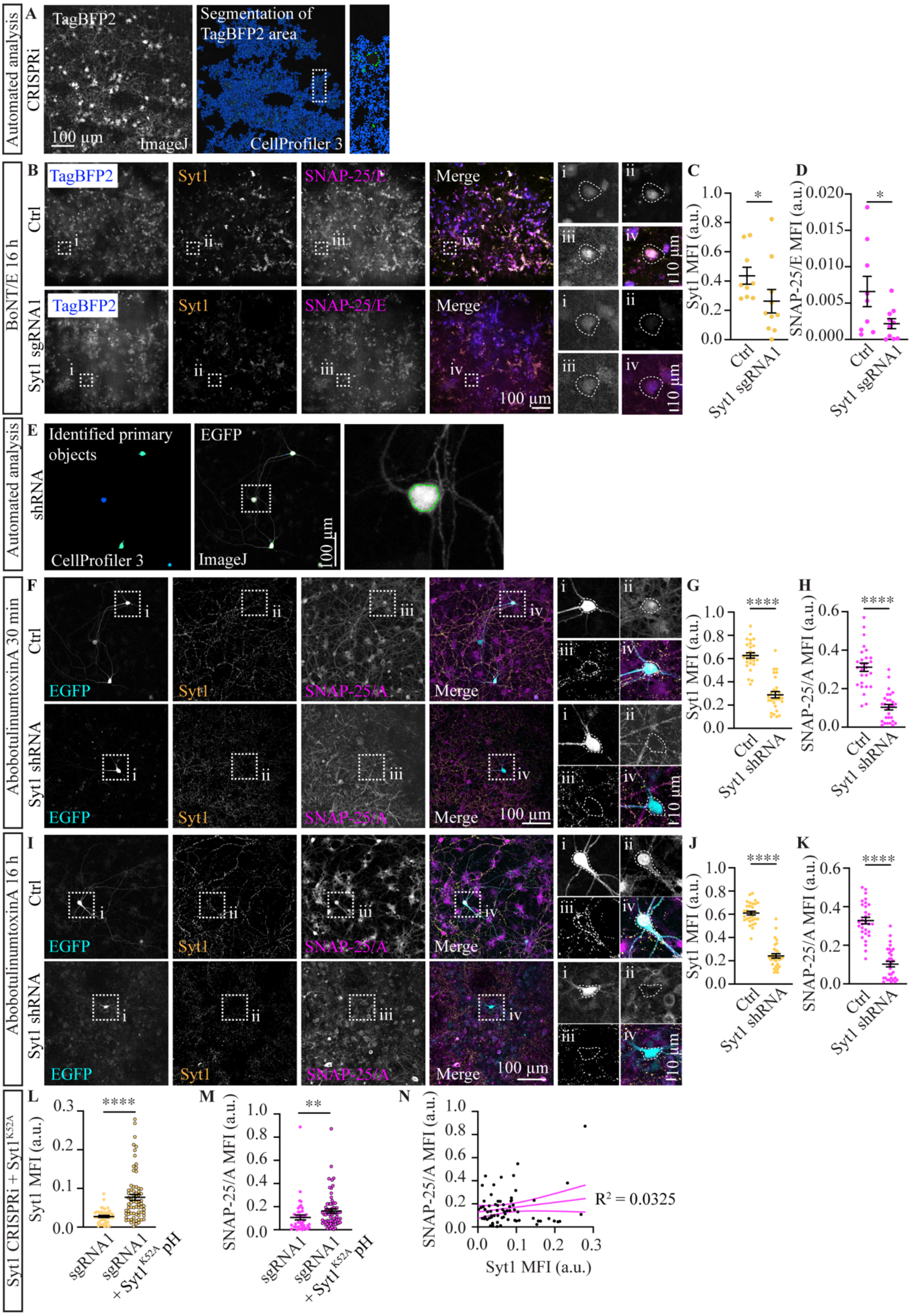

Automated analysis CRISPRi
A
TagBFP2
100 µm
ImageJ
Segmentation of TagBFP2 area
CellProfiler 3
BoNT/E 16 h
B
Ctrl
Syt1 sgRNA1
TagBFP2
Syt1
SNAP-25/E
Merge
i
ii
iii
iv
110 µm
100 µm
C
Syt1 MFI (a.u.)
1.0
0.8
0.6
0.4
0.2
0.0
*
Ctrl
Syt1 sgRNA1
D
SNAP-25/E MFI (a.u.)
0.020
0.015
0.010
0.005
0.000
*
Ctrl
Syt1 sgRNA1
Automated analysis shRNA
E
Identified primary objects
CellProfiler 3
EGFP
ImageJ
100 µm
Abobotulinumtoxin A 30 min
F
Ctrl
Syt1 shRNA
EGFP
Syt1
SNAP-25/A
Merge
100 µm
10 µm
G
Syt1 MFI (a.u.)
****
Ctrl
Syt1 shRNA
H
SNAP-25/A MFI (a.u.)
0.6
0.4
0.2
0.0
****
Ctrl
Syt1 shRNA
AbobotulinumtoxinA 16 h
I
Ctrl
Syt1 shRNA
EGFP
Syt1
SNAP-25/A
Merge
100 µm
10 µm
J
Syt1 MFI (a.u.)
****
Ctrl
Syt1 shRNA
K
SNAP-25/A MFI (a.u.)
****
Ctrl
Syt1 shRNA
Syt1 CRISPRi + Syt1K52A
L
Syt1 MFI (a.u.)
0.3
0.2
0.1
0.0
****
sgRNA1
sgRNA1 + Syt1K52A pH
M
SNAP-25/A MFI (a.u.)
**
sgRNA1
sgRNA1 + Syt1K52A pH
N
SNAP-25/A MFI (a.u.)
R² = 0.0325
Syt1 MFI (a.u.)
0.0
0.1
0.2
0.3

Appendix Figure S 7. Syt1 KD is protective over BoNT/A and BoNT/E neurointoxication

(A) Automated segmentation analysis for quantifying the mean fluorescence intensity (MFI) of Syt1 and AbobotulinumtoxinA-cleaved SNAP-25 (SNAP-25/A) levels following Syt1 KD with CRISPRi. Acquired confocal stacks were Z-projected using the sum of fluorescence in ImageJ (a representative image of TagBFP2 is shown in gray on left). The 2D Z-projections were then used in CellProfiler 3 to determine the area positive for TagBFP2, and to identify neurons that had received dCas9-KRAB and the respective sgRNA1-3. For segmentation of the TagBFP2-positive area (image in the middle), the brightest TagBFP2 fluorescent spots were identified using the Identify Primary Object tool of CellProfiler3 and the otzu algorithm (green dots; a detail from the indicated boxed area is magnified on right). Next, these primary spots were expanded following the TagBFP2 fluorescence signal until the whole TagBFP2 area of each image was detected (blue signal in the magnified image on right). The MFI of the Syt1 and cleaved SNAP-25 channels were then determined within this area.

(B) Representative confocal Z-stack sum projections of neurons transduced with TagBFP2-expressing (blue) control (ctrl; non-targeting sgRNA) and CRISPRi Syt1 sgRNA1 KD lentiviruses. Neurons were treated for 16 h with 10 nM BoNT/E, and immunostained for endogenous Syt1 (yellow) and BoNT/E-cleaved SNAP-25 (SNAP-25/E; magenta). Boxed regions (i-iv) are magnified on the right, with neuronal somas outlined by dashed lines for clarity.

(C, D) MFI quantification of endogenous (C) Syt1, and (D) cleaved SNAP-25 (SNAP-25/E), in control and Syt1 sgRNA1 KD neurons following 16 h BoNT/E treatment.

(E) Automated segmentation analysis tool used to quantify the MFI of Syt1 and SNAP-25/A levels following Syt1 KD using shRNA. The automated segmentation analysis was done as described above for the CRISPRi in (A), except for using the EGFP fluorescence signal to identify transfected cells. The values of threshold fluorescence intensity were chosen in CellProfiler 3 so that only the brightest signal of the EGFP-positive cells was automatically detected in all images. These bright areas corresponded to the neuronal soma (boxed area is shown magnified on the right), and the MFIs of Syt1 and cleaved SNAP-25/A were then quantified in these selected areas.

(F) Representative Z-projection sum of a confocal stack acquired from hippocampal neurons that were co-transfected with EGFP (cyan) and either pRNAi control (ctrl) or Syt1-targeting shRNA, and treated with 10 units of AbobotulinumtoxinA for 30 min, fixed and immunostained for endogenous Syt1 (yellow) and SNAP-25/A (magenta). Boxed regions (i-iv) are magnified on the right. Soma outlined with dashed line for clarity.

(G, H) Scatter plots of (G) endogenous Syt1 and (H) cleaved SNAP-25/A MFI in control (ctrl) and Syt1 shRNA-transfected hippocampal neurons following 30 min of AbobotulinumtoxinA treatment.

(I) Same as in (F) aside from the treatment period of AbobotulinumtoxinA being extended to 16 h.

(J, K) Scatter plots of (J) endogenous Syt1 and (K) cleaved SNAP-25/A MFI in control (ctrl) and Syt1 shRNA-transfected hippocampal neurons following 16 h of AbobotulinumtoxinA treatment.

(L, M) MFI quantification of endogenous Syt1 and SNAP-25/A in Syt1 sgRNA1 KD neurons with and without pLenti6.3-Syt1^{K52A}-pH rescue and treated with AbobotulinumtoxinA (10 units) for 30 min.

(N) The linear regression graph (± 95% confidence intervals) of Syt1 and SNAP-25/A MFIs is shown from a representative acquisition in Syt1 sgRNA1 KD neurons rescued with pLenti6.3-Syt1^{K52A}-pH. R^2 represents the proportion of the variance.

Data information: Results are shown as average ± standard error of the mean (±SEM). Scatter plots indicate averages from individual acquisitions. (G, H, J, K) n = 5-12 / condition from 2 independent experiments. Statistical significance was assessed using a parametric unpaired *t* test (G, D), and a non-parametric Mann-Whitney U test (C, H, J, K, L, M). *p<0.05, **p<0.01, ****p<0.0001.

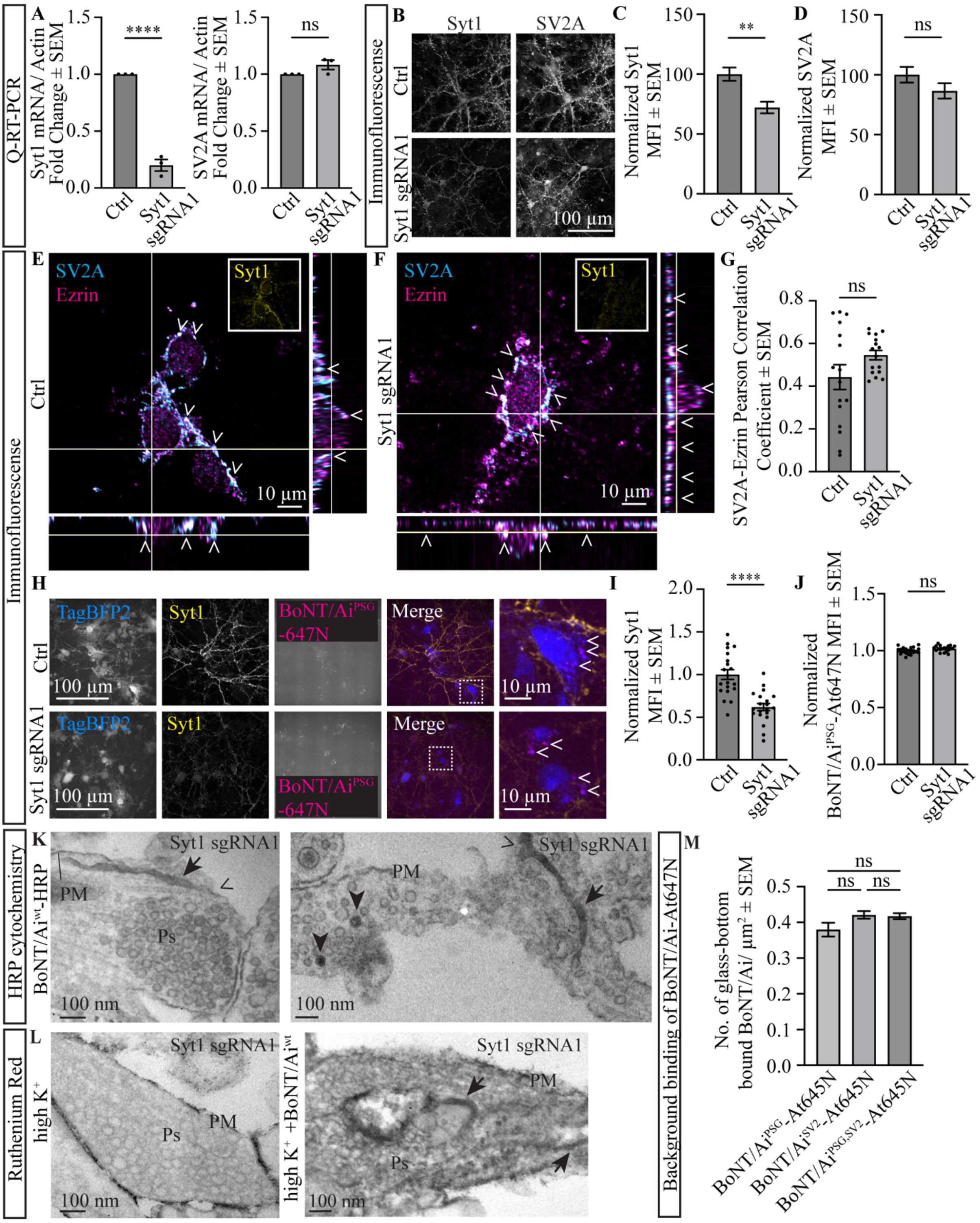

Q-RT-PCR
A
Syt1 mRNA/ Actin Fold Change ± SEM
SV2A mRNA/ Actin Fold Change ± SEM
****
ns
Ctrl
Syt1 sgRNA1
Immunofluorescense
B
Syt1
SV2A
Ctrl
Syt1 sgRNA1
100 μm
C
Normalized Syt1 MFI ± SEM
**
D
Normalized SV2A MFI ± SEM
ns
E
SV2A
Ezrin
Syt1
Ctrl
10 μm
F
Syt1 sgRNA1
G
SV2A-Ezrin Pearson Correlation Coefficient ± SEM
ns
H
TagBFP2
Syt1
BoNT/Ai^PSG -647N
Merge
100 μm
10 μm
I
Normalized Syt1 MFI ± SEM
****
J
Normalized BoNT/Ai^PSG-At647N MFI ± SEM
ns
HRP cytochemistry
K
BoNT/Ai^wt-HRP
Syt1 sgRNA1
PM
Ps
100 nm
Ruthenium Red
L
high K+
high K+ +BoNT/Ai^wt
Background binding of BoNT/Ai-At647N
M
No. of glass-bottom bound BoNT/Ai/ μm² ± SEM
ns
BoNT/Ai^PSG-At645N
BoNT/Ai^SV2-At645N
BoNT/Ai^PSG,SV2-At645N

Appendix Figure S 8. Syt1 KD does not induce changes in SV2A expression or neuronal localization.

(A) Q-RT-PCR analysis of Syt1 and SV2A mRNA fold change normalized to β-actin mRNA levels in non-targeting sgRNA1 (ctrl) neurons and following Syt1 sgRNA1 KD.

(B-D) Representative immunofluorescence staining of confocal Z-projection images (B) and mean fluorescence intensity (MFI) quantification of endogenous levels of (C) Syt1 and (D) SV2A following Syt1 sgRNA1 KD in hippocampal neurons.

(E-G) Representative confocal Z-projection images of hippocampal neurons immunostained against endogenous SV2A (cyan), Ezrin (magenta) and Syt1 (yellow) and represented with orthogonal projection in control neurons (E) and following Syt1 sgRNA1 KD (F), and the corresponding quantification of SV2A-Ezrin Pearson Correlation coefficient (G).

(H-J) Representative confocal Z-images of non-targeted sgRNA (ctrl) and Syt1 sgRNA1 KD hippocampal neurons with BoNT/Ai^{PSG}-At647N labelling (magenta, open arrowheads) and immunostaining against endogenous Syt1 (yellow) (H). TagBFP2 expression of the lentiviral construct in shown in blue. Quantification of MFI ± SEM of Syt1 (I) and BoNT/Ai^{PSG}-At647N (J) normalized to the average of the control in ctrl and Syt1 sgRNA1 KD hippocampal neurons.

(K) Representative electron microscopy images of tubular structures containing BoNT/Ai^{wt}-HRP signal (arrows) in the presynapses (Ps) of Syt1 sgRNA1-transduced neurons which remain open to the extracellular space (open arrowhead). BoNT/Ai^{wt}-HRP in synaptic vesicles (arrowheads) and plasma membrane (PM) are indicated for reference.

(L) Representative images of ruthenium red (RuR) stained Syt1 sgRNA1 KD hippocampal neuron presynapses (Ps) stimulated with high K^+ buffer alone, or in the presence of 100 pM unlabelled BoNT/Ai^{wt}. Tubular structures labelled with RuR (electron-dense precipitate) are indicated with arrows. Plasma membrane (PM) is indicated for reference.

(M) Quantification of the average number of BoNT/Ai^{PSG}-At647N, BoNT/Ai^{SV2}-At647N and BoNT/Ai^{PSG,SV2}-At647N molecules binding to poly-L-lysine-coated cell culture dish glass surface per area (µm^2).

Data information: Error bars are shown as standard error of the mean (±SEM). (A) N = 3, (C, D) n = 19-29 random regions of interest from 2 independent biological replicates, (G) n = 16 random regions of interest from 2 independent biological replicates (I, J) n = 20 random regions of interest from 2 independent biological replicates, and (M) n = 12-16 random regions of interest from 1 experiment/toxin. Unpaired t test in (A, C, D, I, J), non-parametric Mann-Whitney U test (G), and one-way ANOVA multiple comparison test (M). **p<0.01, ****p<0.0001, and ns – nonsignificant.
