# Supplementary material for: Presynaptic targeting of botulinum neurotoxin type A requires a tripartite PSG‐Syt1‐SV2 plasma membrane nanocluster for synaptic vesicle entry
Source: EMBO J. 2023 May 25;42(13):e112095. doi: 10.15252/embj.2022112095 (PMC10308369; doi:10.15252/embj.2022112095)
Supplement: Supplementary file 2 — Table EV1 [file EMBJ-42-e112095-s002.pdf]

REAGENT or RESOURCE	SOURCE	IDENTIFIER
Antibodies		
Anti-SV2A antibody	Abcam	Cat. #ab32942
Anti-SV2C antibody	Synaptic Systems	Cat. #119203
Anti-Synaptotagmin antibody	Abcam	Cat. #ab13259
Anti-Synapsin-1	Synaptic Systems	Cat. #06 011
Anti-SNAP-25/A	A kind gift from D. Sesardic	Division of Bacteriology, National Institute for Biological Standards and Control, NIBSC, Hertfordshire, United Kingdom
Anti-SNAP-25/E	A kind gift from D. Sesardic	Division of Bacteriology, National Institute for Biological Standards and Control, NIBSC, Hertfordshire, United Kingdom
Anti–β-actin clone AC-74	Sigma-Aldrich	Cat. #A5316
Anti-GFP sdAb antibody - FluoTag-Q At565	Synaptic Systems	Cat. #N0301-At565-S
Anti-mouse HRP-conjugated secondary antibody	Invitrogen	Cat #31430
Anti-rabbit HRP-conjugated secondary antibody	Invitrogen	Cat. #31460
Recombinant Anti-Ezrin antibody	Abcam	Cat. #ab40839
Reagents		
ATTO 647N NHS-ester	ATTO-TEC GmbH	Cat. #AD 647N-31
Ruthenium Red	Sigma-Aldrich	Cat. #00541
Bacterial and virus strains		
E. coli for production of active aBoNT/A^{wt} for MPN assay	Weisemann *et. al*., 2015	N/A
Chemicals, peptides, and recombinant proteins		
BoNT/Ai^{wt}	This paper	N/A
BoNT/Ai$^{W1266L/E224A/R363A/Y366F}$	This paper	N/A
BoNT/Ai$^{G1141D/G1292/E224A/R363A/Y366F}$	This paper	N/A
BoNT/Ai$^{W1266L/G1141D/G1292/E224A/R363A/Y366F}$	This paper	N/A
BoNT/A for MNP assay	This paper	N/A
Abobotulinumtoxin A	N/A	Ipsen; Dysport®
BoNT/E	A kind gift from T. Binz	Institute of Cellular Biochemistry, Hannover Medical School, Hannover, Germany

HEPES	Sigma-Aldrich	Cat. #H3375
Ascorbic acid	Sigma-Aldrich	Cat. #A5960
$CaCl_2$	Sigma-Aldrich	Cat. #C5080
BSA	Sigma-Aldrich	Cat. #A8022
$NaCl_2$	AMRESCO	Cat. #X190
D-glucose	AMRESCO	Cat. #0188
KCl	Ajax Finechem Pty Limited	Cat. #1206119
$MgCl_2$	Chem-Supply	Cat. #MA029
EDTA	ChemSupply	Cat #US004209
Nonidet P40 substitute	Sigma-Aldrich	Cat #11754599001
Complete EDTA-free protease inhibitor cocktail	Roche	Cat. #11873580001
Critical commercial assays		
Duolink™ In Situ Red Starter Kit Mouse/Rabbit	Merck	Cat. #DUO92101
Lightning-Link HRP Antibody Labeling Kit	Novus Biologicals	Cat. #NOV7010000
Lipofectamine 2000	Life Technologies	Cat. #11668019
KAPA HiFi HotStart ReadyMix	Roche	Cat. #7958927001
NEBuilder HiFi DNA Assembly Master Mix	NEB	Cat. #E2621
QuikChange Lightning Site-Directed Mutagenesis Kit	Agilent Technologies	Cat. #210518
RNeasy mini kit	Qiagen	Cat. #74004
High-capacity reverse transcription kit	Applied Biosystems	Cat. #4368814
Pierce™ BCA Protein Assay Kit	ThermoFiscer Scientific	Cat. #23225
PowerUpnTM SYBRTM Green Master Mix	ThermoFiscer Scientific	Cat. #A25741
Deposited data		
All raw data from image acquisitions	This paper	Will be deposited to UQ RDM upon acceptance of the manuscript

BoNT/A heavy chain complex with SV2A extracellular domain	Benoit *et al.*, 2014	PDB ID: 4JRA
Syt1 C2B domain complex with SV2 pT84 peptide	Zhang *et al.*, 2015	PDB ID: 4V11
BoNT/A heavy chain complex with GT1b	Stenmark *et al.*, 2008	PDB ID: 2VU9
BoNT/A full length (heavy chain and light chain)	Garcia-Rodriguez *et al.*, 2007	PDB ID: 2NYY
Modelled complex of Syt1 with GT1b	Flores *et al.*, 2019	N/A
AlphaFold2 model of SV2A	Jumper *et al.*, 2021; Tunyasuvunakool *et al.*, 2021	Uniprot ID: Q7L0J3. https://alphafold.ebi.ac.uk
AlphaFold2 model of Syt1	Jumper *et al.*, 2021; Tunyasuvunakool *et al.*, 2021	Uniprot ID: P21579. https://alphafold.ebi.ac.uk
Experimental models: Cell lines		
Lenti-XTM 293T Cell Line	TaKaRa	Cat. #632180
Experimental models: Organisms/strains		
Embryonic hippocampal neurons from Sprague Dawley Rats	Charles River Laboratories	https://www.criver.com
Swiss Mice	Janvier SA, France	N/A
Oligonucleotides		
Oligos for Synaptotagmin1 shRNA Sense: 5’-GATCCCCGAGCAAATCCAGAAAGTG CAATTCAAGAGATTGCACTTTCTGGA TTTGCTCTTTTTC 3’ and Antisense Oligo: 5’ -TCGAGAAAAAGA GCAAATCCAGAAAGTGCAATCTCTTG AATTG CACTTTCTGGATTTGCTCGGG 3’	This paper	N/A
Mutagenesis primers used for $Syt1^{K52A}$-pH Forward: 5’ – GTTTATGAATGAGCTGCATGCA	This paper	N/A

ATTCCATTGCCACCGTG 3’ and Reverse Primer: 5’- CACGGTGGCAATGGAATTGCATG CAGCTCATTCATAAAC 3’		
Mutagenesis oligos used to created K52A in Syt1-pH: Forward 5’ - GTTTATGAATGAGCTGCATGCAATTC CATTGCCACCGTG 3’ and Reverse Oligo 5’- CACGGTGGCAATGGAATTGCATGCAG CTCATTCATAAAC 3’.	This paper	N/A
Syt1 shRNA oligos: Sense Oligo 5’ - GATCCCCGAGCAAATCCAGAAAGTG CAATTCAAGAGA TTGCACTTTCTGGATTTGCTCTTTTTC 3’ and Antisense Oligo 5’ TCGAGAAAAA GAGCAAATCCAGAAAGTGCAA TCTCTTGAATTGCACTTTCTGGATTTG CTC GGG 3’.	This paper	N/A
Q-RT-PCR: rat SV2A forward 5′- CCAGATGGGCTCTGCTTACC-3′ and reverse 5′- CTCATCGTGCTTCCCATTCTCTA-3′	This paper	N/A
Q-RT-PCR: rat Syn1 forward 5′- AGCCATAGCCATAGTTGCGG-3′ and reverse 5′- CGTCATCCTTAAGGGCCTGAT-3′,	This paper	N/A
Q-RT-PCR: rat beta actin forward 5′- CCCGCGAGTACAACCTTCTTG-3′ and reverse 5′- GTCATCCATGGCGAACTGGTG-3′	This paper	N/A
Oligos for CRISPRi and rescue, see Table 1	This paper	N/A
Recombinant DNA		
Plasmid: SV2A^{wt}-pHluorin	Zhang *et al.*, 2015	N/A
Plasmid: SV2A^{T84A}-pHluorin	Zhang *et al.*, 2015	N/A
Plasmid: Syt1^{wt}-pHluorin (pCDNA3.1+ pHluorin Synaptotagmin1)	Diril *et al.*, 2006; Harper *et al.*, 2020	A kind gift from Prof. Volker Haucke (The Leibniz-Institute for Molecular Pharmacology)
Plasmid: Syt1^{K326A,K328A}-pHluorin	Zhang *et al.*, 2015	N/A
Plasmid: Syt1^{K52A}-pHluorin	This paper	Will be deposited to Addgene upon acceptance of the manuscript

Plasmid: Syt1 shRNA	This paper	N/A
Plasmid: pSUPER-neo+mCerulean	Clayton *et al.*, 2010	N/A
Lentiviral vector: pMDL g/p RRE	Addgene	Cat. #12251
Lentiviral vector: pRSV-RE	Addgene	Cat. #12253
Lentiviral vector: pMD2-G	Addgene	Cat. #12259
Lentiviral vector: pLenti6.3-Syt1^{wt}-pH	Addgene	Cat. #202551
Lentiviral vector: pLenti6.3-Syt1^{K52A}-pH	Addgene	Cat. #202552
Lentiviral vector: pLV-hUV6-sgRNA-dCas9-KRAB-TagBFP2 (identifier AAAA-0244)	Addgene	Cat. # 202553
Lentiviral vector: pLV-RnSyt1-sgRNA1-dCas9-KRAB-TagBFP2 (identifier AAAA-0245)	Addgene	Cat. # 202554
Lentiviral vector: pLV-RnSyt1-sgRNA2-dCas9-KRAB-TagBFP2 (identifier AAAA-0246)	Addgene	Cat. # 202555
Lentiviral vector: pLV-RnSyt1-sgRNA3-dCas9-KRAB-TagBFP2 (Identifier AAAA-0247)	Addgene	Cat. 202556
CRISPRi oligonucleotides, see Table 2	This paper	N/A
Software and algorithms		
Fiji (ImageJ)	Schneider *et al.*, 2012; Schindelin *et al.*, 2012	https://imagej.nih.gov/ij/
CellProfiler 3	BROAD Institute; McQuin *et al.*, 2018	https://cellprofiler.org/
MetaMorph (Microscopy Automation and Image Analysis Software)	Molecular Devices	https://www.moleculardevices.com/products/cellular-imaging-systems/acquisition-and-analysis-software/metamorph-microscopy
PALM-Tracer	Kechkar *et al.*, 2013 Nair *et al.*, 2013	N/A
TrackMate (plug-in for Fiji)	Tinevez et al., 2017	https://imagej.net/plugins/trackmate/
Huygens Professional 19.10 software	N/A	https://svi.nl/Huygens-Professional
iMOD	N/A	https://bio3d.colorado.edu/imod/
Python 2.7	N/A	https://python.org

MATLAB 2017b (for SharpVisu drift correction)	N/A	https://au.mathworks.com/matlabcentral/answers/498405-matlab-2017b-download
SharpViSu	Andronov *et al.,* 2016	https://github.com/andronovl/SharpViSu
NASTIC Nanocluster Analysis Tool	Wallis *et al.*, 2021 bioRXiv	N/A
Chop-Chop	Labun *et al.*, 2019<br>Labun *et al.*, 2016<br>Montague *et al.*, 2014	https://chopchop.cbu.uib.no
CRISPick	Kim *et al.*, 2018<br>DeWeirdt *et al.*, 2021	https://portals.broadinstitute.org/gppx/crispick/public
Benchling	Benchling	https://www.benchling.com
AlphaFold	Jumper *et al.,* 2021	https://github.com/deepmind/alphafold
Coot	Emsley *et al.*, 2004	https://www2.mrc-lmb.cam.ac.uk/personal/pemsley/coot/
Pymol	Schrodinger, USA	https://pymol.org/2/
GraphPad Prism 9 for macOS	N/A	https://www.graphpad.com/scientific-software/prism/
Adobe Illustrator 25.4.1 release	Adobe	N/A
Adobe Photoshop 22.4.3 release	Adobe	N/A
BioRender	BioRender	https://app.biorender.com
