# Supplementary material for: Presynaptic targeting of botulinum neurotoxin type A requires a tripartite PSG‐Syt1‐SV2 plasma membrane nanocluster for synaptic vesicle entry
Source: EMBO J. 2023 May 25;42(13):e112095. doi: 10.15252/embj.2022112095 (PMC10308369; doi:10.15252/embj.2022112095)
Supplement: Supplementary file 3 — Table EV2 [file EMBJ-42-e112095-s010.pdf]

Table EV2: CRISPRi oligonucleotides

Name	Sequence (5’->3’)	Description
Vec-pLV_R2	TCCTTAATCAGCTCGCTCATAGGGCCGGGATTCTCCTCCAC	Reverse primer for dCAS9-KRAB linearization
Vec-pLV_F2	ACTGGGGCACAAGCTTAATTGACCAGCACACTGGCGGC	Forward primer for dCAS9-KRAB linearization
TagBFP_F	CCTATGAGCGAGCTGATTAAGGAGAACATGC	Forward primer for TagBFP2 linearization
TagBFP_R	TCAATTAAGCTTGTGCCCCAGTTTGCTAG	Reverse primer for TagBFP2 linearization
RnSyt1-sgRNA1_F	CACCGCGTGCCTCGCACCGGTCCGCGG	Forward primer for gRNA1 against 5’-UTR of rat synaptotagmin-1 gene
RnSyt1-sgRNA1_R	AAACCCGCGGACCGGTGCGAGGCACGC	Reverse primer for gRNA1 against 5’-UTR of rat synaptotagmin-1 gene
RnSyt1-sgRNA2_F	CACCAGTACTCGCGTGCCTCGCACCGG	Forward primer for gRNA2 against 5’-UTR of rat synaptotagmin-1 gene
RnSyt1-sgRNA2_R	AAACCCGGTGCGAGGCACGCGAGTACT	Reverse primer for gRNA2 against 5’-UTR of rat synaptotagmin-1 gene
RnSyt1-sgRNA3_F	CACCTCCTCCTGCAGCGGCAGCATCGG	Forward primer for gRNA3 against 5’-UTR of rat synaptotagmin-1 gene
RnSyt1-sgRNA3_R	AAACCCGATGCTGCCGCTGCAGGAGGA	Reverse primer for gRNA3 against 5’-UTR of rat synaptotagmin-1 gene
Lenti_F	CGTACCGGTTAGTAATGATCGACAATCAACC	Forward primer used to amplify the pLenti6.3
Lenti_R	CGGAACTCCCAAGCTTATCGATAAAATTTTGA	Reverse primer used to amplify the pLenti6.3
prom-Syn1_F	TTATCGATAAGCTTGGGAGTTCCGCTGCAGAGGGCCCTGCGTATGAG	Forward primer used to amplify synaptotagmin-1 with synapsin-1 promoter
RnSyt1_R	TGTCGATCATTACTAACCGGTACGTTACTTCTTGACAGCCAGCATGGCATCAAC	Reverse primer used to amplify synaptotagmin-1 with synapsin-1 promoter
