# Supplementary material for: Presynaptic targeting of botulinum neurotoxin type A requires a tripartite PSG‐Syt1‐SV2 plasma membrane nanocluster for synaptic vesicle entry
Source: EMBO J. 2023 May 25;42(13):e112095. doi: 10.15252/embj.2022112095 (PMC10308369; doi:10.15252/embj.2022112095)
Supplement: Supplementary file 4 — Table EV3 [file EMBJ-42-e112095-s007.pdf]

Table EV3: CRISPRi and rescue plasmids

Unique identifier	Name	Description
AAAA-0238,	pLenti6.3-Syt1^{wt}-pH	*Rattus norvegicus* synaptotagmin1 under human synapsin1 promoter<br>Addgene Cat. #202551
AAAA-0239	pLenti6.3-Syt1^{K52A}-pH	*Rattus norvegicus* synaptotagmin1 with K52A mutation under human synapsin1 promoter<br>Addgene Cat. #202552
AAAA-0240	pLV-hUV6-sgRNA-dCas9-KRAB-Puro	Basic vector Addgene Cat. #71236
AAAA-0244	pLV-hUV6-sgRNA-dCas9-KRAB-TagBFP2	Nontargeting control vector with TagBFP2, this paper<br>Addgene Cat. #202553
AAAA-0245	pLV-RnSyt1-sgRNA1-dCas9-KRAB-TagBFP2	Synaptotagmin-1 sgRNA1 with TagBFP2, this paper<br>Addgene Cat. #202554
AAAA-0246	pLV-RnSyt1-sgRNA2-dCas9-KRAB-TagBFP2	Synaptotagmin-1 sgRNA2 with TagBFP2, this paper<br>Addgene Cat. #202555
AAAA-0247	pLV-RnSyt1-sgRNA3-dCas9-KRAB-TagBFP2	Synaptotagmin-1 sgRNA3 with TagBFP2, this paper<br>Addgene Cat. #202556
