# Supplementary material for: Presynaptic targeting of botulinum neurotoxin type A requires a tripartite PSG‐Syt1‐SV2 plasma membrane nanocluster for synaptic vesicle entry
Source: EMBO J. 2023 May 25;42(13):e112095. doi: 10.15252/embj.2022112095 (PMC10308369; doi:10.15252/embj.2022112095)
Supplement: Supplementary file 5 — Movie EV1 [file EMBJ-42-e112095-s008.zip › Movie EV1.rtf]

Movie EV1. Ribbon structure of BoNT/Ai. Mapped lysine-residues (magenta spheres), E224A/R363A/Y366F light chain (LC) mutations (orange spheres; toxin inactivation), and heavy chain (HC) receptor binding mutations W1266L (BoNT/APSG) and G1141D/G1292R (BoNT/ASV2) (cyan spheres).
