# Supplementary material for: Presynaptic targeting of botulinum neurotoxin type A requires a tripartite PSG‐Syt1‐SV2 plasma membrane nanocluster for synaptic vesicle entry
Source: EMBO J. 2023 May 25;42(13):e112095. doi: 10.15252/embj.2022112095 (PMC10308369; doi:10.15252/embj.2022112095)
Supplement: Supplementary file 6 — Movie EV2 [file EMBJ-42-e112095-s005.zip › Movie EV2.rtf]

Movie EV2. Dual-color single-molecule uPAINT imaging of BoNT/Aiwt-At647N and SV2Awt-pH/At565nb in live hippocampal neurons. Hippocampal neurons expressing SV2Awt-pH were stimulated with high K+ buffer supplemented with 100 pM BoNT/Aiwt-At647 and 100 pM anti-GFP At565nb and the mobility of SV2A-pH–bound At565nb (cyan) and the holotoxin (magenta) was recorded by total internal reflection fluorescence microscopy (50 Hz, 20 ms exposure time). Bar 10 µm and playback 50 frames s-1.
