# Supplementary material for: Presynaptic targeting of botulinum neurotoxin type A requires a tripartite PSG‐Syt1‐SV2 plasma membrane nanocluster for synaptic vesicle entry
Source: EMBO J. 2023 May 25;42(13):e112095. doi: 10.15252/embj.2022112095 (PMC10308369; doi:10.15252/embj.2022112095)
Supplement: Supplementary file 7 — Movie EV3 [file EMBJ-42-e112095-s001.zip › Movie EV3.rtf]

Movie EV3. Internalized BoNT/Aiwt-At647N imaged with sdTIM in live hippocampal neurons. Hippocampal neurons were stimulated for 5 min with high K+ buffer supplemented with 1 nM BoNT/Aiwt-At647 (magenta), washed, chased for 10 min in low K+ buffer, and imaged by TIRF microscopy (50 Hz, 20 ms exposure time). Playback 50 frames s-1. Hippocampal neurons were transfected with EGFP to outline transfected neurons from the underlying cultured neurons and a representative image of the EGFP is superimposed on the acquisition (white).
