# Supplementary material for: Presynaptic targeting of botulinum neurotoxin type A requires a tripartite PSG‐Syt1‐SV2 plasma membrane nanocluster for synaptic vesicle entry
Source: EMBO J. 2023 May 25;42(13):e112095. doi: 10.15252/embj.2022112095 (PMC10308369; doi:10.15252/embj.2022112095)
Supplement: Supplementary file 8 — Movie EV4 [file EMBJ-42-e112095-s009.zip › Movie EV4.rtf]

 Movie EV4. Electron tomographic analysis of the hippocampal neurons following CRISPRi Syt1 KD and induced uptake of BoNT/Aiwt-HRP. Syt1 KD was performed using CRISPRi sgRNA1 Syt1 KD in cultured hippocampal neurons (on DIV14 for 7 days), after which neurons were stimulated for 5 min with high K+ buffer supplemented with 5 µg mL-1 BoNT/Aiwt-HRP (dark precipitate), washed with low K+ buffer and chased for 10 min. Neurons were then fixed, cytochemically stained and processed for EM. Approximately 200 nm thick resin sections were subjected to electron tomography. The section shows an axonal segment on the left, and a presynaptic area on the right. The plasma membrane is modelled in green, synaptic vesicles in cyan, microtubules in yellow and tubular structures containing BoNT/Awt-HRP precipitate (highlighted in purple within yellow bounding boxes) in red. Bar 50 nm.
