# Supplementary material for: Presynaptic targeting of botulinum neurotoxin type A requires a tripartite PSG‐Syt1‐SV2 plasma membrane nanocluster for synaptic vesicle entry
Source: EMBO J. 2023 May 25;42(13):e112095. doi: 10.15252/embj.2022112095 (PMC10308369; doi:10.15252/embj.2022112095)
Supplement: Supplementary file 9 — Movie EV5 [file EMBJ-42-e112095-s003.zip › Movie EV5.rtf]

 Movie EV5. AlphaFold prediction of tripartite PSG-Syt1-SV2 nanocomplex and BoNT/A. Predictive model of the assembled PSG-Syt1-SV2A complex with bound BoNT/A on the plasma membrane. SV2AT84A, Syt1K326A,K328A and Syt1K52A, as well as the BoNT/A W1266L and G1141D/G1292R used in this study, are indicated.    
