# Supplementary material for: Presynaptic targeting of botulinum neurotoxin type A requires a tripartite PSG‐Syt1‐SV2 plasma membrane nanocluster for synaptic vesicle entry
Source: EMBO J. 2023 May 25;42(13):e112095. doi: 10.15252/embj.2022112095 (PMC10308369; doi:10.15252/embj.2022112095)
Supplement: Supplementary file 10 [file EMBJ-42-e112095-s004.pdf]

THE EMBO JOURNAL

Presynaptic targeting of botulinum neurotoxin type A requires a tripartite PSG-Syt1-SV2 plasma membrane nanocluster for synaptic vesicle entry

Frederic Meunier, Merja Joensuu, Parnayan Syed, Saber Saber, Vanessa Lanoue, Tristan Wallis, James Rae, Ailisa Blum, Rachel Gormal, Christopher Small, Shanley Sanders, Anmin Jiang, Stefan Mahrhold, Nadja Krez, Michael Cousin, Ruby Cooper-White, Justin Cooper-White, Brett Collins, Robert Parton, Giuseppe Balistreri, and Andreas Rummel
DOI: 10.15252/embj.2022112095

Corresponding author(s): Frederic Meunier (f.meunier@uq.edu.au) , Frederic Meunier (f.meunier@uq.edu.au), Merja Joensuu (m.joensuu@uq.edu.au)

Review Timeline:

Submission Date:	11th Jul 22
Editorial Decision:	14th Jul 22
Appeal Received:	7th Nov 22
Editorial Decision:	8th Dec 22
Revision Received:	19th Feb 23
Editorial Decision:	4th Apr 23
Revision Received:	18th Apr 23
Accepted:	28th Apr 23

Editor: Ieva Gailite

Transaction Report:

(Note: With the exception of the correction of typographical or spelling errors that could be a source of ambiguity, letters and reports are not edited. Depending on transfer agreements, referee reports obtained elsewhere may or may not be included in this compilation. Referee reports are anonymous unless the Referee chooses to sign their reports.)

Dear Merja,

Thank you for submitting your manuscript "BoNT/A targets Syt1-PSG-SV2 surface nanoclusters for synaptic vesicle entry and neurointoxication" for consideration by The EMBO Journal. I have now read your study carefully and discussed it with my colleagues and an external scientific advisor. I am sorry to say that we have decided not to pursue publication of the manuscript at The EMBO Journal. However, our sister journal Life Science Alliance would be happy to offer peer review if you were to transfer your manuscript there.

We appreciate that your study presents a state-of-art imaging analysis of inactivated botulinum holotoxin BoNT/A uptake into hippocampal neurons. The findings show that in addition to its known cell surface receptors polysialogangliosides (PSGs) and synaptic vesicle glycoprotein 2A (SV2A), BoNT/A requires synaptotagmin 1 (Syt1) for its entry into synaptic vesicles. Furthermore, BoNT/A induces formation of plasma membrane nanoclusters containing Syt1, SV2A and PSG, suggesting a tripartite protein complex involvement in BoNT/A uptake and neurotoxicity.

While we appreciate the high quality of the study and realise that the presented findings will be of interest to the more immediate research field, we also noted that both SV2 and Syt1/2 are known receptors of particular botulinum toxin subtypes, and the interaction between SV2A and Syt1 is known to mediate Syt1 retrieval into synaptic vesicles. Furthermore, our external scientific advisor highlighted potential alternative explanations for the observed relevance of Syt1 for BoNT/A uptake. Therefore, I am afraid we concluded your current manuscript is not a sufficiently strong candidate for peer review and publication in The EMBO Journal.

That being said, your work would be an excellent candidate for our partner journal Life Science Alliance (http://www.life-science-alliance.org/; our broad scope Open Access journal published in partnership between the EMBO, Rockefeller University, and Cold Spring Harbor Laboratory Presses). The editors of Life Science Alliance would be pleased to send your manuscript for in-depth peer review; no reformatting is required. We very much hope you will be interested in this option: please follow the link below for transfer. Eric Sawey, Executive Editor of Life Science Alliance (e.sawey@life-science-alliance.org) will be happy to answer any questions you may have.

Link Not Available

Thank you for giving us the opportunity to consider this manuscript. I am sorry that I could not offer better news this time, and I hope that you will find the transfer option of interest. I will also be happy to discuss further during our video call tomorrow morning.

With kind regards,

Ieva

Ieva Gailite, PhD
Scientific Editor
The EMBO Journal
Meyerhofstrasse 1
D-69117 Heidelberg
Tel: +4962218891309
i.gailite@embojournal.org

External advisor's comments:
Unfortunately I was not fully satisfied by the experimental approach which has been deployed to address this hypothesis / model. Whereas the model is in principle sound (and already largely accepted by the community), a consequence of SytI downregulation is a drop in SV2A availability on the synaptic membrane as a result of a change of synaptic vesicle membrane dynamics, which reduces as a consequence the availability of the primary receptor of BoNT/A. Some experiments to control for this likely eventuality have been done, but in my view they provide correlative evidence more than a conclusive proof that this is not the case.

** As a service to authors, EMBO Press provides authors with the possibility to transfer a manuscript that one journal cannot offer to publish to another EMBO publication or the open access journal Life Science Alliance launched in partnership between EMBO Press, Rockefeller University Press and Cold Spring Harbor Laboratory Press. The full manuscript and if applicable, reviewers' reports, are automatically sent to the receiving journal to allow for fast handling and a prompt decision on your manuscript. For more details of this service, and to transfer your manuscript please click on Link Not Available. **

November 7th, 2022

Re: EMBO J manuscript # EMBOJ-2022-112095

Dear Dr Gailite,

On behalf of our collaborators, please find enclosed a revised version of the manuscript entitled: "Nanoscale intoxication roadmap of BoNT/A: synaptotagmin 1 is critical for synaptic vesicle targeting" for your consideration as a research article in The EMBO Journal.

Based on the Editorial and External Assessor feedback, we have conducted extensive additional experiments and analyses to enhance the functional significance of our study, and to expand the proposed unifying tripartite model of intoxication to another BoNT serotype. We have also reformatted the manuscript, and removed supportive results not directly connected to the main finding, to simplify this rather extensive study. Following the feedback of two Editorial Zoom meetings, we have conducted additional experiments using BoNT serotype E (BoNT/E), which also targets gangliosides and SV2 as its main receptors. We demonstrate that, like BoNT/A, BoNT/E also requires synaptotagmin 1 for its neurointoxication. These results support our proposed model of a unifying tripartite synaptotagmin 1-ganglioside-SV2 neurointoxication mechanism of clostridial neurotoxins. Reflecting these changes, we changed the manuscript title to be more synaptotagmin 1-centric.

We would like to thank the external assessor for their insights, as we have been able to significantly improve manuscript since the initial submission. Although the assessor agreed that our proposed model is in principle sound, they raised an important concern of the consequences of synaptotagmin 1 downregulation to SV2A availability on the synaptic membrane. To address this, we have investigated the effects of synaptotagmin 1 knockdown on SV2A mRNA and protein expression levels, as well as localization of SV2A on the neuronal plasma membrane, and demonstrate that the synaptotagmin 1 knockdown does not induce significant changes in SV2A expression or plasma membrane localization in cultured hippocampal neurons. We also demonstrate that the plasma membrane binding of BoNT/Ai^{PSG} (a receptor binding site mutant of the toxin that is only able to bind to SV2), was not affected by synaptotagmin 1 knockdown, indicating unperturbed access of the toxin to its primary receptor SV2.

The Editorial feedback highlighted SV2 and Syt1/2 as known receptors of botulinum neurotoxin serotypes, and the interaction between of SV2A and Syt1 to mediate Syt1 retrieval into synaptic vesicles. The external assessor also pointed out that our model was already largely accepted by the community. While we agree that the clostridial neurotoxin receptors have mainly been described, and that SV2-synaptotagmin 1 interactions have been shown to control recycling of synaptotagmin 1 into synaptic vesicles, we disagree with this statement in regard to the novelty of our finding. While the different clostridial neurotoxins have been shown to target mutually exclusively either SV2 or synaptotagmin1/2, our study identifies a tripartite requirement for synaptotagmin 1, SV2 and gangliosides for synaptic vesicle targeting of BoNT/A and BoNT/E is novel and has not been proposed or described before. Our results reveal for the first time that synaptotagmin 1 has a central role in the targeted endocytosis of BoNT/A into synaptic vesicles, while the toxin exclusively binds to gangliosides and SV2. We demonstrate for the first time that BoNT/A binding to plasma membrane gangliosides serves as a molecular bridge that engages the ganglioside-bound synaptotagmin 1 to nanocluster with SV2, which is a prerequisite for targeted endocytosis into synaptic vesicles. This goes far beyond what was known before in the field of endocytosis and neurotoxins and creates a frame shift in our understanding of the clostridial neurotoxin intoxication mechanism and synaptic vesicle targeting.

We hope that you will deem this version of our manuscript suitable for publication in The EMBO Journal. We look forward to hearing from you soon.

Yours sincerely, Merja Joensuu, PhD and Frederic A. Meunier, PhD

Dear Merja,

Thank you for submitting your manuscript for consideration by the EMBO Journal. We have now received comments from two reviewers, which are included below for your information.

As you will see from the reports, the reviewers appreciate the work, while also indicating a few aspects that would need to be strengthened before acceptance here. From my side, I find the reviewer comments generally reasonable. Therefore, based on these positive assessments, I would like to invite you to address the issues raised by the reviewers in a revised manuscript, especially focusing on addressing the issues regarding the specificity of the Syt1 role in botulinum toxin uptake as highlighted by reviewer #2. I would be happy to discuss the revision in more detail via email or phone/videoconferencing.

We generally allow three months as standard revision time. As a matter of policy, competing manuscripts published during this period will not negatively impact on our assessment of the conceptual advance presented by your study. However, please contact me as soon as possible upon publication of any related work to discuss the appropriate course of action. Should you foresee a problem in meeting this three-month deadline, please contact us to arrange an extension.

When preparing your letter of response to the referees' comments, please bear in mind that this will form part of the Review Process File and will therefore be available online to the community. For more details on our Transparent Editorial Process, please visit our website: https://www.embopress.org/page/journal/14602075/authorguide#transparentprocess. Please also see the attached instructions for further guidelines on preparation of the revised manuscript.

Please feel free to contact me if you have any further questions regarding the revision. Thank you for the opportunity to consider your work for publication, and I look forward to your revision.

With best regards,

Ieva

Ieva Gailite, PhD
Senior Scientific Editor
The EMBO Journal
Meyerhofstrasse 1
D-69117 Heidelberg
Tel: +4962218891309
i.gailite@embojournal.org

Instructions for preparing your revised manuscript:

Please make sure you upload a letter of response to the referees' comments together with the revised manuscript.

Please also check that the title and abstract of the manuscript are brief, yet explicit, even to non-specialists.

When assembling figures, please refer to our figure preparation guideline in order to ensure proper formatting and readability in print as well as on screen:
https://bit.ly/EMBOPressFigurePreparationGuideline
See also guidelines for figure legends: https://www.embopress.org/page/journal/14602075/authorguide#figureformat

At EMBO Press we ask authors to provide source data for the main manuscript figures. Our source data coordinator will contact you to discuss which figure panels we would need source data for and will also provide you with helpful tips on how to upload and organize the files.

IMPORTANT: When you send the revision we will require

- a point-by-point response to the referees' comments, with a detailed description of the changes made (as a word file).
- a word file of the manuscript text.
- individual production quality figure files (one file per figure)
- a complete author checklist, which you can download from our author guidelines (https://www.embopress.org/page/journal/14602075/authorguide).
- Expanded View files (replacing Supplementary Information)

Please see out instructions to authors
https://www.embopress.org/page/journal/14602075/authorguide#expandedview

Please remember: Digital image enhancement is acceptable practice, as long as it accurately represents the original data and conforms to community standards. If a figure has been subjected to significant electronic manipulation, this must be noted in the figure legend or in the 'Materials and Methods' section. The editors reserve the right to request original versions of figures and the original images that were used to assemble the figure.

Further information is available in our Guide For Authors: https://www.embopress.org/page/journal/14602075/authorguide

We realize that it is difficult to revise to a specific deadline. In the interest of protecting the conceptual advance provided by the work, we recommend a revision within 3 months (8th Mar 2023). Please discuss the revision progress ahead of this time with the editor if you require more time to complete the revisions.

Referee #1:

In this article, the authors used super-resolution microscopy to characterize the binding and entry of Botulinum neurotoxin A in cultured neurons. They were able to decipher the role of a preassembled polygangliosides-Synaptotagmin 1 complex, and Synaptotagmin 1/SV2 nanoclusters which are involved in toxin endocytosis. They further extend their finding to BoNTE and propose a general mechanism for BoNT entry.

This is a very convincing and exhaustive article based on tout-de-force experiments using in particular Atto-holotoxin at low concentration (100pM) which corresponds to physiological conditions and uPAIN (Universal Point Accumulation Imaging in Nanoscale Topography), a single-molecule super-resolution method. The experiments were well-designed and controlled. The results are fairly discussed. This article will have a strong impact on the field of neurotoxins and the cell biology of the pre-synaptic compartment. Indeed, the mechanism of BoNT intoxication has focused attention for a long time without definitive answers mainly for technical reasons which are well addressed here thanks to super-resolution imaging.

The only potential addition which would further add to the proposed mechanism relates to the role of PIP2 which binds Synaptotagmin 1 and was shown to be involved in the entry of BoNTs. Could the authors test whether or not the Synaptotagmin 1/SV2 nanoclusters are also enriched in PIP2? Would dephosphorylation of PIP2 such as by Synaptojanin 1 alter the Synaptotagmin 1/SV2 nanoclusters and prevent toxin entry?

Referee #2:

Background
Botulinum neurotoxins include seven major serotypes (BoNT/A-G). BoNT/A is the most important member, as it is the dominant type used as a medicine (Botox). BoNTs are known to recognize polysialogangliosides (PSG) with low affinity, which are enriched in neuronal membranes. A ganglioside-binding site is well defined, and mutations that disrupt BoNT/A binding to gangliosides have been reported and validated. It is also well established that two synaptic vesicle membrane proteins, synaptotagmin 1 and 2 (Syt1/2) and SV2, are utilized by distinct BoNTs as specific protein receptors, including SV2 for BoNT/A and Syt1/2 for BoNT/B. The structure of BoNT/A-SV2 has been solved and mutations that disrupt BoNT/A binding to SV2 have been reported and validated.
By utilizing synaptic vesicle proteins as receptors, BoNTs enter neurons through synaptic vesicle exocytosis-endocytosis pathway. Syt1/2 are essential Ca2+ sensors for regulating synaptic vesicle exocytosis, and lack of Syt1/2 blocks synchronized release of vesicles. Syt1 is also well known to play a key role in endocytosis of synaptic vesicle proteins, which appear to cluster and recycle together as a synaptic iTRAPs (Cousin, 2017, cited here). The function of SV2 remains unknown, but it has been well established that SV2 interacts with Syt1 and forms a complex. Lacking SV2 reduces Syt1 and disrupts Syt1 endocytosis. Specific mutations in SV2 that disrupt SV2-Syt1 interactions have been reported.
A previous model proposed that PSG acts to enrich toxins and increase their chances of binding to protein receptors. Recent studies suggested that Syt1/2 can bind to PSG directly and the Syt-PSG complex presents the high affinity binding site to BoNT/B (Flores et al., 2019; Ramirez-Franco et al., 2022, both cited here). A specific mutation in Syt1 (K52A) that disrupt Syt-PSG complex has been reported.
New findings and strength
There is no evidence for SV2 to form a complex with PSG. Whether there are any synergistic mechanisms between SV2-binding

and PSG-binding for BoNT/A, beyond two binding sites, remains unknown and difficult to study.
Here Joensuu et al utilized a series of high-tech single molecule super-resolution imaging approaches (uPAINT, sdTIM, HILO, dual color, NASTIC etc) plus EM, took advantage of previously established specific mutations (in BoNT/A, SV2, or Syt1), and analyzed binding and entry of BoNT/A on cultured hippocampal neurons in figures 1-3, which is the strong part of the manuscript. They reported that (1) binding to both PSG and SV2 is important for efficient entry of BoNT/A into neurons, (2) binding of BoNT/A reduces SV2 mobility on neuronal surfaces; (3) this reduction of SV2 mobility depends on both PSG binding and SV2-Syt1 interactions; (4) binding of BoNT/A enhances SV2-Syt1 interactions and elevates clustering of SV2-Syt1.
Based on these findings, together with previous findings on PSG-Syt1 complex, SV2-Syt1 interactions, and the role of Syt1 in endocytosis, the authors proposed an interesting model that BoNT/A recognizes PSG complexed with Syt1. Thus, PSG-binding brings Syt1 to be close to SV2 and increases formation of SV2-Syt1 clustering. As Syt1 is important for endocytosis, formation of SV2-Syt1 complex promotes endocytosis of the toxin. Here the role of PSG is not limited to be a low-affinity binding site but also acts to recruit the endocytosis factor Syt1, leading to efficient internalization of BoNT/A into the correct endosomal compartment in neurons. By actively promoting SV2-Syt1 complex, BoNTs amply the existing synaptic vesicle protein endocytosis mechanism to facilitate their own entry into neurons. The model is appealing and further deepens our understanding on the role of PSG-binding for BoNTs that bind to SV2 (BoNT/A and BoNT/E) and endocytosis of toxins.
Major weakness and issues
1. The writing of the manuscript is disappointing for this otherwise exciting study. Although I understand the motivation to claim "new" "novel", "first", and to claim the toxin binding "inducing" the forming of Syt1-SV2 complex etc, the authors have taken these unscientific and inaccurate claims to extreme degrees (title, abstract, introduction and the first part of discussion). This is very annoying as the authors apparently are aware of previous studies (in the Result section). Making these claims and disregarding previous literatures are harmful and unnecessary - the writing needs to be re-focused on describing their findings accurately in the context of previous publications, in scientifically plain language.
Title: the current title is more proper for a review article - it needs to be tuned it down and reflect the actual content of this paper.
Abstract: rewrite to describe the actual content of the paper (specific experiments and findings), delete all "novel" "first' claims and other unnecessary sentences not relevant to the experiments.
Introduction: the authors need to thoroughly discuss the previous work relevant to this study in the introduction and cite these papers here (see the background section above, on PSG-Syt1, SV2-Syt1, Syt1-endocytosis etc. add Chen et al, PNAS, 2022, 119 (20) e2111051119; Yao et al., JNeuroscience, 2012, 32:3778. Colasante et al, 2013, Mol. Neurobiol., 48:120.)
Discussion: The first section is repetitive and can be deleted. The rest needs to be tuned down on claims and add the discussion on technical limitations and potential alternative explanations.
"inducing": SV2-Syt1 interact and form complexes without BoNT/A. The word "inducing" is misleading. It can be replaced with "enhancing", "increasing", "elevating", "facilitating", etc. The surprise is not that SV2-Syt1 form a complex, but that the complex formation is not saturated and can still be further promoted with BoNT/A.
2. In contrast to Figures 1-3, Figures 4-5 are less relevant, overinterpreted, and some data are problematic. Syt1 is essential for SV exocytosis and endocytosis recycling, therefore it is not surprising that KD of Syt1 reduces BoNT/A and BoNT/E entry, as the entry pathway is disrupted. This part is over-exaggerated unnecessarily, without acknowledging many of the previous studies (e.g. Chen et al, PNAS, 2022, 119 (20) e2111051119; Yao et al., JNeuroscience, 2012, 32:3778; Willig et al, Nature, 2006, 440:935; Liu et al, J. Neurosci. 2009, 29:7395). There are insufficient controls to differentiate differences in the general role of Syt1 in synaptic vesicle recycling from any specific role in BoNT/A's selective uptake.
Figure 4: examining cleavage of SNAP-25 is not meaningful here as SV exo-endo recycling is disrupted. It would be more meaningful to do a more careful study of BoNT/A endocytosis, using authors' high-tech approach, in the presence of Syt1 (K52A) in Syt1 KO neurons.
Figure 5: the mIPSCs data are less relevant and problematic. Syt1 KO and KD neurons should show higher mIPSC frequency, not lower, than the control (Coutney et al, Nat. Commun. 2019, 10:4076). This part is unnecessary and can be taken out. The tubules in Figure 5 are interesting findings, but missing the controls on specificity. A recent study indicates the development of large tubular structures occurs in Syt1 KO neurons using HRP as a traditional lipophilic label (Chen et. al 2022, PNAS, PMID: 35537054), indicating these effects are unrelated to botulinum neurotoxin, but ascribed to loss of synaptic endocytosis and increase of large-sized endocytosis.
Syt1 KD in cultured hippocampal neurons by DIV21-22 days will reduce the amount of SV2, and the total vesicle numbers (e.g. Liu et al, J. Neurosci. 2009, 29:7395). The authors' western blot data on this point is problematic (Fig. S8). As the authors showed reduction of Syt1 using imaging approaches in Figure 4, the level of SV2 needs to be validated using the same approach.
One solution is to expand figures 1-3 into more figures and minimize figures 4-5, to re-focus on the strong part of this work, rather than the Syt1 KD part.
3. Figure 2D: the impact of BoNT/A on Syt1 mobility is rather small (0.29 to 0.26?). The authors need to re-check it by averaging technical replicates within a biological replicate and replot and compare the mean of each biological replicate. Additional repeats (ideally done in blind) might be needed to confirm the results.
Other minor points
Introduction:
2nd paragraph line 1: missing a blank before E.
2nd paragraph line 4: of glycoproteins.
3rd paragraph line 1-3: many previous studies utilized full length active toxins.
Results:
Line 1-3: Authors need to tune down their claims and delete line 1-3.

Page 3, last line and Page 4, 3rd paragraph, line 4-9: as the double KO showed no further reduction in binding compared with PSG KO, there is lack of evidence for SV2-mediated binding here. The author cannot assume that these binding observed for PSK mutant is mediated by SV2.
Page 4, line 16: what is "cytochemically"?
BoNT/A is in SV: needs to cite previous paper: Colasante et al, 2013, Mol. Neurobiol., 48:120.
Page 7 line: description of BoNT/E was repeated.
What is this TagBFP2?
"which fits with earlier findings that Syt1 KD perturbs the Ca2+-sensor function of Syt1 in evoked synchronous release": This sentence does not make much sense here - there is no study of evoked release here.
Needs to cite references for Stonin and AP2.
Syt1 is not a "must", but rather facilitates the endocytosis efficacy.

Referee #1:

In this article, the authors used super-resolution microscopy to characterize the binding and entry of Botulinum neurotoxin A in cultured neurons. They were able to decipher the role of a preassembled polygangliosides-Synaptotagmin 1 complex, and Synaptotagmin 1/SV2 nanoclusters which are involved in toxin endocytosis. They further extend their finding to BoNTE and propose a general mechanism for BoNT entry.

This is a very convincing and exhaustive article based on tout-de-force experiments using in particular Atto-holotoxin at low concentration (100pM) which corresponds to physiological conditions and uPAIN (Universal Point Accumulation Imaging in Nanoscale Topography), a single-molecule super-resolution method. The experiments were well-designed and controlled. The results are fairly discussed. This article will have a strong impact on the field of neurotoxins and the cell biology of the pre-synaptic compartment. Indeed, the mechanism of BoNT intoxication has focused attention for a long time without definitive answers mainly for technical reasons which are well addressed here thanks to super-resolution imaging.

The only potential addition which would further add to the proposed mechanism relates to the role of PIP2 which binds Synaptotagmin 1 and was shown to be involved in the entry of BoNTs. Could the authors test whether or not the Synaptotagmin 1/SV2 nanoclusters are also enriched in PIP2? Would dephosphorylation of PIP2 such as by Synaptojanin 1 alter the Synaptotagmin 1/SV2 nanoclusters and prevent toxin entry?
These are interesting questions, but we feel that addressing these would deserve a full and detailed investigation in a separate follow up study. However, we have discussed these points in the discussion section as follows: “BoNT/A-Hc has been shown to internalize into uncoated SVs and clathrin-coated vesicles [21-24,72]. Clathrin-coated pits are formed by the association of an array of endocytic proteins including clathrin heavy and light chains, as well as AP-2, and are regulated by membrane lipid phosphatidylinositol 4,5-bisphosphate (PIP2) [73]. Syt1 has been shown to bind to membrane domains enriched in PIP2 [74] which can be cleaved by synaptojanin-1 to facilitate membrane fission [75]. Whether Syt1 and SV2 form nanoclusters on the plasma membrane patches that are enriched in PIP2, remain to be determined. Our results suggest that the endocytic sorting of the toxin depends on tripartite interactions within the PSG-Syt1-SV2 nanocluster. It is likely that downstream of these events, other endocytic machinery proteins, such as stonin-2 [28-32], are also involved in the endocytic process of BoNT/A.”

Referee #2:

Background
Botulinum neurotoxins include seven major serotypes (BoNT/A-G). BoNT/A is the most important member, as it is the dominant type used as a medicine (Botox). BoNTs are known to recognize polysialogangliosides (PSG) with low affinity, which are enriched in neuronal membranes. A ganglioside-binding site is well defined, and mutations that disrupt BoNT/A binding to gangliosides have been reported and validated. It is also well established that two synaptic vesicle membrane proteins, synaptotagmin 1 and 2 (Syt1/2) and SV2, are utilized by distinct BoNTs as specific protein receptors, including SV2 for BoNT/A and Syt1/2 for BoNT/B. The structure of BoNT/A-SV2 has been solved and mutations that disrupt BoNT/A binding to SV2 have been reported and validated.
By utilizing synaptic vesicle proteins as receptors, BoNTs enter neurons through synaptic vesicle exocytosis-endocytosis pathway. Syt1/2 are essential Ca2+ sensors for regulating synaptic vesicle exocytosis, and lack of Syt1/2 blocks synchronized release of vesicles. Syt1

is also well known to play a key role in endocytosis of synaptic vesicle proteins, which appear to cluster and recycle together as a synaptic iTRAPs (Cousin, 2017, cited here). The function of SV2 remains unknown, but it has been well established that SV2 interacts with Syt1 and forms a complex. Lacking SV2 reduces Syt1 and disrupts Syt1 endocytosis. Specific mutations in SV2 that disrupt SV2-Syt1 interactions have been reported.
A previous model proposed that PSG acts to enrich toxins and increase their chances of binding to protein receptors. Recent studies suggested that Syt1/2 can bind to PSG directly and the Syt-PSG complex presents the high affinity binding site to BoNT/B (Flores et al., 2019; Ramirez-Franco et al., 2022, both cited here). A specific mutation in Syt1 (K52A) that disrupt Syt-PSG complex has been reported.

New findings and strength
There is no evidence for SV2 to form a complex with PSG. Whether there are any synergistic mechanisms between SV2-binding and PSG-binding for BoNT/A, beyond two binding sites, remains unknown and difficult to study.
Here Joensuu et al utilized a series of high-tech single molecule super-resolution imaging approaches (uPAINT, sdTIM, HILO, dual color, NASTIC etc) plus EM, took advantage of previously established specific mutations (in BoNT/A, SV2, or Syt1), and analyzed binding and entry of BoNT/A on cultured hippocampal neurons in figures 1-3, which is the strong part of the manuscript. They reported that (1) binding to both PSG and SV2 is important for efficient entry of BoNT/A into neurons, (2) binding of BoNT/A reduces SV2 mobility on neuronal surfaces; (3) this reduction of SV2 mobility depends on both PSG binding and SV2-Syt1 interactions; (4) binding of BoNT/A enhances SV2-Syt1 interactions and elevates clustering of SV2-Syt1.

Based on these findings, together with previous findings on PSG-Syt1 complex, SV2-Syt1 interactions, and the role of Syt1 in endocytosis, the authors proposed an interesting model that BoNT/A recognizes PSG complexed with Syt1. Thus, PSG-binding brings Syt1 to be close to SV2 and increases formation of SV2-Syt1 clustering. As Syt1 is important for endocytosis, formation of SV2-Syt1 complex promotes endocytosis of the toxin. Here the role of PSG is not limited to be a low-affinity binding site but also acts to recruit the endocytosis factor Syt1, leading to efficient internalization of BoNT/A into the correct endosomal compartment in neurons. By actively promoting SV2-Syt1 complex, BoNTs amply the existing synaptic vesicle protein endocytosis mechanism to facilitate their own entry into neurons. The model is appealing and further deepens our understanding on the role of PSG-binding for BoNTs that bind to SV2 (BoNT/A and BoNT/E) and endocytosis of toxins.
Major weakness and issues
1. The writing of the manuscript is disappointing for this otherwise exciting study. Although I understand the motivation to claim "new" "novel", "first", and to claim the toxin binding "inducing" the forming of Syt1-SV2 complex etc, the authors have taken these unscientific and inaccurate claims to extreme degrees (title, abstract, introduction and the first part of discussion). This is very annoying as the authors apparently are aware of previous studies (in the Result section). Making these claims and disregarding previous literatures are harmful and unnecessary - the writing needs to be re-focused on describing their findings accurately in the context of previous publications, in scientifically plain language.
We are very sorry that we gave this impression. Our claim is that the tripartite nanocluster acts as a portal during BoNT/A intoxication, and we demonstrate a central role of Syt1 in this process. To the best of our knowledge, this has not been proposed or described prior to our study. Hence, we felt that the use of “novel” was warranted in this instance. However, the

referee is right and we have now removed the use of "new" "novel", and "first" throughout the manuscript including the abstract and can assure the reviewer that any potential disregard of previous publications and results has not been intentional.

Title: the current title is more proper for a review article - it needs to be tuned it down and reflect the actual content of this paper.
We have now modified the title to "A tripartite PSG-Syt1-SV2 plasma membrane nanocluster sorts BoNT/A into synaptic vesicles".

Abstract: rewrite to describe the actual content of the paper (specific experiments and findings), delete all "novel" "first' claims and other unnecessary sentences not relevant to the experiments.
We have reformatted the abstract, focusing on the description of experimental findings, and have removed unnecessary mentions of novelty.

Introduction: the authors need to thoroughly discuss the previous work relevant to this study in the introduction and cite these papers here (see the background section above, on PSG-Syt1, SV2-Syt1, Syt1-endocytosis etc. add Chen et al, PNAS, 2022, 119 (20) e2111051119; Yao et al., JNeuroscience, 2012, 32:3778. Colasante et al, 2013, Mol. Neurobiol., 48:120.)
In light of this, we have added the following paragraph in the introduction: "Syt1 and SV2 receptors are, therefore, remarkably complementary in the SV targeting of CNTs. SV2 is an intrinsic trafficking partner (iTRAP) for Syt1, ensuring that these SV proteins are clustered and retrieved efficiently from the neuronal plasma membrane [14-17]. Syt1-SV2 iTRAP interactions [18,19], and the ability of Syt1 and SV2 to form nanoclusters at the plasma membrane [20], are considered to function as a "fail-safe" mechanism that ensures SVs are formed with the correct molecules at the required stoichiometry. BoNT/A-Hc has been shown to internalize into uncoated SVs and clathrin-coated vesicles [21-24], and both Syt1 and SV2 have been shown to interact with clathrin adaptor proteins. Syt1 has also been shown to function as a dual Ca^{2+} sensor for both endo- and exocytosis [25,26], and to specifically facilitate clathrin-mediated endocytosis, and clamp bulk endocytosis [26,27]. The cytosolic C2B domain of Syt1 has been shown to bind to endocytic adaptor protein 2 (AP-2) and stonin-2, further supporting a role for Syt1 in clathrin-dependent endocytosis [28-32], and SV2A has been shown to bind to multiple clathrin-dependent endocytosis proteins such as AP-2, epidermal growth factor receptor pathway substrate 15 (EPS15), and amphiphysin 2/ bridging integrator 1 (Bin1) [15]. SV2 and stonin-2 play a major role in regulating the amount of Syt1 in SVs [15,17]".

Discussion: The first section is repetitive and can be deleted. The rest needs to be tuned down on claims and add the discussion on technical limitations and potential alternative explanations.
We thank the reviewer for the suggestion. We have now focused the Discussion on the findings, tuned down our claims and introduced a paragraph on technical limitations and alternative explanations.

"inducing": SV2-Syt1 interact and form complexes without BoNT/A. The word "inducing" is misleading. It can be replaced with "enhancing", "increasing", "elevating", "facilitating", etc. The surprise is not that SV2-Syt1 form a complex, but that the complex formation is not saturated and can still be further promoted with BoNT/A.
We have replaced "inducing" with more appropriate wording, such as promoting and enhancing.

2. In contrast to Figures 1-3, Figures 4-5 are less relevant, overinterpreted, and some data are problematic. Syt1 is essential for SV exocytosis and endocytosis recycling, therefore it is not surprising that KD of Syt1 reduces BoNT/A and BoNT/E entry, as the entry pathway is disrupted. This part is over-exaggerated unnecessarily, without acknowledging many of the previous studies (e.g. Chen et al, PNAS, 2022, 119 (20) e2111051119; Yao et al., JNeuroscience, 2012, 32:3778; Willig et al, Nature, 2006, 440:935; Liu et al, J. Neurosci. 2009, 29:7395). There are insufficient controls to differentiate differences in the general role of Syt1 in synaptic vesicle recycling from any specific role in BoNT/A's selective uptake.

To address these points and possible caveats, we have:

- included extensive control experiments showing that the effect of Syt1 sgRNA1 knockdown (KD) on both the SV2 expression level (Appendix Figure S8 A-D) and plasma membrane localization (Appendix Figure S8 E-G) are minimal.
- demonstrated BoNT/Ai^{PSG}-At647N (a mutant that only binds to SV2) binds to the neuronal plasma membrane at similar levels in control neurons and in Syt1 sgRNA1 KD neurons, providing further support that Syt1 depletion in our CRISPRi approach does not perturb the availability of SV2 on the neuronal plasma membrane (see Appendix Figure S8 H-J).
- demonstrated that the Syt1 sgRNA1 KD does not change mPSCs frequency significantly (please see detailed reply below), demonstrating that the endocytic pathway is minimally disrupted by the partial knockdown.
- shown that the number of Syt1 plasma membrane nanoclusters is elevated by the toxin and that this is highly dependent on the tripartite PSG-Syt1-SV2 binding interactions. This potentiation of clustering promoted by by the toxin suggests that Syt1 is an essential sorting factor, upstream of endocytosis. Whether Syt1 plays a downstream role in endocytosis of the toxin, remains to be studied.

As this is an important topic, we have included the following additional results into the manuscript:

- As requested below, we have now replaced the western blot quantification of SV2 expression following Syt1 sgRNA1 KD with corresponding immunofluorescense quantification (Appendix Figure S8 B-D), demonstrating a small, but non-significant, decrease in SV2A levels following Syt1 sgRNA1 KD.
- To address the important issue of control experiments for the EM studies (Fig. 5D), we have included ruthenium red (RuR) EM results (Appendix Figure S8 L) which demonstrate that the membrane tubulation following Syt1 KD is cause by BoNT/Ai, and not the KD itself (please see the full explanation below).
- As requested, we have performed additional rescue experiments with Syt1^{K52A}-pH (a mutation that abolishes the PSG-Syt1 interactions at Syt1 K52 residue) in Syt1 sgRNA1 KD neurons, demonstrating that the mutated Syt1 fails to rescue the KD phenotype of decreased SNAP-25/A cleavage (Appendix Figure S7 L-N). It is worth noting that Syt1^{K52A} only carries a mutation in the luminal domain of the protein, which does not interfere with the cytoplasmic C2A and C2B domains that are important for its endocytic function. Flores *et al*., PNAS 2019 116: 18098–18108 demonstrate that the subcellular distribution of Syt1^{K52A} is similar to that of Syt1^{wt}, indicating that the recycling of the protein is not perturbed by the mutation. Together, these results indicate that the decrease in SNAP-25 cleavage following Syt1 KD is not due to disrupted entry pathway, but rather by the absence of Syt1 in the process.

Figure 4: examining cleavage of SNAP-25 is not meaningful here as SV exo-endo recycling is disrupted. It would be more meaningful to do a more careful study of BoNT/A endocytosis, using authors' high-tech approach, in the presence of Syt1 (K52A) in Syt1 KO neurons.
We performed rescue experiments in Syt1 sgRNA1 KD background with Syt1^{K52A}-pH, demonstrating that the mutant with abolished binding to PSGs was less effective in rescuing the CRISPRi phenotype of decreased SNAP-25 cleavage than Syt1^{wt}-pH. These results have been included in Appendix Figure S7 L-N.

Figure 5: the mIPSCs data are less relevant and problematic. Syt1 KO and KD neurons should show higher mIPSC frequency, not lower, than the control (Coutney et al, Nat. Commun. 2019, 10:4076). This part is unnecessary and can be taken out.
We thank the reviewer for the comment, but we respectively disagree with the interpretation of our mPSC results for technical reasons explained below. Liu *et al*, J. Neurosci. 2009, 29:7395 demonstrate that the frequency of spontaneous mIPSCs is unchanged in Syt-I KO neurons when neurons are plated at 25,000–50,000 cells/cm^2 density. We routinely plate 100,000 neurons/ 314.16 mm^2 glass-bottom dishes (Joensuu *et al*., Nature Protocols 2017) which corresponds to a density of ~32,000 neurons/cm^2. In agreement with Liu et al., 2009, our mPSC results demonstrate that Syt1 sgRNA KD in these cultures does not significantly change the mPSC frequency. Coutney *et al*, Nat. Commun. 2019, 10:4076 report the use of 12–17 day old cultures, while we performed all our experiments at DIV 21-22 to ensure the maturity of the cultures. They seeded neurons at ~100,000 cells/cm^2 which is 3 times higher than what we use in our *in vitro* cultures. Both Liu *et al.,* 2009 and Coutney *et al*. 2019 report higher mPSC frequency when patch clamping these high-density neuronal cultures. In addition, both of these publications used Syt1 knockout (KO), whereas we chose to use Syt1 KD and not a full KO of Syt1 due to the known function of Syt1 in exo- and endocytosis. Our mPSCs therefore support the results reported in Liu *et al*, and are not comparable to those reported in Coutney *et al*. In addition, we recorded all miniature postsynaptic currents (mPSCs) rather than miniature inhibitory postsynaptic currents (mIPSCs) in our experiments, which has now been clarified throughout the manuscript. Coutney *et al*, Nat. Commun. 2019, 10:4076 show only GABAergic currents. To our knowledge, excitatory and inhibitory neurotransmitter release is equitable. However, there are more excitatory than inhibitory synapses in hippocampal neurons (~3:1 ratio), which means that a decrease in frequency due to reduced presynaptic release would be more noticeable when looking only at GABAergic currents, which would explain their smaller deviation, resulting in a significant increase.

Given that Syt1 appears to function as an endocytic sorting factor of BoNT/A on the neuronal plasma membrane, and that the toxin entry pathway is significantly perturbed by the Syt1 KD, we argue that the mPSC results support our main finding and should therefore remain in the manuscript.

The tubules in Figure 5 are interesting findings, but missing the controls on specificity. A recent study indicates the development of large tubular structures occurs in Syt1 KO neurons using HRP as a traditional lipophilic label (Chen et. al 2022, PNAS, PMID: 35537054), indicating these effects are unrelated to botulinum neurotoxin, but ascribed to loss of synaptic endocytosis and increase of large-sized endocytosis.
In Chen *et. al*., PNAS 2022 119:1-12, the authors used 100 mM K^+ external solution supplemented with 10 mg/mL HRP at 37 °C for 2 min before fixation for EM. Based on these experiments, they conclude that Syt1 KO decreases HRP-labelled small vesicles. They further demonstrated the formation of large endosomal structures in Syt1 KO neurons, but no membrane tubulation. Syt1 C2A–C2B fragments were demonstrated to convert liposomes into

long, thin lipid tubules in the presence of 1 mM Ca^{2+}, which simply demonstrates that the protein has a propensity to promote membrane tubulation.

To addess the important issue of specificity of toxins inducing the tubular phenotype observed in EM (Fig. 5D), we hypothesised that if Syt1 depletion alone induces tubulation in neurons, without BoNT/A, we should be able to observe the tubules following Syt1 sgRNA KD using ruthenium red (RuR) plasma membrane staining, which produces an electron-dense precipitate that can be observed in EM (see Blaquet, Histochemistry 1976 47:175-89). We prepared EM samples of Syt1 sgRNA1 KD hippocampal neurons, and stimulated the neurons with high K^+ buffer with and without 100 pM unlabelled BoNT/Ai^{wt}. We then stained the neuronal plasma membrane with RuR for EM. In the absence of toxin, we did not observe RuR-positive tubulations/invaginations of the plasma membrane in the Syt1 KD neurons (Appendix Figure S8 L). In contrast, in the presence of toxin, elongated tubular structures stained with RuR could be detected following Syt1 KD, indicating that the tubulation effect was specifically induced by the toxin in Syt1 KD neurons (Appendix Figure S8 L). It is worth noting though that RuR would only stain the tubular membrane if the tubule remained connected to the plasma membrane. It is therefore possible that some of the formed tubules could have lost their connection to the plasma membrane, and that they were not detected with RuR staining. However, it is unlikely that all the tubules would have lost the connection to the plasma membrane as we regularly observed the toxin-containing tubules originating from the plasma membrane (Figure S8 K).

Syt1 KD in cultured hippocampal neurons by DIV21-22 days will reduce the amount of SV2, and the total vesicle numbers (e.g. Liu et al, J. Neurosci. 2009, 29:7395).
We thank the reviewer for raising this important point. Although the SV2A/B KO has been shown to lead to partial loss and missorting of Syt1 to the neuronal surface (Kaempf *et al*., PNAS 2015, 112:7297-7302), we are not aware of any study which shows that Syt1 KD alters the expression level of SV2. While Liu *et al*, J. Neurosci. 2009, 29:7395 does demonstrate that Syt1 KD decreases the total number of synaptic vesicles in hippocampal neurons, it does not address the amount of SV2 following Syt1 KD. However, as this is an important issue, we have now quantified the level of SV2 using immunofluorescence labelling of endogenous SV2A following Syt1 sgRNA1 KD, as suggested (please see the response to the next question and the results included in Appendix Figure S8 B-D).

The authors' western blot data on this point is problematic (Fig. S8). As the authors showed reduction of Syt1 using imaging approaches in Figure 4, the level of SV2 needs to be validated using the same approach.
We have now included the immunofluorescence quantification of endogenous SV2A following Syt1 sgRNA KD (Appendix Figure S8 B-D), demonstrating that this does not reduce SV2A levels significantly. Our results also demonstrate that, following Syt1 KD, BoNT/Ai^{PSG}-At647N binding on neurons remains unaltered, indicating that there are sufficient numbers of SV2 receptors present on the neuronal plasma membrane with which the toxin can bind to. As requested, we have replaced the western blot analysis shown in Appendix Figure S8 B-D, with the the immunofluorescence analysis.

One solution is to expand figures 1-3 into more figures and minimize figures 4-5, to re-focus on the strong part of this work, rather than the Syt1 KD part.
We appreciate the comment, but respectively disagree. We acknowledge that perturbations that reduce exocytosis can indirectly affect endocytosis (Jorgensen *et al*., 1995 *Nature* 378, 196–199; Poskanzer, *et al*., 2003 *Nature* 426, 559–563 and Nicholson-Tomishima & Ryan 2004 *PNAS* 101, 16648–16652). Syt1 is required for normal rates of synaptic vesicle endo- and

exocytosis, which could indicate that the kinetic defects observed during endocytosis in Syt1 KO neurons could be due to defective exocytosis. Edwin Chapman's laboratory has demonstrated an uncoupled function of Syt1 in exo- and endocytosis in mouse neurons by re-targeting the protein or via mutagenesis of the Syt1 tandem C2 domains (Yao *et al*., 2023 Nat Neurosci 15:243–249). The effects of these manipulations on exo- and endocytosis were analyzed using electrophysiology, in conjunction with optical imaging of the vesicle cycle, and their results indicate that changes in exocytosis do not always result in changes in endocytosis, at least in mouse hippocampal neurons, and that Syt1 is directly involved in endocytosis. Notably, both C2A and C2B were able to function as Ca^{2+} sensors for endocytosis. Our Syt1 KD results (Figures 4 and 5) are central, as they demonstrate an upstream endocytic targetting role for Syt1 in the neurointoxication process of BoNT/A. These results demonstrate that Syt1 mediates the synaptic vesicle targeting of the toxin, rather than being a mere bystander to the process. We agree that perturbations of key synaptic vesicle proteins such as Syt1 or SV2 will affect exo- and endocytosis rates in neurons. However, our data demonstrate that the Syt1 sgRNA1 KD, instead of full KO, does not perturb the physiological function of Syt1 significantly, and that neither the SV2 mRNA, protein levels, nor the recycling of SV2, are perturbed by the KD of Syt1.

3. Figure 2D: the impact of BoNT/A on Syt1 mobility is rather small (0.29 to 0.26?). The authors need to re-check it by averaging technical replicates within a biological replicate and replot and compare the mean of each biological replicate. Additional repeats (ideally done in blind) might be needed to confirm the results. Each technical replicate is presented as an average diffusion coefficient of hundreds to thousands of single-molecule tracks with large variability, as seen in Fig. 2D. The mobile-to-immobile (M/IMM) ratio shown in Appendix Figure S5 C better captures the BoNT/Ai^{wt}-induced effect on Syt1^{wt} single-molecule mobility. In support of these data, we also performed uPAINT experiments (surface molecule tracking) using a probe that selectively recognizes an endogenous luminal epitope of Syt1 that is exposed to the extracellular space following synaptic vesicle fusion with the plasma membrane. In these experiments, we detected a similar decrease in the average diffusion coefficient (μm^2 s^{-1}) of endogenous Syt1 in the presense of BoNT/Ai^{wt} on the plasma membrane. The generation and validation of this probe is being prepared for a separate manuscript, and we are unable to include the data in this manuscript. We agree with the reviewer that the effect size is small, but it is significant across all tested experimental paradigms. Considering that uPAINT imaging records the nanoscale changes (localization precision 30-40 nm; Joensuu *et al*., Nat Protocols 2017, 12:2590-2622) in the single-molecule mobility of the proteins, and that we use minimal amounts of toxin (100 pM to 1 nM), we expect the effect sizes to be in this range (one toxin molecule engages with one PSG-Syt1-SV2 nanocluster). For example, the addition of a single receptor in a cluster can control whether or not a receptor and bound cargo are internalized efficiently, highlighting how nanoscale changes alter biological processes (See Salavessa et al., PNAS 2021 118:e2024893118).

Other minor points
Introduction:
2nd paragraph line 1: missing a blank before E. We have now corrected this typographical error.

2nd paragraph line 4: of glycoproteins. This ha snow been corrected.

3rd paragraph line 1-3: many previous studies utilized full length active toxins. We apologize for the imprecise wording. Our intention was to refer to BoNT/A studies performed using super-resolution imaging. We have modified the the text in the introduction as follows: "Because of their small size, Syt1 and SV2 nanoclusters and the nanoscale mobility of BoNT/A can only be detected in live neurons using super-resolution single-molecule imaging which allows the investigation of the neurointoxication with high spatiotemporal resolution. One of the major limitations of published work on the binding, endocytosis and trafficking of BoNT/A using live-cell super-resolution imaging has been the reliance on atoxic 50 kDa receptor binding domains, the H_C fragments at concentrations far exceeding pathophysiological levels[33,34]. Here, we took advantage of single- and dual-colour super-resolution imaging, in particular live-cell single-molecule tracking, in cultured hippocampal neurons to fully characterize the intoxication mechanism of intact BoNT/A at picomolar concentrations from plasma membrane binding to selective endocytosis into SVs.".

Results:
Line 1-3: Authors need to tune down their claims and delete line 1-3. We thank the reviewer for the comment, and have modified the sentences in the Results section as requested.

Page 3, last line and Page 4, 3rd paragraph, line 4-9: as the double KO showed no further reduction in binding compared with PSG KO, there is lack of evidence for SV2-mediated binding here. The author cannot assume that these binding observed for PSK mutant is mediated by SV2.
This is an important topic, that we have now expanded in the Discussion: "Our uPAINT single-molecule imaging of BoNT/Ai^{PSG}-At647N and BoNT/Ai^{SV2}-At647N revealed significantly decreased binding of the toxin on the neuronal plasma membrane compared to wildtype toxin. Unexpectedly, BoNT/Ai^{PSG,SV2}-At647N binding to the plasma membrane did not show a further decrease compared to the other receptor binding mutant holotoxins. One explanation could be that the slightly elevated At647N-labelling of BoNT/Ai^{PSG,SV2} compared to the other mutant holotoxins (Appendix Figures S1 E and S2 A) increases the positive charge of the toxin molecule, thereby potentially increasing its non-specific binding of the toxin. Our analysis on the binding of At647N-labelled mutant toxins to glass-bottom dishes showed no differences in the number of bound toxins (data not shown), indicating that the increased At647N labelling was not contributing to the BoNT/Ai^{PSG,SV2}-At647N binding. Additional factors, such as FGFR3 [83] which interacts with SV2 [84], have also been suggested to have a role in BoNT/A neurointoxication, which could offer an alternative explanation as to why the BoNT/Ai mutant, with abolished binding to both of its primary receptors, was still able to bind neuronal plasma membrane. However, based on our results, potential engagement with additional receptors without PSG and SV2 binding resulted in unsuccessful endocytic uptake of the toxin into SVs, and therefore the role of additional factors remains to be determined".

Page 4, line 16: what is "cytochemically"? We have defined and explained the cytochemical staining in more detail in the Materials and Methods "HRP cytochemistry and electron microscopy" section.

BoNT/A is in SV: needs to cite previous paper: Colasante et al, 2013, Mol. Neurobiol., 48:120. The reference has been added.

Page 7 line: description of BoNT/E was repeated. Thank you for noticing the error. We have corrected the mistake.

What is this TagBFP2?
TagBFP2 was described in the Results section as “tag blue fluorescent protein 2”. It is a fluorescent protein that can be excited with ultraviolet light. We introduced TagBFP2 in the lentivirus vector expression cassette under a separate promoter from non-targetting sgRNA or Syt1 sgRNA1-3, which allowed us to use automated image analysis to identify and segment lentiviral-transduced neurons. We have now clarified this point more clearly in the Materials and Methods “Automated image segmentation and fluorescence intensity analysis” section.

"which fits with earlier findings that Syt1 KD perturbs the Ca2+-sensor function of Syt1 in evoked synchronous release": This sentence does not make much sense here - there is no study of evoked release here. We have removed the sentence.

Needs to cite references for Stonin and AP2. We have included the following text in the Introduction, elucidating the interaction between Syt1 and SV2 with AP-2 and stonin-2: “The cytosolic C2B domain of Syt1 has been shown to bind to endocytic adaptor proteins AP-2 and stonin-2, further supporting a role for Syt1 in clathrin-dependent endocytosis [28-32], while stonin-2 and SV2 have been shown to have overlapping functions in sorting Syt1 into SVs [17].

Syt1 is not a "must", but rather facilitates the endocytosis efficacy. Our results show a direct correlation between the expression level of Syt1, and BoNT/A-induced cleavage of SNAP-25. Over-expression of Syt1 in naïve neurons increased the level of SNAP-25 cleavage, indicating that Syt1 expresion is rate-limiting to BoNT/A neurointoxication.

2nd Revision - Editorial Decision 4th Apr 2023

Dear Dr. Meunier,

Thank you for submitting a revised version of your manuscript. Your study has now been seen by one of the original referees, who finds that their previous concerns have been addressed in a satisfactory manner and now recommends publication of the manuscript after minor textual edits. I will look into the possible title adjustments in the coming days. Meanwhile, there remain also a few editorial points that have to be addressed before I can extend formal acceptance of the manuscript:

1. Our publisher has done their pre-publication check on your manuscript. I have attached the file here. Please take a look at the word file and the comments regarding the figure legends and respond to the issues. Please also use this version when you resubmit the revised version.
2. Please submit up to five keywords.
3. There are references to "data not shown" on pages 11 and 27. According to our policy, which does not permit references to "data not shown", please include this information in the Appendix. Please see also https://www.embopress.org/page/journal/14602075/authorguide#unpublisheddata.
4. Please rename movies into Movie EV1-EV5 and update the callouts. Please remove the movie legends form the manuscript file and zip together with each movie file as a plain text README file.
5. Please remove the Appendix Figure legends from the main manuscript file and add to the Appendix.
6. Please check Appendix Figure labelling - Appendix Figure S8 appears to be mislabelled.
7. Please rename the "Conflict of Interest" section into "Disclosure Statement and Competing Interests".
8. CRediT has replaced the traditional author contributions section because it offers a systematic, machine-readable author contributions format that allows for more effective research assessment. Please remove the Authors Contributions from the manuscript and use the free text boxes beneath each contributing author's name in our online submission system to add specific details on the author's contribution. More information is available in our guide to authors.
9. Please update the reference list according to The EMBO Journal style - where there are more than 10 authors on a paper, the first 10 should be listed, followed by 'et al.' Please see further information here: https://www.embopress.org/page/journal/14602075/authorguide#referencesformat
10. Papers published in The EMBO Journal are accompanied online by a 'Synopsis' to enhance discoverability of the manuscript. It consists of A) a short (1-2 sentences) summary of the findings and their significance, B) 3-4 bullet points highlighting key results and C) a synopsis image that is 550x300-600 pixels large (width x height, jpeg or png format). You can either show a model or key data in the synopsis image. Please note that the image size is rather small and that text needs to be readable at the final size. Please send us this information together with the revised manuscript.

Please let me know if you have any questions regarding any of these points. You can use the link below to upload the revised files.

Thank you again for giving us the chance to consider your manuscript for The EMBO Journal. I look forward to receiving the final version.

With best regards,

Ieva

Ieva Gailite, PhD
Senior Scientific Editor
The EMBO Journal
Meyerhofstrasse 1
D-69117 Heidelberg
Tel: +4962218891309
i.gailite@embojournal.org

Further information is available in our Guide For Authors: https://www.embopress.org/page/journal/14602075/authorguide

We realize that it is difficult to revise to a specific deadline. In the interest of protecting the conceptual advance provided by the work, we recommend a revision within 3 months (3rd Jul 2023). Please discuss the revision progress ahead of this time with the editor if you require more time to complete the revisions.

Referee #2:

The revision is overall satisfactory.
Minor points:
1. Title: either spell out "PSG-Syt1-SV2" or delete it? change to "recycling synaptic vesicles'?
2. Abstract line 3: suggest to add one transition sentence such as "but the relationship between PSG and SV2 remains unknown", or "whether and how PSG may coordinate with SV2 for BoNT/A binding and entry remains unknown"?
3. Abstract line 4: recycling synaptic vesicles?
4. Introduction, 3rd paragraph: needs to introduce SV2A.
5. Page 3, line 1: "Hc" Line 15: change "loss" to "reduction".
6. Page 5, line 8: add something like "at pM toxin concentrations". Line 9, this sub-title can be re-worded.

April 18th, 2023

Re: EMBOJ-2022-112095R1

Dear Dr Gailite,

Thank you for your email, and for the opportunity to revise our manuscript. On behalf of our collaborators, please find enclosed the revised version of our manuscript titled "A tripartite PSG-Syt1-SV2 plasma membrane nanocluster sorts BoNT/A into synaptic vesicles" for your consideration as a research article in EMBO Journal. Please find our detailed responses to the Editor point and reviewer comments below.

Responses to Editorial points:

1. Our publisher has done their pre-publication check on your manuscript. I have attached the file here. Please take a look at the word file and the comments regarding the figure legends and respond to the issues. Please also use this version when you resubmit the revised version.
All the requested details have been added in tracking mode. We have also added the missing "Data information" on Appendix Figure legends.

2. Please submit up to five keywords.
BoNT/A, Synaptotagmin-1, SV2, Polysialogianglioside, endocytosis

3. There are references to "data not shown" on pages 11 and 27. According to our policy, which does not permit references to "data not shown", please include this information in the Appendix. Please see also https://www.embopress.org/page/journal/14602075/authorguide#unpublisheddata.
Page 11 reference to "data not shown" is now replaced with Appendix Figure S8 L. The respective information pertaining to the above-mentioned experiments has been included in the Materials and Methods (page 16), and Appendix Figure S8 figure legend. The reference of the "not shown" on page 27 refers to the different output options of the NASTIC Cluster analysis tool as examples. We have now modified this section by solely referencing to the output options that have been included in the manuscript (i.e. "…clustering metrics such as percentage of clustered tracks and co-clustered tracks, as well as identifying spatial "hotspots" of repeated cluster formation over the acquisition period.").

4. Please rename movies into Movie EV1-EV5 and update the callouts. Please remove the movie legends form the manuscript file and zip together with each movie file as a plain text README file.
Movies have been renamed as requested, callouts updated and zipped together with README files.

5. Please remove the Appendix Figure legends from the main manuscript file and add to the Appendix.
Appendix Figure Legends have been moved to the Appendix.

6. Please check Appendix Figure labelling - Appendix Figure S8 appears to be mislabelled.
The figure legend has been corrected. We have also included the missing information on sample sizes throughout the Appendix Figure legends.

7. Please rename the "Conflict of Interest" section into "Disclosure Statement and Competing Interests".
The text has been modified as requested.

8. CRediT has replaced the traditional author contributions section because it offers a systematic, machine-readable author contributions format that allows for more effective research assessment. Please remove the

Authors Contributions from the manuscript and use the free text boxes beneath each contributing author's name in our online submission system to add specific details on the author's contribution. More information is available in our guide to authors.
Author contribution have been removed from the manuscript and placed in the Credit section.

9. Please update the reference list according to The EMBO Journal style - where there are more than 10 authors on a paper, the first 10 should be listed, followed by 'et al.' Please see further information here: https://www.embopress.org/page/journal/14602075/authorguide#referencesformat
The references have been updated as requested.

10. Papers published in The EMBO Journal are accompanied online by a 'Synopsis' to enhance discoverability of the manuscript. It consists of A) a short (1-2 sentences) summary of the findings and their significance, B) 3-4 bullet points highlighting key results and C) a synopsis image that is 550x300-600 pixels large (width x height, jpeg or png format). You can either show a model or key data in the synopsis image. Please note that the image size is rather small and that text needs to be readable at the final size. Please send us this information together with the revised manuscript.
A) Summary: Botulinum neurotoxin type A (BoNT/A) coincidentally binds to a preassembled PSG-synaptotagmin 1 (Syt1) complex and SV2 on the neuronal plasma membrane, facilitating Syt1-SV2 nanoclustering, thereby controlling the endocytic sorting of the toxin into synaptic vesicles.

B) Bullet points:

- The targeting of BoNT/A to synaptic vesicle in primary hippocampal neurons depends on a tripartite polysialoganglioside (PSG) - synaptotagmin 1 (Syt1) – synaptic vesicle protein 2 (SV2) nanocluster on the plasma membrane.
- BoNT/A must coincidentally bind to a preassembled PSG-Syt1 complex and SV2 to facilitate Syt1-SV2 nanoclustering on the neuronal plasma membrane, leading to endocytic sorting of the toxin into synaptic vesicles.
- Syt1 is required for the BoNT/A and BoNT/E neurointoxication as demonstrated by Syt1 knockdown which decrease the toxin-induced SNAP-25 cleavage, suggesting a general mechanism of entry for clostridial botulinum neurotoxins.

C) Synopsis image:
Synopsis image with BioRender Publication license have been uploaded into the submission system.

Responses to Referee #2 points:

1. Title: either spell out "PSG-Syt1-SV2" or delete it? change to "recycling synaptic vesicles'?
Our title is currently "A tripartite PSG-Syt1-SV2 plasma membrane nanocluster sorts BoNT/A into synaptic vesicles". We would be very happy with "A tripartite polysialoganglioside - synaptotagmin-1 - synaptic vesicle protein 2 plasma membrane nanocluster sorts BoNT/A into synaptic vesicles", but it is longer than the allowed title length in EMBO J. We would be happy to hear whether this is possible.

2. Abstract line 3: suggest to add one transition sentence such as "but the relationship between PSG and SV2 remains unknown", or "whether and how PSG may coordinate with SV2 for BoNT/A binding and entry remains unknown"?
We are happy to add the following sentence in the abstract: "Whether and how PSGs and SV2 may coordinate other proteins for BoNT/A recruitment and internalization remains unknown.". This sentence has been added into the manuscript file, but not in the online submission system, as the addition will result the Abstract being over 175 words. We hope the Editor can allow inclusion of the requested sentence into the Abstract.

3. Abstract line 4: recycling synaptic vesicles?
Although it is clear that BoNT/A enter endocytic synaptic vesicles, we have not formerly demonstrated that the hijacked vesicles are part of the recycling pool of synaptic vesicles. This is why we have avoided reference to "recycling" in the title and throughout the manuscript.

4. Introduction, 3rd paragraph: needs to introduce SV2A.
Introduction to different SV2 isoforms has been added on page 2.

5. Page 3, line 1: "Hc" Line 15: change "loss" to "reduction".
“HC” has been changed to “Hc” on page 3. On page 3, “loss” has been replaced with “reduction”.

6. Page 5, line 8: add something like "at pM toxin concentrations". Line 9, this sub-title can be re-worded.
Requested addition of concentration has been added on page 5 line 16. The subtitle on P5 has been reworded to “BoNT/A-induced SV2A immobilization at the plasma membranerequires Syt1”. We hope that these modifications are satisfactory and that our manuscript will be officially accepted for publication in EMBO J.

We look forward to hearing from you.

Yours sincerely,
Merja Joensuu, PhD and Frederic A. Meunier, PhD

3rd Revision - Editorial Decision 28th Apr 2023

Dear Fred and Merja,

Thank you for addressing the final editorial issues, and I apologise for the delay in communicating the decision due to the Easter holidays and the resulting backlog. I am now pleased to inform you that your manuscript has been accepted for publication.

Before we forward your manuscript to our publishers, there remain a couple of points that need addressing:
1. You have indicated that the synopsis image was prepared with BioRender. As requested in their license, please add a small text in the bottom corner of the image stating, "Created with BioRender.com".
2. I would like to propose a couple of changes in the article title, abstract and synopsis, mainly to shorten them and increase the accessibility to our more general readership. I have also written a short blurb that will accompany the title of your manuscript in our online table of contents. Please take a look at the text below and in the attached manuscript text file and let me know if any corrections are necessary.

Proposed new title:
Nerve end targeting of botulinum neurotoxin BoNT/A requires a tripartite PSG-Syt1-SV2 plasma membrane nanocluster for synaptic vesicle entry

Blurb:
Live-cell super-resolution imaging and electron microscopy of botulinum toxin-infected hippocampal neurons reveal the mechanism for its synaptic vesicle hijacking and neurointoxication.

Synopsis:
Synaptic targeting of botulinum neurotoxins is mediated by interaction with toxin-type-specific receptors and complex gangliosides, such as polysialoganglioside (PSG). This study shows that botulinum neurotoxin type A (BoNT/A) uptake and sorting into synaptic vesicles requires a tripartite nanocluster on the neuronal plasma membrane.
• Live-cell super-resolution and electron microscopy in primary hippocampal neurons shows BoNT/A synaptic vesicle targeting depends on a tripartite PSG-synaptotagmin 1 (Syt1)-synaptic vesicle protein 2 (SV2) nanocluster on the plasma membrane.
• Coincidental binding of BoNT/A to a preassembled PSG-Syt1 complex and SV2 facilitates Syt1-SV2 nanoclustering on the neuronal plasma membrane and endocytic sorting of the toxin into synaptic vesicles.
• Syt1 is required for the BoNT/A and BoNT/E neurointoxication, suggesting a general mechanism of entry for clostridial botulinum neurotoxins.

I have also forwarded your beautiful cover suggestion to my colleague Daniel Klimmeck, who is responsible for cover image selection at our journal.

Please note that it is EMBO Journal policy for the transcript of the editorial process (containing referee reports and your response letter) to be published as an online supplement to each paper. If you do NOT want this, you will need to inform the Editorial Office via email immediately. More information is available here:
https://www.embopress.org/page/journal/14602075/authorguide#transparentprocess

Your manuscript will be processed for publication in the journal by EMBO Press. Manuscripts in the PDF and electronic editions of The EMBO Journal will be copy edited, and you will be provided with page proofs prior to publication. Please note that supplementary information is not included in the proofs.

You will be contacted by Wiley Author Services to complete licensing and payment information. The required 'Page Charges Authorization Form' is available here: https://www.embopress.org/pb-assets/embo-site/tej_apc.pdf - please download and complete the form and return to embopressproduction@wiley.com

EMBO Press participates in many Publish and Read agreements that allow authors to publish Open Access with reduced/no publication charges. Check your eligibility: https://authorservices.wiley.com/author-resources/Journal-Authors/open-access/affiliation-policies-payments/index.html

Should you be planning a Press Release on your article, please get in contact with embojournal@wiley.com as early as possible, in order to coordinate publication and release dates.

If you have any questions, please do not hesitate to call or email the Editorial Office. Thank you for this contribution to The EMBO Journal and congratulations on a successful publication!

Best wishes,

Ieva

Ieva Gailite, PhD
Senior Scientific Editor
The EMBO Journal
Meyerhofstrasse 1
D-69117 Heidelberg
Tel: +4962218891309
i.gailite@embojournal.org

EMBO Press Author Checklist

Corresponding Author Name: Merja Joensuu and Frederic A Meunier
Journal Submitted to: The EMBO Journal
Manuscript Number: EMBOJ-2022-112095R-Q

USEFUL LINKS FOR COMPLETING THIS FORM
The EMBO Journal - Author Guidelines
EMBO Reports - Author Guidelines
Molecular Systems Biology - Author Guidelines
EMBO Molecular Medicine - Author Guidelines

Reporting Checklist for Life Science Articles (updated January
This checklist is adapted from Materials Design Analysis Reporting (MDAR) Checklist for Authors. MDAR establishes a minimum set of requirements in transparent reporting in the life sciences (see Statement of Task: 10.31222/osf.io/9sm4x). Please follow the journal's guidelines in preparing your
Please note that a copy of this checklist will be published alongside your article.

Abridged guidelines for figures
1. Data
The data shown in figures should satisfy the following conditions:
- the data were obtained and processed according to the field's best practice and are presented to reflect the results of the experiments in an accurate and unbiased manner.
- ideally, figure panels should include only measurements that are directly comparable to each other and obtained with the same assay.
- plots include clearly labeled error bars for independent experiments and sample sizes. Unless justified, error bars should not be shown for technical
- if n<5, the individual data points from each experiment should be plotted. Any statistical test employed should be justified.
- Source Data should be included to report the data underlying figures according to the guidelines set out in the authorship guidelines on Data

2. Captions
Each figure caption should contain the following information, for each panel where they are relevant:
- a specification of the experimental system investigated (eg cell line, species name).
- the assay(s) and method(s) used to carry out the reported observations and measurements.
- an explicit mention of the biological and chemical entity(ies) that are being measured.
- an explicit mention of the biological and chemical entity(ies) that are altered/varied/perturbed in a controlled manner.
- the exact sample size (n) for each experimental group/condition, given as a number, not a range;
- a description of the sample collection allowing the reader to understand whether the samples represent technical or biological replicates (including how many animals, litters, cultures, etc.).
- a statement of how many times the experiment shown was independently replicated in the laboratory.
- definitions of statistical methods and measures:
  - common tests, such as t-test (please specify whether paired vs. unpaired), simple χ2 tests, Wilcoxon and Mann-Whitney tests, can be unambiguously identified by name only, but more complex techniques should be described in the methods section;
  - are tests one-sided or two-sided?
  - are there adjustments for multiple comparisons?
  - exact statistical test results, e.g., P values = x but not P values < x;
  - definition of 'center values' as median or average;
  - definition of error bars as s.d. or s.e.m.

Please complete ALL of the questions below.
Select "Not Applicable" only when the requested information is not relevant for your study.

Materials

Newly Created Materials	Information included in the manuscript?	In which section is the information available? (Reagents and Tools Table, Materials and Methods, Figures, Data Availability Section)
New materials and reagents need to be available; do any restrictions apply?	Yes	Data and materials availability

Antibodies	Information included in the manuscript?	In which section is the information available? (Reagents and Tools Table, Materials and Methods, Figures, Data Availability Section)
For **antibodies** provide the following information:<br>- Commercial antibodies: RRID (if possible) or supplier name, catalogue number and or/clone number<br>- Non-commercial: RRID or citation	Yes	Materials and Methods, and Table EV1

DNA and RNA sequences	Information included in the manuscript?	In which section is the information available? (Reagents and Tools Table, Materials and Methods, Figures, Data Availability Section)
**Short novel DNA or RNA including primers, probes**: provide the sequences.	Yes	Materials and Methods, Tables EV1-3

Cell materials	Information included in the manuscript?	In which section is the information available? (Reagents and Tools Table, Materials and Methods, Figures, Data Availability Section)
**Cell lines:** Provide species information, strain. Provide accession number in repository **OR** supplier name, catalog number, clone number, and/**OR** RRID.	Yes	Materials and Methods, and Table EV1
**Primary cultures:** Provide species, strain, sex of origin, genetic modification status.	Yes	Materials and Methods
Report if the cell lines were recently **authenticated** (e.g., by STR profiling) and tested for mycoplasma contamination.	Not Applicable	

Experimental animals	Information included in the manuscript?	In which section is the information available? (Reagents and Tools Table, Materials and Methods, Figures, Data Availability Section)
**Laboratory animals or Model organisms:** Provide species, strain, sex, age, genetic modification status. Provide accession number in repository **OR** supplier name, catalog number, clone number, **OR** RRID.	Yes	Materials and Methods
**Animal observed in or captured from the field:** Provide species, sex, and age where possible.	Not Applicable	
Please detail **housing and husbandry conditions**.	Yes	Materials and Methods

Plants and microbes	Information included in the manuscript?	In which section is the information available? (Reagents and Tools Table, Materials and Methods, Figures, Data Availability Section)
**Plants:** provide species and strain, ecotype and cultivar where relevant, unique accession number if available, and source (including location for collected wild specimens).	Not Applicable	
**Microbes:** provide species and strain, unique accession number if available, and source.	Yes	Materials and Methods

Human research participants	Information included in the manuscript?	In which section is the information available? (Reagents and Tools Table, Materials and Methods, Figures, Data Availability Section)
If collected and within the bounds of privacy constraints report on age, sex and gender or ethnicity for all study participants.	Not Applicable	

Core facilities	Information included in the manuscript?	In which section is the information available? (Reagents and Tools Table, Materials and Methods, Figures, Data Availability Section)
If your work benefited from core facilities, was their service mentioned in the acknowledgments section?	Yes	Acknowledgements

Design

Study protocol	Information included in the manuscript?	In which section is the information available? (Reagents and Tools Table, Materials and Methods, Figures, Data Availability Section)

If study protocol has been **pre-registered, provide DOI in the manuscript**. For clinical trials, provide the trial registration number **OR** cite DOI.	Not Applicable	
Report the **clinical trial registration number** (at ClinicalTrials.gov or equivalent), where applicable.	Not Applicable	

Laboratory protocol	Information included in the manuscript?	In which section is the information available? (Reagents and Tools Table, Materials and Methods, Figures, Data Availability Section)
Provide DOI OR other citation details if **external detailed step-by-step protocols** are available.	Yes	Materials and Methods

Experimental study design and statistics	Information included in the manuscript?	In which section is the information available? (Reagents and Tools Table, Materials and Methods, Figures, Data Availability Section)
Include a statement about **sample size** estimate even if no statistical methods were used.	Yes	Materials and Methods, and Figure Legends
Were any steps taken to minimize the effects of subjective bias when allocating animals/samples to treatment (e.g. **randomization procedure**)? If yes, have they been described?	Yes	Materials and Methods.
Include a statement about **blinding** even if no blinding was done.	Not Applicable	
Describe **inclusion/exclusion criteria** if samples or animals were excluded from the analysis. Were the criteria pre-established?<br><br>If sample or data points were omitted from analysis, report if this was due to attrition or intentional exclusion and provide justification.	Yes	Materials and Methods
For every figure, are **statistical tests** justified as appropriate? Do the data meet the assumptions of the tests (e.g., normal distribution)? Describe any methods used to assess it. Is there an estimate of variation within each group of data? Is the variance similar between the groups that are being statistically compared?	Yes	Materials and Methods

Sample definition and in-laboratory replication	Information included in the manuscript?	In which section is the information available? (Reagents and Tools Table, Materials and Methods, Figures, Data Availability Section)
In the figure legends: state number of times the experiment was **replicated** in laboratory.	Yes	Figure Legends
In the figure legends: define whether data describe **technical or biological replicates**.	Yes	Figure Legends

Ethics

Ethics	Information included in the manuscript?	In which section is the information available? (Reagents and Tools Table, Materials and Methods, Figures, Data Availability Section)
Studies involving **human participants**: State details of **authority granting ethics approval** (IRB or equivalent committee(s), provide reference number for approval.	Not Applicable	
Studies involving **human participants**: Include a statement confirming that **informed consent** was obtained from all subjects and that the experiments conformed to the principles set out in the WMA Declaration of Helsinki and the Department of Health and Human Services Belmont Report.	Not Applicable	
Studies involving **human participants:** For publication of **patient photos**, include a statement confirming that consent to publish was obtained.	Not Applicable	
Studies involving experimental **animals**: State details of **authority granting ethics approval** (IRB or equivalent committee(s), provide reference number for approval. Include a statement of compliance with ethical regulations.	Yes	Materials and Methods
Studies involving **specimen and field samples:** State if relevant **permits** obtained, provide details of authority approving study; if none were required, explain why.	Not Applicable	

Dual Use Research of Concern (DURC)	Information included in the manuscript?	In which section is the information available? (Reagents and Tools Table, Materials and Methods, Figures, Data Availability Section)
Could your study fall under dual use research restrictions? Please check biosecurity documents and list of **select agents and toxins** (CDC): https://www.selectagents.gov/sat/list.htm	Yes	Active BoNT/A, BoNT/E and AbobotulinumtoxinA in Materials and Methods
If you used a select agent, is the security level of the lab appropriate and reported in the manuscript?	Yes	Materials and Methods
If a study is subject to dual use research of concern regulations, is the name of the **authority granting approval and reference number** for the regulatory approval provided in the manuscript?	Yes	Materials and Methods

Reporting

The MDAR framework recommends adoption of discipline-specific guidelines, established and endorsed through community initiatives. Journals have their own policy about requiring specific guidelines and recommendations to complement MDAR.

Adherence to community standards	Information included in the manuscript?	In which section is the information available? (Reagents and Tools Table, Materials and Methods, Figures, Data Availability Section)
State if relevant guidelines or checklists (e.g., **ICMJE, MIBBI, ARRIVE, PRISMA**) have been followed or provided.	Not Applicable	
For **tumor marker prognostic studies,** we recommend that you follow the **REMARK** reporting guidelines (see link list at top right). See author guidelines, under 'Reporting Guidelines'. Please confirm you have followed these guidelines.	Not Applicable	
For **phase II and III randomized controlled trials**, please refer to the **CONSORT** flow diagram (see link list at top right) and submit the CONSORT checklist (see link list at top right) with your submission. See author guidelines, under 'Reporting Guidelines'. Please confirm you have submitted this list.	Not Applicable	

Data Availability

Data availability	Information included in the manuscript?	In which section is the information available? (Reagents and Tools Table, Materials and Methods, Figures, Data Availability Section)
Have **primary datasets** been deposited according to the journal's guidelines (see 'Data Deposition' section) and the respective accession numbers provided in the Data Availability Section?	Yes	Data and materials availability
Were **human clinical and genomic datasets** deposited in a public access-controlled repository in accordance to ethical obligations to the patients and to the applicable consent agreement?	Not Applicable	
Are **computational models** that are central and integral to a study available without restrictions in a machine-readable form? Were the relevant accession numbers or links provided?	Yes	Materials and Methods, and Table EV1
If publicly available data were reused, provide the respective **data citations in the reference list**.	Not Applicable	
